# Expert recommendations for setting and adjusting airway pressure release ventilation based on clinical experience and basic science evidence

**DOI:** 10.3389/fmed.2026.1741129

**Published:** 2026-02-03

**Authors:** Gary F. Nieman, Jason H. T. Bates, Penny L. Andrews, Louise Rose, Joseph Shiber, Joaquin Araos, Ledoux Aurelien, Maria Madden, Toni Manougian, Josh Satalin, Tero Varpula, Hassan Al-khalisy, Manjunath Markandaya, Pedro Leme Silva, Luis Felipe da Fonseca Reis, John Downs, Luigi Camporota, Nader M. Habashi

**Affiliations:** 1 Department of Surgery, Upstate Medical University, Syracuse, NY, United States; 2 Department of Medicine, Larner College of Medicine, University of Vermont, Burlington, VT, United States; 3 Department Critical Care, University of Maryland Medical Center – R Adams Cowley Shock Trauma Center, Baltimore, MD, United States; 4 Florence Nightingale Faculty of Nursing, Midwifery & Palliative Care, King’s College London, London, United Kingdom; 5 Departments of Emergency Medicine, Neurology, and Surgery, University of Florida College of Medicine, Jacksonville, FL, United States; 6 Department of Clinical Sciences, College of Veterinary Medicine, Cornell University, Ithaca, NY, United States; 7 Department of Intensive Care, CHU HELORA - Jolimont, La Louvière, Belgium; 8 Department of Anesthesiology, Westchester Medical Center, New York Medical College, Valhalla, NY, United States; 9 Division of Intensive Care, Department of Anaesthesiology and Intensive Care, University of Helsinki and Helsinki University Hospital, Helsinki, Finland; 10 Department of Internal Medicine, Division of Pulmonary Disease, Sleep and Critical Care Medicine, East Carolina University, Greenville, NC, United States; 11 Neurocritical Care, NorthEast Georgia Medical Center, Gainesville, GA, United States; 12 Department of Critical Care Medicine, MedStar St. Mary's Hospital, Leonardtown, MD, United States; 13 Laboratory of Pulmonary Investigation, Institute of Biophysics Carlos Chagas Filho, Federal University of Rio de Janeiro, Rio de Janeiro, Brazil; 14 Postgraduate Program in Rehabilitation Sciences, Centro Universitário Augusto Mota (UNISUAM), Rio de Janeiro, Brazil; 15 Intensive Care Unit, Central Hospital of the Military Police of the State of Rio de Janeiro, Rio de Janeiro, Brazil; 16 Department of Anesthesiology, University of Florida College of Medicine, Gainesville, FL, United States; 17 Centre for Human & Applied Physiological Sciences, School of Basic & Medical Biosciences, Shepherd's House, Guy's Campus, King’s College London, London, United Kingdom; 18 Guy’s & St Thomas’ NHS Foundation Trust, London, United Kingdom

**Keywords:** airway pressure release ventilation, APRV, ARDS, consensus recommendations, TCAV, time controlled adaptive ventilation, ventilator induced lung injury, VILI

## Abstract

**Background:**

We conducted a roundtable discussion and provided evidence-based guidance on the setting and adjustment of Airway Pressure Release Ventilation (APRV) in adult patients with acute respiratory distress syndrome (ARDS).

**Methods:**

A panel of clinicians and basic scientists with extensive experience in lung physiology and using APRV was assembled to provide expert consensus guidance. The panel first established and agreed upon guiding principles for optimal APRV settings. To support consensus discussions, we then reviewed the literature on the physiological basis of APRV as a lung-protective ventilation strategy, as well as published APRV research. Finally, we held a one-day meeting and conducted robust, iterative consensus discussions using the Nominal Group Technique to reach agreement on the optimal APRV settings. This work represents an Expert Recommendation and Position Statement rather than a formal consensus guideline. The recommendations were developed through iterative expert discussions that integrated extensive clinical experience with supporting basic science evidence on time-controlled ventilation and alveolar mechanics. Recommendations were based on expert experience with APRV in the intensive care unit and supported by published animal and clinical studies.

**Results:**

Consensus on initial APRV settings for acute lung injury (ALI) such as ARDS or disorders of normal or increased elstance was as follows: set the upper airway pressure (P_High_) to either plateau or peak inspiratory pressure when transitioning from volume control or pressure control/dual control, respectively; set the duration of P_High_ (T_High_) to match the current respiratory rate on conventional ventilation; set lower airway pressure (P_Low_) to 0 cmH_2_O; and calculate duration of P_Low_ (T_Low_) using the equation Peak Expiratory Flow x 75% = Termination of Expiratory Flow. Other recommendations included titrating these settings in response to changes in lung physiology and reaching consensus on injurious APRV settings that could impair gas exchange or cause lung instability.

**Conclusion:**

The panel developed a protocol for adjusting the four APRV settings based on expert experience and solid clinical and scientific evidence for patients with ALI and ARDS, or disorders of normal or increased elastance. Optimizing the lung-protective settings in APRV mode can improve patient outcomes.

## Overview

Lung-protective ventilation aims to maintain adequate oxygenation and ventilation while reducing ventilator-induced lung injury (VILI) and related tissue damage, supported by evidence from both basic science research and clinical practice. To achieve this, 18 panelists [clinicians and basic scientists] were selected for their multidisciplinary expertise in acute respiratory distress syndrome (ARDS), mechanical ventilation, pulmonary physiology, and the clinical application of the mode airway pressure release ventilation (APRV). All those contacted had 5–35 years of experience with APRV. A summary of all panelists’ published work and biosketches is provided in Supplementary Files 1, 2.

This expert panel conference (Supplementary File 1) aimed to reach consensus on the clinical and experimental use setting and adjusting the APRV mode. The process of developing this consensus involved reviewing the evidence on the physiological basis for APRV as a lung-protective ventilation strategy, engaging in iterative discussions among panel members guided by Nominal Group Technique (NGT), and reaching final agreement on the optimal APRV settings.

**Figure 1:**
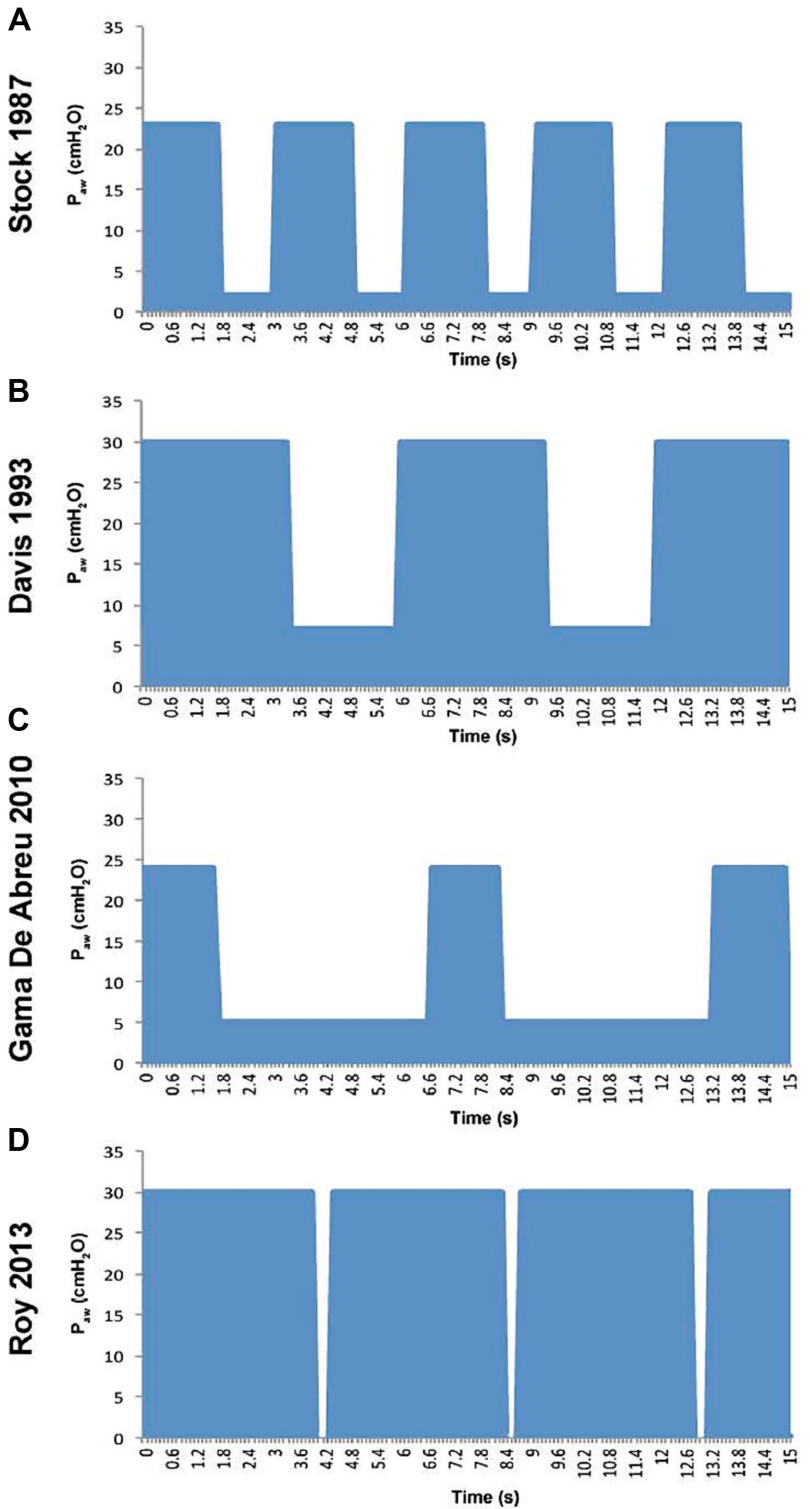
Pressure–time waveforms from four published studies using airway pressure release ventilation (APRV) demonstrate marked heterogeneity in ventilator settings. Substantial variation exists in both pressure levels (P_High_ and P_Low_) and duration of inspiration (T_High_ and T_Low_), despite all being described as APRV (2). **(A)** Stock (1987) shows 60 % continuous positive airway pressure (C_PAP_) with T_Low_ of 1.27 seconds. B) Davis (1993) decreased the respiratory rate by prolonging both (T_Low_) of 1.27 seconds. **(B)** Davis (1993) decreased the respiratory rate by prolonging both T_High_ and T_Low_. **(C)** Gama de Abreau (2010) simulated conventional ventilation with a brief T_High_ and prolonged T_Low_. In contrast, **(D)** Roy (2013) shows a personalized, brief T_Low_ and prolonged T_High_ yielding 90 % C_PAP_. A recent meta-analysis reported that in 65% of clinical trials, expiratory time (T_Low_) was set arbitrarily rather than physiologically titrated (4). Given that reducing tidal volume from 12 to 6 mL/kg has been shown to reduce ARDS mortality, it follows that how APRV is configured and adjusted is also likely to have a major impact on clinical outcomes. Consequently, trials using APRV may produce widely divergent results depending on the specific implementation of the mode.

Initially developed by Dr. John B. Downs [a faculty member at the conference] APRV was first introduced in 1987 (1). However, despite nearly four decades of use, APRV still lacks a consistent or standardized method of delivery. Analyses of its 30-year evolution have shown significant variability in its application, making it challenging to define an optimal approach (Figure 1) (2). Notably, many clinicians do not follow the most extensively studied method for setting and adjusting APRV, as highlighted in recent publications (3). A meta-analysis confirmed this heterogeneity and the ongoing lack of consensus on APRV settings (4). These findings emphasize the variability in clinical use of APRV and reflect a broader failure to incorporate key physiological and mechanistic insights into its implementation. The scientific evidence supporting the efficacy of APRV is reviewed in Supplementary File 2.

There is considerable variation in how clinicians select and modify the core four APRV parameters: upper airway pressure (P_High_), the duration of P_High_ (T_High_), lower airway pressure (P_Low_), and the duration of P_Low_ (T_Low_). Notably, 65% (13 of 20) of the studies in the clinical research set used T_Low_ arbitrarily, rather than established methods based on changes in lung pathophysiology (4).

The lack of consensus and standardization presents significant challenges for delivering and evaluating the effectiveness of APRV, as specific configurations of its settings likely influence patient outcomes (5). Although multiple small clinical trials have tested APRV, no trials have systematically analyzed outcomes by APRV setting and adjustment. Consequently, we could not perform a statistical (6) or meta-analysis (7) or support a Grading-Based recommendation (8) as used in previous analyses of patient outcomes related to APRV settings and adjustments.

To address this gap, we held a consensus conference using the NGT, bringing together experienced clinicians who have used APRV for decades and have treated thousands of ARDS patients, both as a primary and a rescue ventilation mode. Their practical expertise offers valuable insights into optimizing APRV settings in clinical practice.

## Introduction

Despite extensive investigation over the past 25 years, including the Acute Respiratory Distress Syndrome Network (ARDSNet) study, randomized controlled trials (RCTs) and meta-analyses have consistently shown no significant reduction in mortality among patients with established ARDS (15–18). This persistent mortality underscores the limitations of current protective ventilation strategies, including recruitment maneuvers (15, 16, 18) and highlights the need for alternative approaches.

The APRV mode, first introduced in 1987 (1), is one such alternative. It has strong supporting evidence from basic science demonstrating its physiological benefits (Supplementary Files 1, 2). However, the lack of standardized clinical guidelines for setting and adjusting APRV has limited its wider use and thorough evaluation. To address this, we formed an expert consensus panel comprising experienced clinicians who have extensively used APRV and scientists who have studied the mechanics of time-controlled ventilation for decades.

A major challenge in evaluating APRV’s effectiveness is the wide variation in how it is defined and implemented across clinical studies and practice, particularly with respect to ventilator settings (4). This inconsistency leads to disparate physiological and clinical outcomes, making it difficult to assess the benefits and risks of APRV accurately. This paper aims to address this issue by reaching expert consensus on the definition and use of APRV settings to ensure consistent application and interpretation in both research and clinical practice.

An extensive review has addressed misconceptions about APRV, countering claims of increased barotrauma, volutrauma, or right heart dysfunction (19). Additionally, APRV has been shown to improve secretion clearance, potentially reducing ventilator-associated pneumonia rates (20). Evidence indicates that APRV may be at least non-inferior (21) and possibly superior (22) compared to conventional ventilation strategies. Meta-analyses of RCTs favor APRV over low tidal volume ventilation (LV_T_) as a lung-protective method (23, 24). Furthermore, mechanistic data suggest that APRV reduces stress and strain in distal airspaces and improves ventilation uniformity (Supplementary File 2).

## Supporting science

The basic science literature on the mechanisms of ARDS pathophysiology and how these injuries predispose the lung to secondary VILI is reviewed in Supplementary File 3. The mechanisms by which inspiratory and expiratory times can stabilize and reopen the acutely injured lung, thereby reducing VILI, are also discussed.

To evaluate the effectiveness of APRV, tables were created listing published studies comparing APRV with other modes in both animal models and humans, as well as computational models of time-controlled ventilation (see Supplementary Files 4-7). These tables are organized into categories: clinical trials (Supplementary File 4), animal studies (Supplementary File 5), computational modeling studies (Supplementary File 6), and meta-analyses (Supplementary File 7). Whenever possible, APRV settings were extracted and included in the tables. The primary outcome parameters selected by each publication’s authors are listed in the tables. The authors’ assessments of APRV’s impact, compared to conventional ventilation controls on these outcome parameters, were recorded as Negative (A—Statistically inferior), Neutral (B—Not statistically different), or Positive (C—Statistically superior) relative to the control ventilation group. These Supplementary Files 4-7 serve as a resource compiling the published literature on APRV and time-controlled ventilation.

## Methods

### Committee composition and consensus

Our clinical panel included pulmonologists, anesthesiologists, nurses, respiratory therapists, and intensivists, all selected for their extensive experience treating patients with ARDS using APRV in both primary and rescue mechanical ventilation modes. The basic science panel comprised pulmonary physiologists and lung bioengineers who have investigated the physiological role of time-controlled lung mechanics and its pathophysiological implications.

Draft statements were developed by the organizing committee and iteratively refined through structured, moderated discussions and post-conference feedback. Consensus was pre-defined as agreement by at least 75% of panel members on a given statement following structured debate. Statements that did not meet this threshold during the initial voting round were revised based on panel feedback and re-circulated for additional discussion and voting until the predefined level of agreement was achieved or the statement was excluded.

### Formulating the questions

As a first step, the committee agreed that their focus would be on guidance for setting and adjusting the four key parameters of the APRV mode: the upper airway pressure (P_High_), the duration of P_High_ (T_High_), the lower airway pressure (P_Low_), and the duration of P_Low_ (T_Low_).

### Consensus building methods

Expert-based cooperative analysis (EbCA) represents a significant improvement in healthcare decision-making, particularly given the complexity of healthcare systems (25). EbCA uses “expert knowledge” to improve clinical decisions by combining available clinical and scientific data with insights obtained from extensive practical experience with specific treatment methods in particular patient groups (26).

Unlike “expert opinion,” which is often subjective, “expert knowledge” is grounded in accumulated clinical experience and offers a higher level of evidence (25, 26). Additionally, while evidence-based medicine has traditionally emphasized RCTs, it has been criticized for being too rigid (26). Observational studies can complement RCT results, thereby improving the quality of evidence and strengthening clinical decision-making (27).

The process began with panelists independently generating ideas in response to our predefined questions about APRV settings and adjustments. These ideas were then shared in a round-robin manner and recorded verbatim. Scientific evidence supporting APRV settings was incorporated into the discussion, providing a solid foundation for their arguments (Supplementary File 2). An iterative group discussion was held to ensure a shared understanding, during which clinical participants shared the APRV settings they found most useful in their practice.

Each of the four APRV settings was reviewed sequentially, with both its individual effects and overall impact analyzed. After each discussion, the leader (Habashi) summarized key points and identified the approach supported by the strongest clinical and scientific evidence. Once consensus was reached, the group moved on to the next setting. If consensus was not initially achieved, additional discussion was held until agreement was reached. All faculty members contributed to the final manuscript through edits and additions.

## Results

### Consensus recommendations for specific APRV settings

The consensus group developed recommendations for setting and adjusting the APRV mode, supported by strong experimental evidence (Supplementary File 2), where studies using relevant animal models have demonstrated significant improvements in lung protection and patient outcomes (28). Additionally, *in vivo* microscopy and histological analyses have documented the beneficial effects of precise inspiratory and expiratory timing on alveolar stability and the lung microenvironment (12, 13). Computational models have further clarified how changes in timing and pressure settings influence lung mechanics and stress distribution (29–34), supported by biological data analysis (35, 36).

The committee’s recommended settings aim to stabilize alveoli, maintain end-expiratory lung volume (EELV), and prevent the onset of the so-called “VILI vortex.” (37) Specifically, prolonged T_High_ facilitates gradual recruitment of collapsed lung regions, while a carefully set T_Low_ prevents derecruitment (38). These settings are designed to use time and pressure strategically to maintain alveolar stability and progressively reopen collapsed lung tissue (39).

#### Question 1: what is the optimal method for setting P_High_?

##### Background

**Figure 2:**
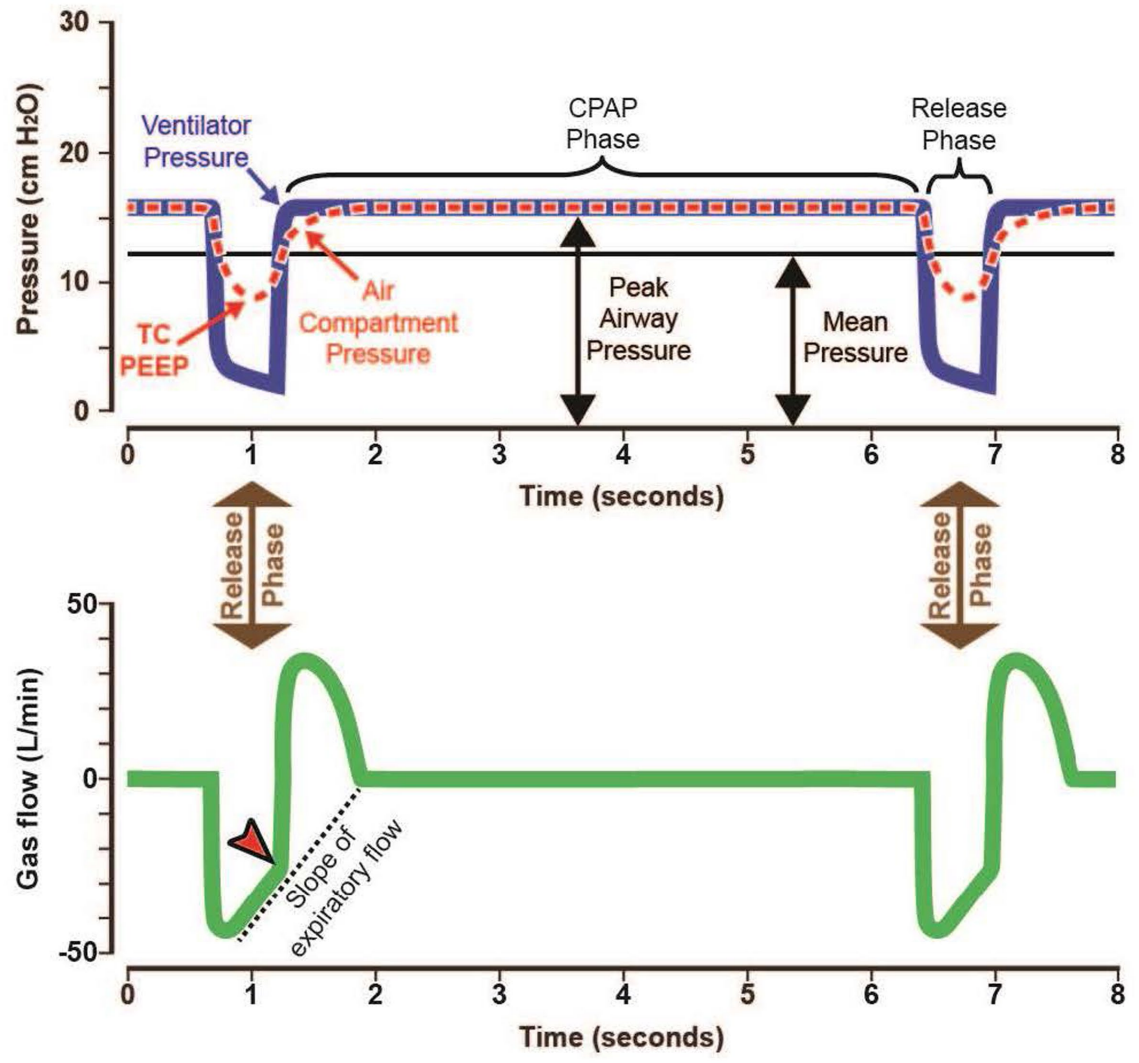
Typical airway pressure release ventilation (APRV) pressure–time and gas flow-time curves using the time-controlled adaptive ventilation TCAV method. An extended continuous airway pressure (CPAP) phase (P_High_/T_High_) and a very brief release phase (P_Low_/T_Low_) with an inhalation to exhalation (I:E) ratio as high as 12:1. The low airway pressure (P_Low_) is always set to 0 cmH_2_O. However, the release phase is so brief that the lung does not have enough time to depressurize to atmospheric pressure, so a time-controlled PEEP (TC-PEEP) is maintained. Although P_Low_ is set at 0 cmH_2_O TC-PEEP at end-expiration (red dotted line), it is approximately half of the P_High_. The T_Low_ setting is influenced by changes in respiratory system compliance (C_RS_), which is measured as the slope of the expiratory flow curve (Slope_EF_). T_Low_ is calculated by multiplying the peak expiratory flow (F_PE_) by a cofactor (75%) to determine the termination of expiratory flow (F_EE_), indicated by the red arrowhead (Equation 1; F_PE_ x 0.75 = F_EE_). As lung disease progresses, C_RS_ decreases, leading to increased lung recoil and Slope_EF_, reducing T_Low_ (Figure 3).

The pressure–time and flow-time curves seen during APRV are shown in Figure 2. P_High_ denotes the pressure inflating the lung, with lung volume changing with respiratory system compliance (C_RS_) and the duration (T_High_) of the applied pressure (P_High_). These together define the continuous positive airway pressure (CPAP) phase of APRV (Figure 2).

##### Summary of basic science evidence

To our knowledge, only two translational animal studies have investigated the APRV mode, examining how variations in P_High_ affect lung injury and inflammation (40, 41). Both studies used a heterogeneous porcine lung injury model of surfactant deactivation, which included both normal lung areas and areas with surfactant deactivation simulating ARDS. This setup allowed for assessing the pathological impact of lung overdistension (OD) and recruitment-derecruitment (RD) in both normal and surfactant-depleted tissues.

Four groups of animals were studied, with mechanical breaths set in the APRV mode to induce lung overdistension (OD) (OD↑; P_High_ = 40 cmH_2_O) or not (OD↓; P_High_ = 28 cmH_2_O), and lung recruitment-derecruitment (RD) or not. The RD was caused (RD↑) or prevented (RD↓) by controlling the end-expiratory flow termination (F_EE_) point, by altering the expiratory time (T_Low_). The exact expiratory time was determined using the peak expiratory flow (F_PE_) equation: [F_PE_ x 25% (RD↑) or 75% (RD↓)] = F_EE_. A higher percentage (75%) resulted in a shorter expiratory time. The expiratory time (T_Low_) was set sufficiently brief to prevent alveolar collapse (RD↓), according to the equation,

$$F_{\mathrm{PE}}\times 75\%=F_{\mathrm{EE}}$$ (1)

which resulted in a very brief expiratory duration. The (T_Low_) was set sufficiently long to cause alveolar collapse (RD↑) using the equation (41).

$$F_{\mathrm{PE}}\times 25\%=F_{\mathrm{EE}}$$ (2)

This study did not evaluate efficacy or outcomes but aimed to identify the roles of the two main mechanisms of VILI, specifically OD and RD, on inflammation and pathophysiology. A P_High_ of 40 cmH_2_O, with or without RD, was found not to cause pulmonary edema in normal tissue and only induced edema in surfactant deactivation tissue when OD was combined with RD. A similar porcine ARDS model confirmed that P_High_ 40 cmH_2_O does not damage normal lung tissue and becomes problematic only when combined with RD (40).

A similar porcine ARDS model confirmed that a P_High_ of 40 cmH_2_O does not damage normal lung tissue and only becomes problematic when combined with alveolar instability (RD) (40). This supports the conclusion that overdistending normal tissue (i.e., the Baby Lung) is not the primary cause of VILI (41–45). This is because the Baby Lung in the ARDS patient is not normal tissue, similar to that in the animal studies described. Instead, it has heterogeneously distributed lesions of unstable, collapsed, or edema-filled alveoli that standard clinical imaging methods cannot detect. These lesions have been called “Hidden Microatelectasis” and function as stress multipliers that correlate with VILI. Maintaining P_High_ below 30 cmH_2_O during normal chest wall compliance (C_CW_) reduces lung tissue damage in areas of surfactant deactivation when a brief expiratory duration prevents alveolar collapse (RD). These studies thus demonstrate that normal lung tissue is highly resistant to damage from high airway pressures, provided cyclic collapse and reopening are avoided.

##### Recommendation

P_High_ should be sustained during the steep portion of the pressure-volume curve between functional residual capacity (FRC) and total lung capacity (TLC). Therefore, P_High_ should be set equal to plateau pressure (P_Plat_) when switching from volume control (VC) mode, to the peak inspiratory pressure (PIP) when switching from pressure control (PC) or dual targeted (DT) mode such as pressure regulated pressure control (PRVC) or mean airway pressure (Paw) plus 2–4 cmH_2_O when using the high frequency oscillatory ventilation (HFOV) mode. When using APRV as the initial mode, P_High_ recommendations are Mild ARDS (20–24 cmH_2_O), Moderate ARDS (25–29 cmH_2_O), and Severe ARDS (26–30 cmH_2_O), with the possibility of exceeding 30 cmH_2_O with low C_RS_.

**Figure 3:**
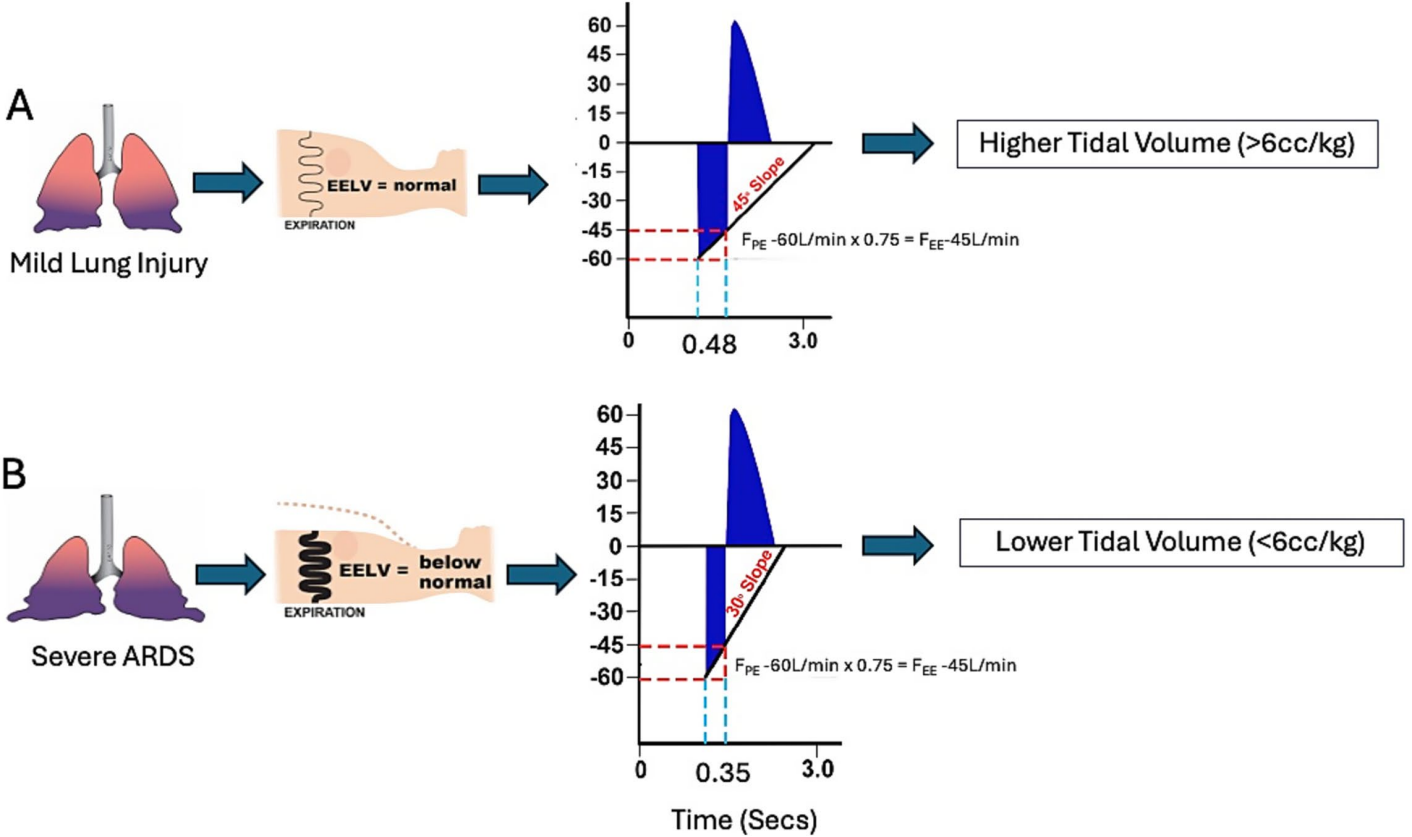
Utilizing the time-controlled adaptive ventilation (TCAV) method to set and adjust the time at low airway pressure (T_Low_) with the airway pressure release ventilation (APRV) mode personalizes: (1) expiratory duration (T_Low_), (2) tidal volume (V_T_), and (3) time-controlled PEEP (TC-PEEP). **(A)** With mild lung injury, the moderately reduced respiratory system compliance (C_RS_) is represented by a thin spring within the chest. This results in low lung recoil, and end-expiratory lung volume (EELV) is only moderately lowered. The expiratory gas flow-time curve (blue) observed on the ventilator monitor can be used to assess lung pathophysiology, as with spirometry. A shallow slope of the expiratory flow curve (Slope_EF_) indicates normal C_RS,_ while a steep slope indicates low C_RS_. With mild lung injury, the Slope_EF_ is shallow (45°). Equation 1 (F_PE_ x 75% = F_EE_) is used to calculate T_Low_. In this example, the F_PE_ = −60 L/min, so the clinician would set the F_EE_ at −45 L/min (60 L/min x 75% = 45 L/min). The duration from F_PE_ to F_EE_ is used to set T_Low_. In this case, T_Low_ = 0.48 s. The relatively long T_Low_ allows a larger volume of gas to be released (V_R_), resulting in a lower TC-PEEP and higher V_T_ (>6 cc/kg). **(B)** Severe ARDS has developed in the same patient, characterized by a very low C_RS_, high lung recoil (thick spring inside the chest), and loss of EELV (dotted chest wall outline). The same equation (Equation 1) is used to calculate T_Low_. However, the high lung recoil quickly expels air from the lung, resulting in a steep Slope_EF_ (30°). Rapid gas exhalation causes F_EE_ to be reached quickly, thereby decreasing T_Low_ (0.35 s). The brief T_Low_ results in a small V_R_, leading to a higher TC-PEEP and lower V_T_ (<6 cc/kg), precisely what is needed to protect a severely injured lung. To summarize, T_Low_, V_T_, and TC-PEEP are personalized and adjusted based on the patient’s lung pathophysiology, as indicated by lung spirometry (Slope_EF_) (2, 64).

Although tidal volume (V_T_) is not targeted, it is important to note that V_T_ is generally <6 mL/kg in a severely injured lung (Figure 3B), as described in detail under the T_Low_ setting section. With decreased C_RS_ / increased E_RS_, the V_T_ may be <4 mL/kg. In this instance, the P_High_ may be increased in 2 cmH_2_O increments to achieve a minimum V_T_ of 5–6 mL/kg.

Before transitioning to APRV, it is advisable to evaluate the patient’s intravascular status to assess preload dependency, for example, with the passive leg raise maneuver (46). Data show that patients with positive preload dependency respond effectively to a fluid bolus without developing pulmonary edema (47).

Because lung recruitment can occur rapidly, monitor lung volume on chest radiograph. Check the diaphragm’s shape, which should typically reach its maximum at the mid-clavicular line, crossing the fifth anterior rib. If the diaphragms start to flatten—that is, if the peak curvature moves medially from the mid-clavicular line—reduce P_High_ by 2–3 cmH₂O. Then, adjust T_Low_ as needed to keep the fraction of F_EE_ at 75% of the F_PE_ (Equation 1) (See T_Low_ adjustment below).

##### Justifications and implementation considerations

The above recommendations align with those for standard mechanical ventilation, namely maintaining P_High_ ≤ 30 cmH₂O with normal C_CW_. This approach is consistent with all ventilation mode guidelines and should not cause lung injury, as with other modes. Raising P_High_ above 30 cmH₂O may be necessary for patients with low C_CW_, such as those with significant central obesity, ascites, or abdominal distension (48).

A practical method is to gradually increase P_High_ in 2 cmH₂O steps while monitoring changes in C_RS_ and driving pressure (ΔP) (ΔP = V_T_/C_RS_). It is essential to recognize that positive changes in C_RS_ and P may take considerable time to manifest if lung reopening occurs gradually, suggesting prolonged opening time constants. In these cases, APRV gradually opens the lung (49).

##### Future research opportunities

Future research opportunities include further studies on using esophageal manometry to estimate pleural pressure (P_PL_). This method would allow P_High_ to be set above 30 cmH_2_O in patients with low C_CW,_ reducing concerns about overdistension by providing a direct measurement of transpulmonary pressure (P_TP_). The ARDSNet protocol was found to be less lung protective than the APRV mode set by the time-controlled adaptive ventilation (TCAV) method and guided by P_TP_ in a clinically relevant porcine model of sepsis and gut ischemia/reperfusion (50).

#### Question 2: what is the optimal method for setting P_Low_?

##### Background

During conventional mechanical ventilation, the positive end-expiratory pressure (PEEP) applied by the ventilator is the end-expiratory pressure during each breath cycle. Setting P_Low_ to 0 cmH₂O with APRV using T_Low_ calculated as a fraction of F_EE_ at 75% of F_PE_ (Equation 1) does not create a PEEP of 0 cmH₂O because expiration is too brief for the expiratory flow to cease and the lungs to depressurize fully. This results in a time-controlled PEEP (TC-PEEP) (Figure 2, red dashed line). In a translational large-animal model, a P_Low_ of 0 cmH₂O yielded a significant TC-PEEP, slightly more than half the P_High_ (50).

A brief expiratory duration, rather than a fixed pressure (PEEP), stabilizes alveoli because the expiratory time is shorter than the lung’s fastest time constant of closure. Therefore, setting P_Low_ above 0 cmH₂O is unnecessary and may be harmful, as it can increase PaCO₂ levels and hinder clearance of secretions by slowing expiratory gas flow (51). Unlike PEEP in traditional ventilation, T_Low_ dynamically adjusts EELV (14), providing a physiologically responsive method that better adapts to changes in lung mechanics than compliance-based adjustments, arbitrary scales, or oxygenation targets, which have limited predictive value for patient outcomes (52).

Setting a P_Low_ above 0 cmH_2_O creates expiratory resistance, reducing gas flow and causing two major issues:

1. Reduced expiratory flow limits CO₂ elimination per breath, and when combined with a brief T_Low_, can cause hypercapnia.
2. Expiratory flow when unimpeded by a set PEEP is a measure of C_RS_ and can be used to determine the correct T_Low_ (Equation 1). Adding resistance (setting a P_Low_) breaks this link, potentially leading to an incorrect T_Low_ setting. An excessively long T_Low_ has been shown to increase the risk of RACE-induced atelectrauma (41).

Thus, maintaining P_Low_ at 0 cmH_2_O is crucial for maintaining dynamic, patient-specific control of EELV and reducing the risk of ventilator-induced lung injury in APRV.

##### Summary of basic science evidence

**Figure 4:**
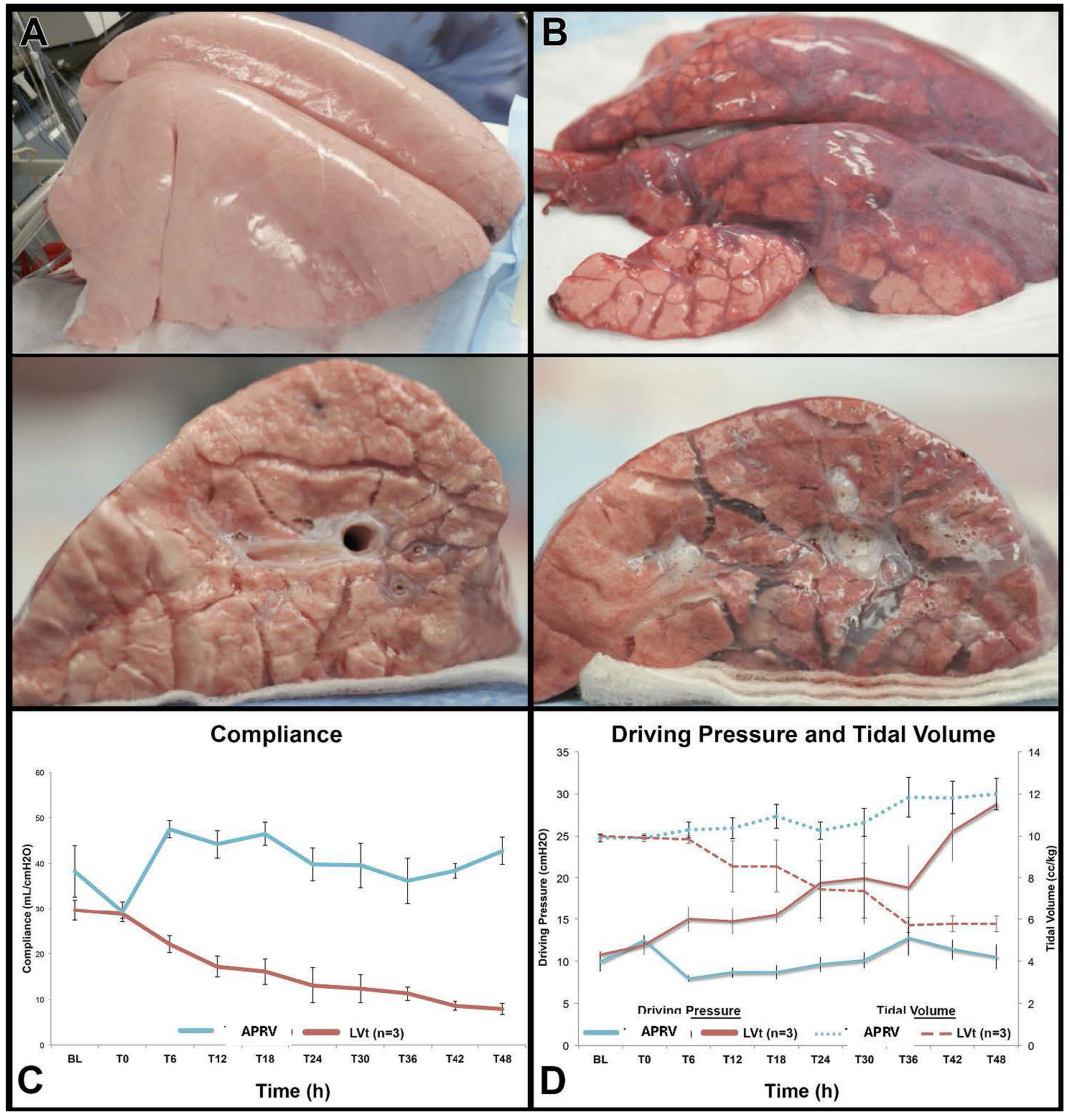
Gross lung photos at the end of a 48-h (T48) porcine sepsis plus ischemia/reperfusion acute respiratory distress syndrome (ARDS) model using the airway pressure release ventilation (APRV) mode set with the time-controlled adaptive ventilation (TCAV) method **(A)** or the ARDSnet low tidal volume (LV_T_) method **(B)**. Driving pressure (ΔP), tidal volume (V_T_), and respiratory system compliance (C_RS_) values over time for each mode **(C,D)** (28). **(A)** Forty-eight hours post-injury, the TCAV method displayed fully inflated lungs with no edema on the cut lung surface. **(B)** The ARDSNet method demonstrated significant atelectasis and edema on the cut lung surface. The V_T_ in the APRV group was 12 mL/kg at T48, but the ΔP was only 9 cmH_2_O because the C_RS_ remained elevated (ΔP=Vt/C_RS_).

In a translational porcine ARDS model comparing ARDSnet LV_T_ with APRV, it was observed that 48 h after injury (peritoneal sepsis plus gut ischemia/reperfusion), using P_Low_ = 0 cmH_2_O resulted in a TC-PEEP of 17.8 ± 2.0 cmH_2_O. The lung remained well-inflated, and ARDS was prevented (Figure 4) (50).

##### Recommendation

The P_Low_ should always be set to 0 cmH_2_O, but PEEP will be maintained because of the brief expiratory period (TC-PEEP) (Figure 2, red dashed line) (50). Setting P_Low_ to 0 cmH_2_O also improves CO_2_ clearance and mucus removal. A P_Low_ of 0 cmH_2_O enables expiratory flow to identify lung C_RS_, similar to spirometry, on a breath-by-breath basis for precise setting and adjustment of T_Low_.

##### Justifications and implementation considerations

**Figure 5:**
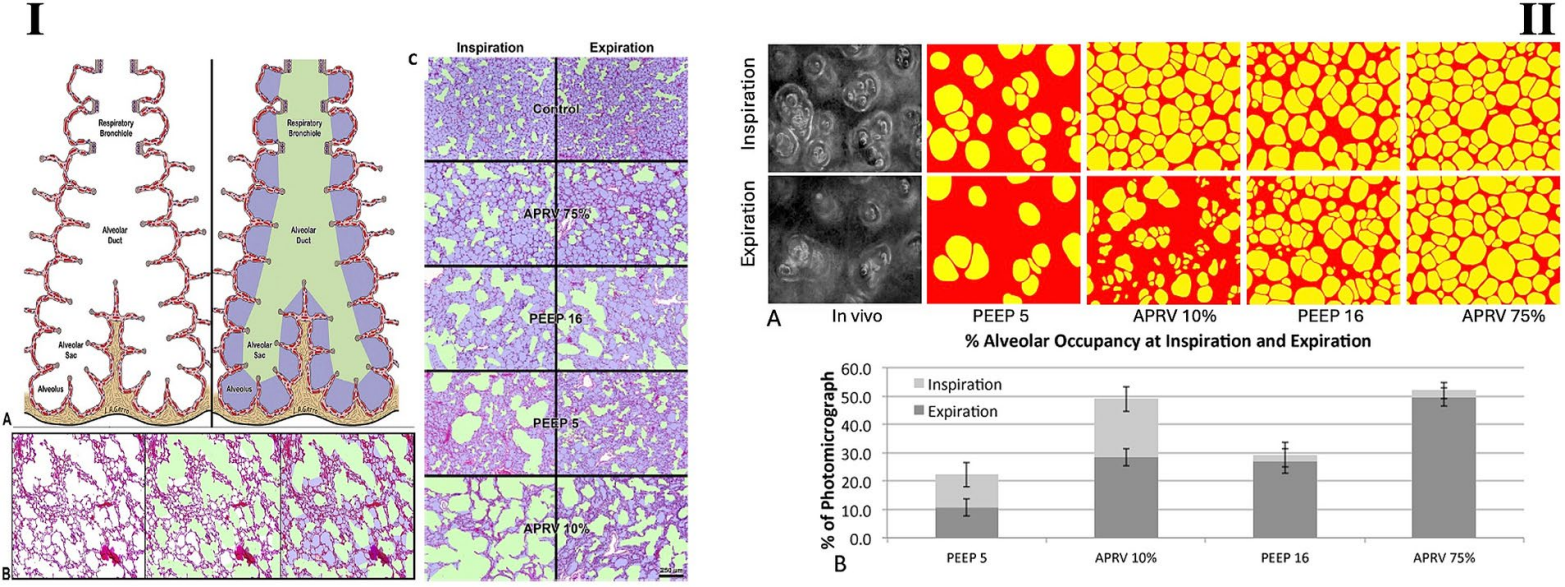
The impact of ventilatory modes and settings on alveolar and alveolar duct histology and real-time subpleural alveolar opening and collapse. Airway pressure release ventilation (APRV) mode set at different expiratory times (T_Low_) (APRV 10% – long T_Low_, APRV 75% – brief T_Low_, see Equations 1, 2) and the volume-controlled ventilation (VCV) mode with a low tidal volume (V_T_) of 6 mL/kg and two levels of positive end-expiratory pressure (PEEP) at PEEP 5 and PEEP 16 cmH_2_O. In APRV mode, the expiratory time (T_Low_) was determined by Equation 1: peak expiratory flow (F_PE_) multiplied by a cofactor (10% or 75%) to establish expiration termination: F_PE_ × 10% or 75% = F_EE_. Using a 10% cofactor prolongs the expiratory duration, whereas a 75% cofactor yields a very brief duration; the 75% cofactor is used in the time-controlled adaptive (TCAV) method. **I—**In this study, Tween was lavaged into the lungs to deactivate pulmonary surfactant, serving as an established model for ARDS, and one lung was fixed at inspiration. In contrast, the other was fixed at expiration. **(A,B)** A color-coding scheme is used to distinguish between alveolar ducts (green), alveoli (blue), and tissue (red) for computer image analysis. **(C)** The impact of the two ventilator modes and the four ventilator settings on duct and alveolar size at inspiration and expiration is compared to an uninjured (no Tween) control group. Gas distribution in the alveolar tissue is highest in the control group, with 68% during inspiration (I) and 50% during expiration (E), as measured by computer image analysis of the histologic slide. The alveolar duct volume was slightly under 19%-I and 16%-E; the remaining area was tissue. Tween injury increased duct volume in all groups, whereas alveolar volume decreased. APRV 75% yielded the gas distribution closest to control (ducts at 30%-I and 25%-E, and alveoli at 49%-I and 48%-E). APRV 10% was the least effective at alveolar ventilation, with duct occupancy at 50%-I and 35%-E and alveoli at 27%-I and 29%-E. The disparity between inspiratory and expiratory occupancies reflects dynamic strain (atelectrauma), which is significantly elevated in the alveolar ducts of the APRV 10% group. It was also found that the micro-strain (*ε*-strain) was 0.14 in the control group, increased to 0.20 in the APRV 75% group, and rose significantly to 0.39 in the APRV 10% group. The ε-strain for PEEP 5 was 0.34, and PEEP 16 was 0.23. This indicates that APRV 75% with a very brief expiratory time (T_Low_) is most effective at normalizing gas distribution and reducing ε-strain in an acutely injured lung. The very different impact of the APRV mode with different expiratory durations (APRV10% – long T_Low_ and APRV 75% -very brief T_Low_) on lung histopathology and dynamic alveolar mechanics demonstrates that proper setting of the APRV mode is crucial (13). **II**—**(A)** Photomicrographs of subpleural alveoli were recorded using *in vivo* microscopy during both inspiration and expiration. Black-and-white photographs of subpleural alveoli were captured with the *in vivo* microscope at *inspiration* and *expiration*. Individual alveoli are circled and color-coded in yellow, while collapsed alveoli are marked in red, using computer image analysis. The effects of APRV with two expiratory times (10 and 75%) and VCV with a low V_T_ (6 mL/kg) and two positive end-expiratory pressure (PEEP) levels (5 and 16 cmH_2_O) on alveolar recruitment and collapse were measured. For APRV, the expiratory time was determined using the equation F_PE_ x 0.10 or 0.75 = F_EE_. Two additional groups using 0.50 and 0.25 cofactors to determine the expiratory time with APRV were also investigated (data not shown). The difference in the number of alveoli (yellow) between inspiration and expiration directly reflects repetitive alveolar collapse and expansion (RACE), which is the mechanism of atelectrauma. **(B)** The numerical difference between the volume of open alveoli in the microscopic field during inspiration and expiration **(A)** was used to calculate RACE. The difference in the area of the photomicrograph occupied by alveoli during inspiration (light gray bar) and expiration (dark gray bars) indicates the level of alveolar instability (RACE). VCV with PEEP 5 did not recruit alveoli during inspiration nor prevent collapse during expiration, resulting in a high RACE level. PEEP 16 moderately increased recruitment while minimizing RACE. Both APRV groups had prolonged inspiratory time (T_High_), resulting in significant alveolar recruitment. Increasing expiratory time (T_Low_) (APRV 10%) caused substantial alveolar collapse at expiration, resulting in more RACE than PEEP 5. APRV 75% was the only strategy that opened and stabilized the alveoli, thereby preventing RACE and reducing stress multiplication. % of Photomicrograph, the percent of the photomicrograph covered by inflated alveoli (color coded yellow) (12).

Direct observation of subpleural alveoli shows that a P_Low_ of 0 cmH_2_O does not cause RACE when T_Low_ is determined using a fraction of F_EE_ at 75% of F_PE_ (Figure 5, II). Although P_Low_ is set to 0 cmH_2_O, the lung does not have enough time to depressurize, maintaining a TC-PEEP (Figure 2, red dashed line) (50). Research indicates that alveoli are better stabilized with a brief expiratory time that generates TC-PEEP compared to using a non-zero PEEP level with a longer expiratory duration that results in the same end-expiratory lung volume (Supplementary File 3, Figure S9) (5). In the acutely injured lung, gas distribution within the alveoli and ducts closely resembles normal conditions, with a P_Low_ of 0 cmH_2_O and a T_Low_ set using Equation 1 (Figure 5, I). This results in dynamic alveolar homogeneity similar to that of a normal lung (11), thereby facilitating CO_2_ and mucus removal (5).

##### Future research opportunities

Investigate alternative methods to determine T_Low_ beyond using expiratory flow magnitude alone. These methods might allow P_Low_ to exceed 0 cmH_2_O without compromising the primary benefits of APRV, provided that CO_2_ retention and mucus clearance are not adversely affected.

#### Question 3: what is the optimal method for setting T_High_?

##### Background

Alveolar RD is a dynamic process influenced by both time and pressure. When increased pressure is applied to a derecruited lung, alveolar units gradually open even if the pressure remains constant (53). In specific lung injuries characterized by slow recruitment kinetics, this process may persist throughout the entire T_High_ and across multiple consecutive breaths. Although the volume of lung recruited during a single T_High_ block may be small, the overall effect across multiple breaths can be significant, provided derecruitment is avoided during each T_Low_. Therefore, using a prolonged T_High_ while still achieving sufficient expired minute volume (MVe), combined with a brief T_Low_ set according to Equation 1, can effectively gradually ‘ratchet’ open atelectatic lung regions that do not respond to conventional PEEP strategies (49). However, increasing T_High_ lowers the respiratory rate (RR) and, consequently, reduces MVe. Although an intuitive misconception to increase MVe is to increase the T_Low_ thereby increasing V_T_. However, this increases the risk of RACE-induced atelectrauma and should be avoided. This is explained below in the recommendations for the T_Low_ setting. The more appropriate method is to decrease T_High_ transiently to increase RR and MVe. Although this might slow lung recruitment, it will prevent RACE-induced VILI. As the lung is progressively ‘ratcheted’ open, gas exchange improves, and the T_High_ can be gradually increased.

The appropriate T_High_ should be customized according to the severity of lung dysfunction and adjusted based on the patient’s ventilatory efficiency to achieve normocapnia as lung function improves. Initially, T_Hgh_ is set to ensure adequate bulk (convective) ventilation and MVe, particularly in patients with severe lung injury who require a higher RR. The T_High_ is the rate controller in APRV and is similar to increasing the RR in conventional ventilation.

For example, in patients with established ARDS and hypercapnia transitioning to APRV, an initial T_High_ of 1–2 s is typically used, yielding an RR of approximately 24–35 breaths per minute. In this setup, a brief T_High_ is combined with a brief T_Low_ to keep the fraction of F_EE_ at 75% of F_PE_ (Equation 1), thereby maintaining functional EELV and normalizing lung E_RS_. This creates a respiratory cycle characterized primarily by inspiration (Figure 2, CPAP Phase).

**Figure 6:**
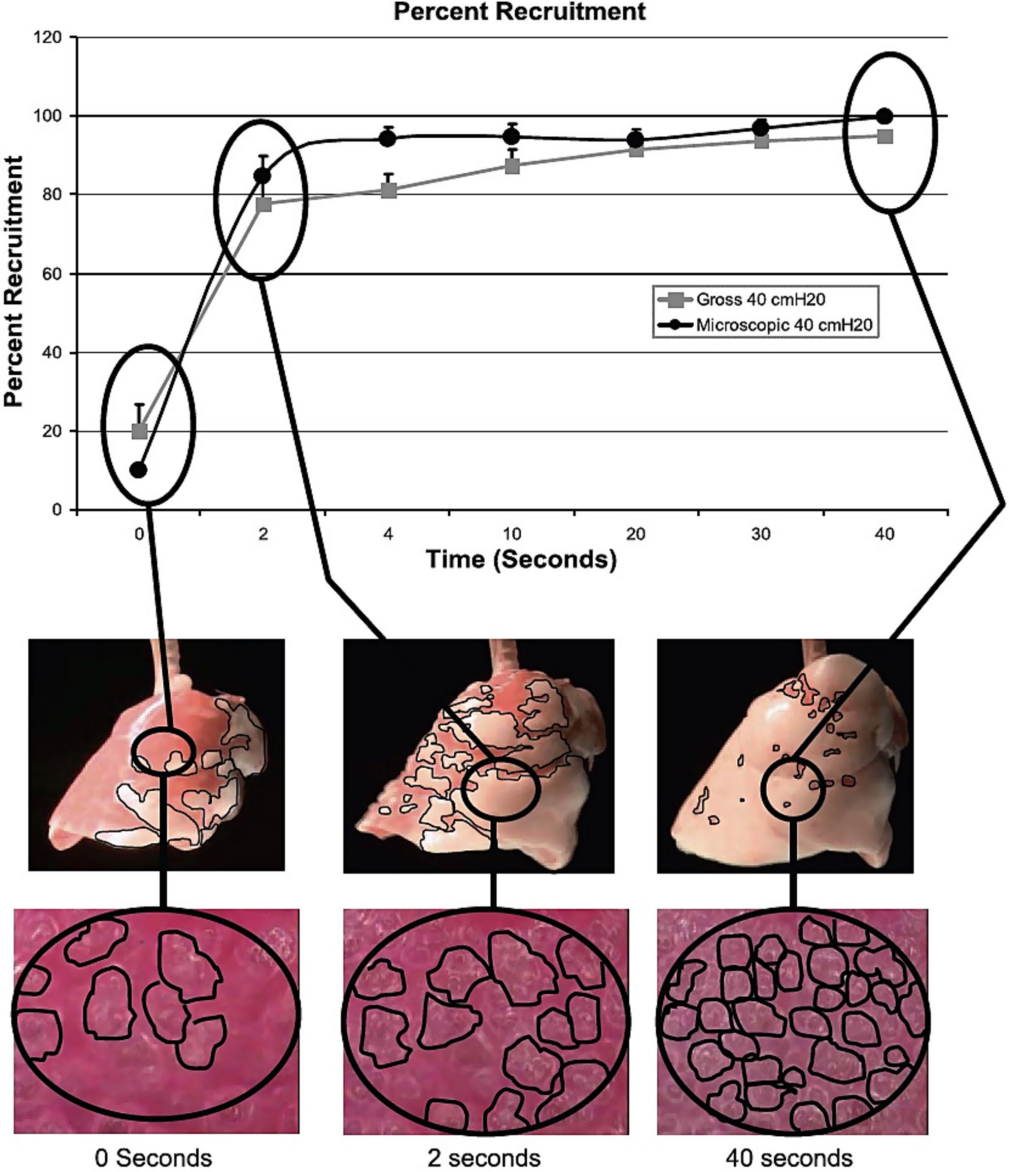
Time- and pressure-dependent alveolar recruitment. In a rat model of Tween lavage-induced ARDS, subpleural alveolar and overall lung recruitment were measured at three static pressures (20, 30, and 40 cmH_2_O) held for 40 s. The percentage of recruited subpleural alveoli in the microscopic field (black circles) and the gross lung recruitment on the lung surface, indicated by red atelectatic tissue (black lines) turning pink, were analyzed using computer image analysis. A time lag of approximately 2 s, reflecting viscoelastic response, was observed between pressure application and the onset of recruitment. This was followed by rapid recruitment, then by gradual, continuous recruitment throughout the 40-s inflation period, indicating viscoelastic behavior. The airway pressure level also influenced recruitment (data not shown). These viscoelastic recruitment patterns were accurately predicted by a mathematical model that incorporated the effects of airway pressure and time. This study demonstrated that lung tissue can be recruited by extending the inspiratory time without additional mechanical power or energy cost.

The T_High_ controls convective and diffusive ventilation and also regulates end-inspiratory lung volume by recruiting additional alveoli with each breath, due to the time-dependent nature of recruitment (Figure 6) (35). Reopening the lung increases the surface area for gas exchange, thereby improving CO_2_ clearance through diffusive ventilation. Reopening also reduces local stress concentrators in the tissue (Supplementary File 3, Figure S2), thereby eliminating a significant mechanism of VILI (11–13).

In contrast, for stable patients recovering from ARDS or receiving APRV as a preventive or lung-protective strategy, a longer initial T_High_ (e.g., 4–6 s) may be used. This approach reduces RR and MVe; however, because most of the lung remains open, there is sufficient diffusion surface area to maintain normal PaCO_2_.

Although permissive hypercapnia may be acceptable in selected cases at the clinician’s discretion, inducing hypercapnia is not a goal of APRV. Reports of hypercapnia in the literature are most commonly attributable to an excessively prolonged T_High,_ particularly when ventilator settings are not appropriately adjusted to the degree of lung dysfunction during the early transition phase. Accordingly, initial APRV parameters should not be selected to target hypercapnia or to employ a prolonged T_High_ that reduces MVe relative to prior ventilator settings. Instead, the primary objective is to maintain normocapnia with adequate MVe, even when the initial T_High_ is brief—though still approximately twice the duration of conventional inspiratory times. Lung reopening is initiated within this time frame.

As a patient’s ventilatory efficiency improves, T_High_ can be gradually increased. This adjustment is usually guided by trends toward normocapnic or even hypocapnic, which indicate effective CO₂ removal. Increasing T_High_ shifts ventilation from mainly convective, RR-driven mechanisms to a combination of convective and diffusive processes. This transition allows for significant reductions in both RR and MVe without compromising overall ventilation.

##### Summary of basic science evidence

The recruitment of alveolar lung tissue at three different airway pressures over time was measured using a rat ARDS model (Figure 6) (35). Gross lung recruitment was assessed by imaging the lung surface and calculating the recruitment rate (the proportion of red tissue that turned pink). Alveolar recruitment was evaluated using *in vivo* microscopy, and the proportion of the microscopic field occupied by alveoli was quantified by computer image analysis. Rapid recruitment occurred within the first 2 s, followed by gradual recruitment over the next 40 s. The results were accurately predicted by a mathematical model that incorporated both time and pressure. Extending the T_High_ will continuously recruit lung tissue without additional mechanical power or ΔP costs (35).

##### Recommendation

**Figure 7:**
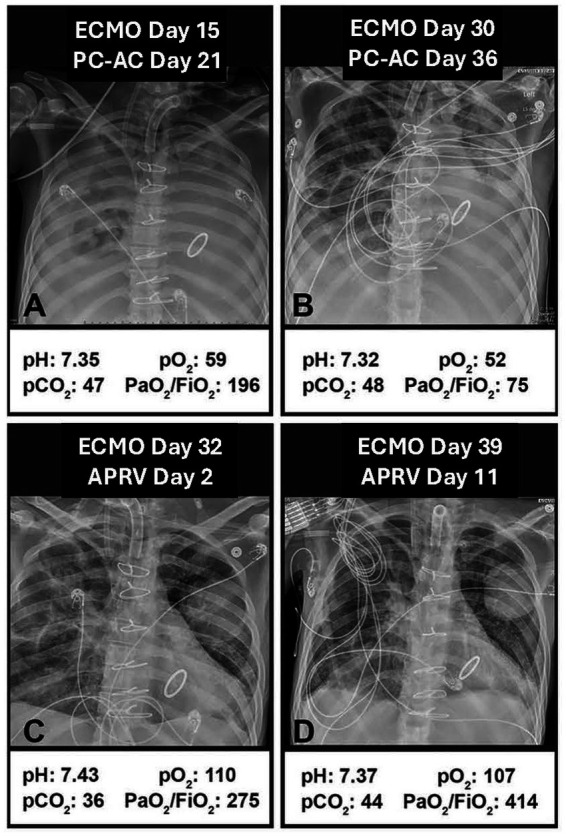
A 35-year-old man with catastrophic antiphospholipid syndrome and respiratory failure. **(A)** ARDSnet low tidal volume ventilation with pressure–control assist–control (PC-AC) mode, day 21, with extracorporeal membrane oxygenation (ECMO), day 15, and **(B)** day 36 on PC-AC with ECMO,. Transitioned to APRV on day 36. **(C)** 2 days on APRV, the lungs opened (clearer chest X-rays), and arterial blood gases (ABGs) improved. **(D)** After 11 days on APRV, the chest X-ray was clear, the ABGs normalized, and the patient was removed from ECMO and mechanical ventilation (54). This case demonstrates the gradual ‘ratcheting’ open of the lung tissue over several days with the use of the APRV (49).

A T_High_ of 4–6 s is typical for patients with routine post-operative atelectasis or normal lungs, but it may be too long when used as a rescue strategy for patients in respiratory failure. In these cases, a brief T_High_ of 2–3 s may be necessary to increase MVe until the lung has recruited enough to exchange an adequate volume of CO_2_. This may not occur for 12–24 h, or even 24–48 h in lungs with severe ARDS (Figure 7) (54).

Note that if V_T_ falls below 3 mL/kg of ideal body weight after switching from conventional ventilation to APRV, it may be necessary to lower T_High_ and raise P_High_ to maintain or exceed the pre-transition MVe. When switching from another ventilation mode, set T_High_ to match the previous RR using the formula: 60/RR – T_Low_ = T_High_.

For example, with an RR of 26 during conventional ventilation and a T_Low_ of 0.5 s, the T_High_ is 60/26 = 2.3 s minus 0.5 s (T_Low_), yielding 1.8 s. When using APRV as the primary mode, set T_High_ to 2–4 s for Mild ARDS, 2–3 s for Moderate ARDS, and 1–3 s for Severe ARDS. After obtaining blood gas results, T_High_ can be adjusted up or down to maintain a normal PaCO_2_.

##### Justifications and implementation considerations

When switching from conventional ventilation to APRV for a patient with severe ARDS, the T_High_ should be brief to match the required conventional ventilation RR and MVe, ensuring that ventilation remains sufficient to maintain normocarbia. As the lung gradually recruits, the gas-exchange surface area increases, allowing T_High_ to be extended to promote lung recruitment while maintaining normocarbia.

##### Future research opportunities

Currently, the optimal combination of T_High_ and P_High_ for optimizing V_T_ and MVe is determined empirically. Future work should focus on developing a computational approach to identify the optimal combination for each patient’s lung condition, using techniques such as fuzzy logic.

#### Question 4: what is the optimal method for setting T_Low_?

##### Background

Personalizing T_Low_ based on lung pathophysiology is essential for achieving lung-protective effects with APRV. T_Low_ is adjusted using C_RS_ (Equation 1), which effectively prevents RACE. RACE causes tissue injury by over-distending open alveoli near collapsed lung regions and avoids harmful stresses on opposing epithelial walls during inflation (Supplementary File 3, Figure S3) (55). Recently, we demonstrated in a pig model of surfactant deficiency that the energy dissipated in parenchymal tissues due to RACE correlates with outcomes (Supplementary File 3, Figure S5) (56), and that alveolar collapse is more effectively prevented using a brief expiratory time combined with TC-PEEP than with the same set-PEEP and a longer expiratory duration (Supplementary File 3, Figure S9) (5). When T_Low_ is set using Equation 1, it prevents progressive EELV loss and steers the lung away from the VILI Vortex (37).

The T_Low_ influences V_T_, which is affected by changes in C_RS_. A low C_RS_, common in *Severe ARDS*, results in a brief T_Low_ when using the TCAV method. This reduces the amount of gas that escapes during exhalation, often resulting in a V_T_ below 6 mL/kg. As the lung heals and C_RS_ increases, both T_Low_ and V_T_ also increase (Figure 3). Therefore, when correctly set, APRV will not deliver dangerously large V_T_ into a severely injured lung because it adjusts with changes in C_RS_, keeping ΔP (V_T_/C_RS_) within safe limits (Figure 3).

##### Summary of basic science evidence

The T_Low_ controls V_T_ and EELV, and therefore the level of TC-PEEP (Figure 3). After expiration begins, expiratory flow quickly reaches its peak value, F_PE_, then decreases progressively. The rate of decrease depends on the mechanical properties of the lungs, which change with acute lung injury, and remains approximately constant during the brief period defined by T_Low_. This means that T_Low_ can be calculated from the expiratory flow curve, assuming it is a straight line, by identifying the point at which flow reaches 75% of F_PE_ (Equation 1).

During the Release Phase (P_Low_/T_Low_) (Figure 2), gas flow decreases at an approximately steady rate, reflecting the mechanical properties of the respiratory system. Since the duration of the Release Phase is calculated as F_PE_ × 75% = F_EE_ (Equation 1), the T_Low_ is customized to the patient’s respiratory mechanics’ timeline (Figure 3). This strategy has been validated clinically (Figure 7) and experimentally (Figure 5) to optimize alveolar stability, meaning it prevents alveolar collapse during exhalation and reinflation during inhalation (atelectrauma) (11–13, 40, 41, 57).

**Figure 8:**
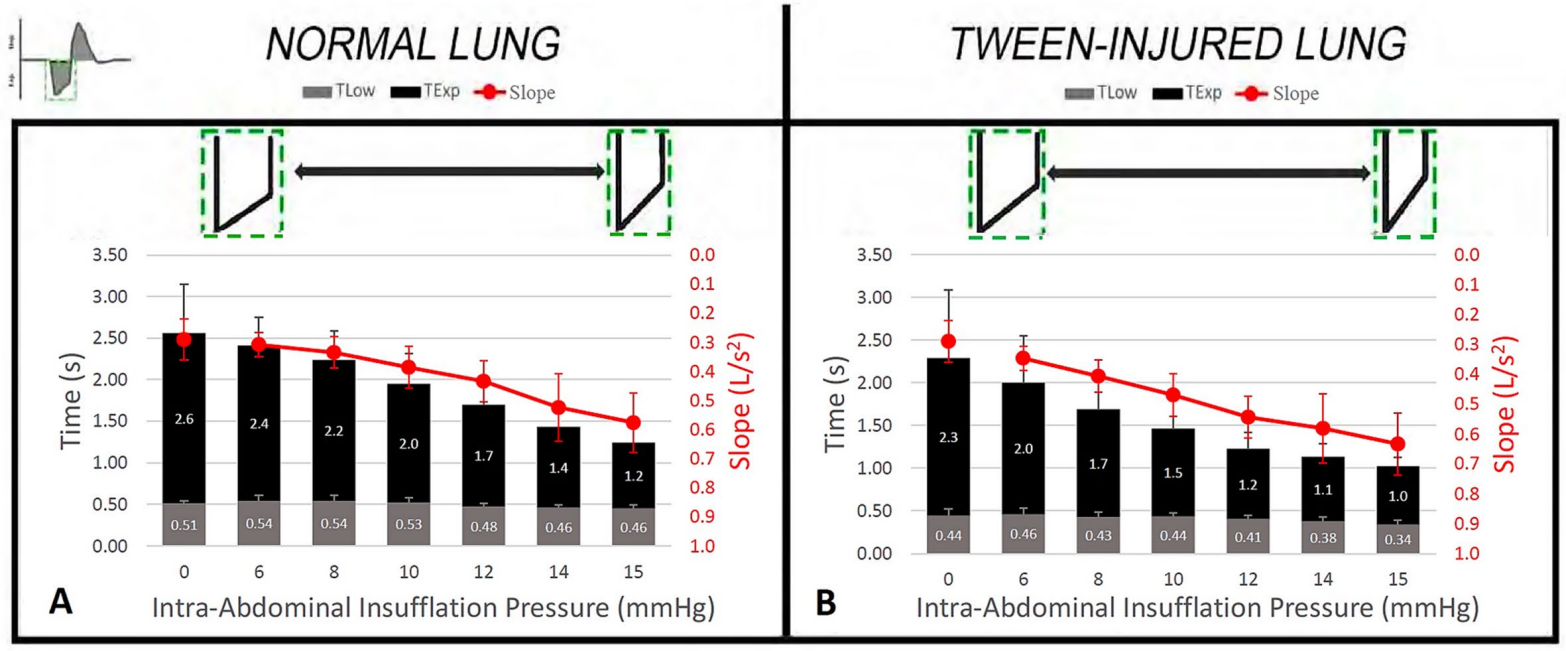
The relationship between changes in respiratory system compliance (CRS) and expiratory time (T_Low_, grey) during airway pressure release ventilation (APRV) is shown. This coupling occurs when T_Low_ is set as a fraction (75%) of the peak expiratory flow (Fig. 2); the lower the CRS, the briefer the T_Low_. Increasing intra-abdominal pressure (IAP) causes a gradual decrease in CRS. The slope of the expiratory flow curve (Slope_EF_, red) is a breath-by-breath measure of CRS using a method similar to spirometry. The expiratory time needed to reach zero expiratory flow and pressure (T_Exp_, black) is compared with the T_Low_ setting (T_Low_, grey). Note that the T_Low_ is set to terminate before the expiratory flow and pressure reaches zero. **(A)** Normal Lung: As CRS decreases with increased IAP, the Slope_EF_ (red) also decreases. This leads to a progressive reduction in T_Low_ from 0.51 seconds at an IAP of 0 mmHg to 0.46 seconds at an IAP of 15 mmHg. **(B)** Tween-Injured Lung: The same relationship between changes in CRS (i.e.,Slope_EF_) and the T_Low_ is observed in the acutely injured lung with surfactant deactivation by Tween lavage. As IAP is increased to reduce CRS, T_Low_ decreases from 0.44 seconds at an IAP of 0 mmHg to 0.34 seconds at an IAP of 15 mmHg (37). Note that T_Low_ is consistently lower in the acutely injured lung at each level of IAP.

The T_Low_ is the key parameter that personalizes APRV settings based on lung pathophysiology and regulates V_T_, TC-PEEP, and EELV (Figure 8). The Slope_EF_ is a breath-by-breath measure of C_RS_. A decrease in C_RS_ increases expiratory flow rate, resulting in a steeper slope and a more rapid attainment of the calculated F_EE_ (Equation 1), thereby shortening T_Low_ (Figure 3). Figure 8 illustrates the relationship between C_RS_, T_Low_, and Slope_EF_.

Substantial experimental evidence supports the idea that effectively using expiratory time stabilizes alveoli (Figure 5) (11–13), preventing RACE-induced energy dissipation (Supplementary File 3, Figure S5, S6) and tissue damage (Figure 4) (5). In a clinically relevant 48-h porcine model of peritoneal sepsis and gut ischemia/reperfusion, personalized T_Low_, along with the other recommended parameters, offered significant lung protection (Figure 4) (28, 50, 58). We showed that personalized T_Low_ prevents EELV loss, maintains normal C_RS_ and ΔP even with a V_T_ of 12 mL/kg (28). When T_Low_ is set using Equation 1, together with the three other recommended settings, it progressively increases C_RS_ while maintaining ΔP within the safe range (ΔP = V_T_/C_RS_) (Figure 7).

Histologic analysis of alveoli and alveolar ducts during inspiration and expiration (Figure 5, I) (13), along with direct observation of subpleural alveoli using *in vivo* microscopy (11, 12), highlights the critical role of personalized T_Low_ in lung stability (alveoli and ducts) (Figure 5, II). Using computational models, we confirmed the influence of time on alveolar recruitment and stabilization, as shown in animal studies (29–33), along with computational validation of the biological data (Supplementary File 6) (35, 36).

The relationship between T_Low_, C_RS_, and Slope_EF_ was examined in a recent study (Figure 8) (14). The study used normal pigs and a Tween-induced surfactant deactivation ARDS model. The C_CW_ was decreased by peritoneal CO_2_ insufflation to elevate abdominal pressures (0, 6, 8, 10, 12, 14, 15 mmHg). The T_Low_, Slope_EF_ (liters/s^2^), EELV, and the time to reach complete expiration to functional residual capacity (T_Exp_) were measured at each level of C_CW_.

As C_RS_ decreased with increasing intra-abdominal pressure, T_Low_, T_Exp_, and Slope_EF_ all progressively declined in both normal and Tween-injured (ARDS model) lungs (Figure 8). This study demonstrates the interconnected relationship between T_Low_, T_Exp_, and Slope_EF_, and how T_Low_ is coupled with C_RS_, which Slope_EF_ measures on a breath-by-breath basis. Changes in the Slope_EF_ can also be used to detect worsening lung injury and estimate EELV (14).

The clinician adjusts T_Low_ until the calculated F_EE_ is achieved (Equation 1). For example, if F_PE_ is 60 L/min, then F_EE_ would be 60 × 75% = 45 L/min, so the clinician adjusts T_Low_ so that the next expiration begins when F_EE_ reaches 45 L/min (Figure 3). A patient with mild lung injury shows a slight decrease in C_RS_ along with a small increase in lung recoil and normal EELV (Figure 3A). Because gas exits the lung slowly, the Slope_EF_ is gradual, and terminating expiration at F_EE_ = 45 L/min, as calculated by Equation 1, results in a T_Low_ of 0.48 s. Severe ARDS significantly reduces C_RS_, increases lung recoil, and decreases EELV (Figure 3B). High lung recoil quickly expels gas, creating a steep Slope_EF_ so that the same F_EE_ (−45 L/min) results in a T_Low_ of only 0.38 s. As mentioned earlier, T_Low_ is guided by changes in C_RS_.

Setting T_Low_ using Equation 1 typically yields a V_T_ < 6 mL/kg in a severely injured lung (Figure 3B). This occurs because V_T_ and C_RS_ are coupled, and this relationship varies with Slope_EF_ angle (Low C_RS_ = low V_T_ and vice versa). In cases of mild lung injury with relatively normal C_RS_ and EELV, a shallower Slope_EF_ results in an increase in T_Low_. This leads to greater Release Gas Volume (V_R_) and lung pressure, resulting in a relatively high V_T_ (>6 mL/kg) and a lower TC-PEEP (Figure 3A).

In contrast, severe ARDS, characterized by low C_RS_ and EELV, increases lung recoil, leading to a steeper Slope_EF_ and a significant reduction in T_Low_. This decreased V_R_ requires a similar small V_T_ during inflation (<6 mL/kg) while increasing TC-PEEP (Figure 3B). At the end of a 48-h porcine model of peritoneal sepsis and gut ischemia/reperfusion (PS + I/R)-induced ARDS, the APRV group, with settings similar to those recommended in this consensus conference, had a V_T_ of 12 mL/kg and C_RS_ of 42 mL/cmH_2_O, compared to the LV_T_ group with a V_T_ of 5.8 mL/kg and C_RS_ of 9 mL/cmH_2_O. Even with a V_T_ of 12 mL/kg—believed to cause VILI—the APRV group maintained a safe ΔP because of the high C_RS_ (ΔP = V_T_/C_RS_) (Figure 4). Therefore, it is not just the size of the delivered V_T_ that causes injury, but also the volume and lung pathophysiology into which it is introduced that determine whether a mechanical breath is harmful or protective.

##### Recommendation

When switching from another ventilation mode, initially set T_Low_ to 0.3–0.6 s. When starting with ARPV for Mild ARDS, use 0.4–0.6 s; for Moderate ARDS, use 0.3–0.5 s; and for Severe ARDS, use 0.2–0.3 s.

**Figure 9:**
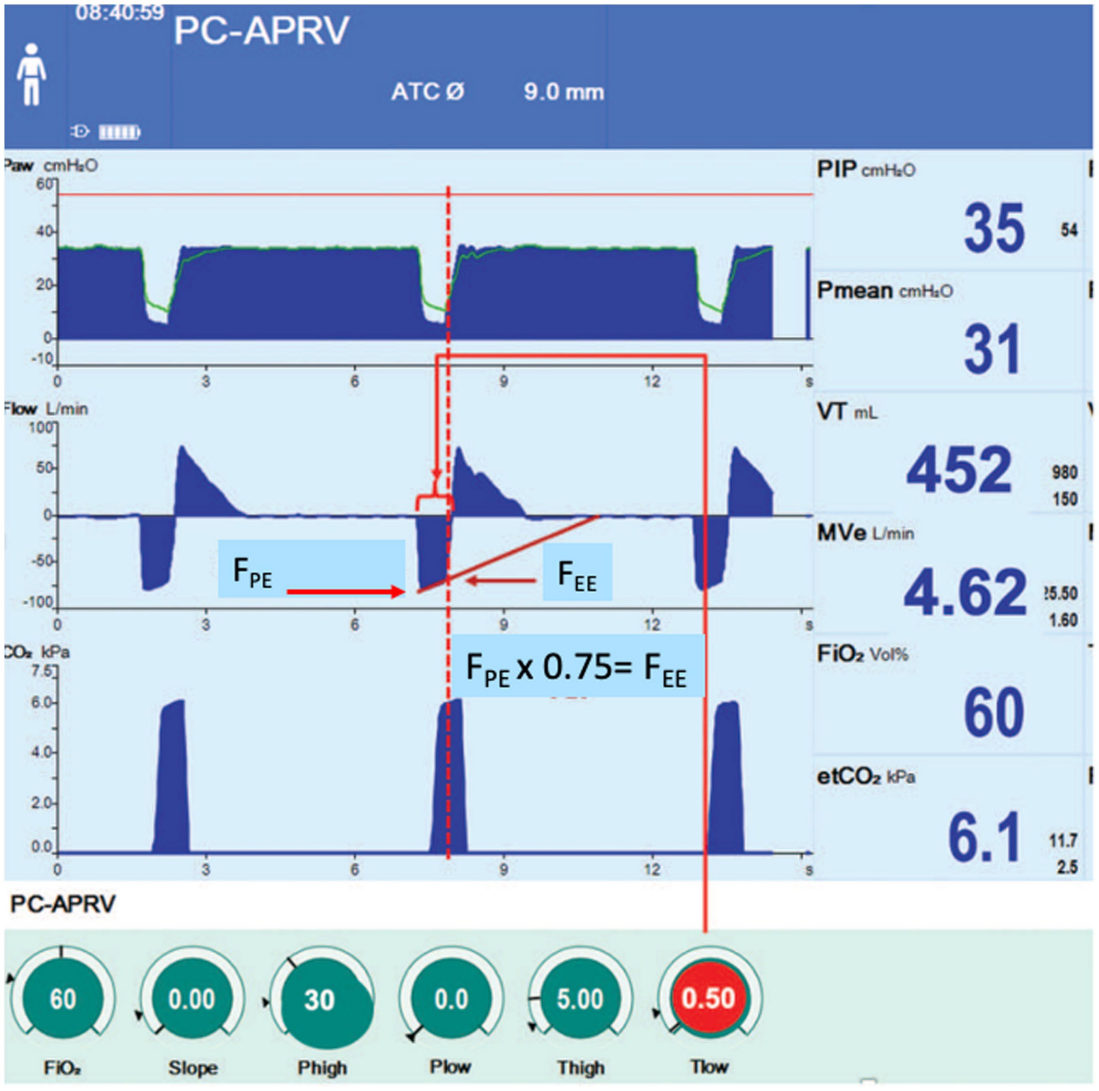
Setting expiratory time (T_Low_) as shown on a ventilator monitor. The peak expiratory flow (F_PE_) is identified and, in this example, measures ~75 L/min. Equation 1 is used to calculate the end expiratory flow (F_EE_) (F_PE_ 75 L/min x 75% = F_EE_ 56 L/min), at which point expiratory flow will be terminated and the lung reinflated. The T_Low_ is adjusted (red controller and line) to terminate at 56 L/min, which occurs at 0.5 s. The T_Low_ dial is not adjusted to a specific time but rather to end expiratory flow and reinflate the lung at the calculated value (56 L/min). If the T_Low_ set initially does not reach the calculated F_EE_ target, it will be adjusted upward or downward until it does (65).

Once the patient stabilizes, T_Low_ is customized based on the Slope_EF_ of the expiratory flow waveform (Equation 1). Figure 9 shows the clinician’s observation when T_Low_ is calculated and set according to Equation 1. In this example, F_PE_ is approximately 75 L/min. Therefore, using Equation 1 (F_PE_ 75 L/min x 75% = F_EE_ 56 L/min), the clinician should adjust T_Low_ until the calculated F_EE_ (56 L/min) is reached (Figure 9). Thus, the clinician does not set T_Low_ to a specific time but instead to reach the calculated F_EE_. The time taken for the lung to empty from F_PE_ to F_EE_ represents the desired T_Low_ (Figure 9, red bracket). Ideally, T_Low_ is fine-tuned in 0.01-s steps for precise control. If V_T_ is decreased, thereby decreasing MVe, the T_Low_ should not be increased beyond 75% to achieve MVe to increase V_T_ or improve CO_2_ removal as this has been shown to increase the risk of RACE-induced atelectrauma (41).

##### Justifications and implementation considerations

Histological analysis of a rat ARDS model, with lungs fixed at inspiration and expiration, showed that the T_Low_ set, using Equation 1, effectively distributes gas throughout the alveoli and ducts, closely resembling normal lung function (Figure 5, I). In contrast, T_Low_ determined using the equation F_PE_ x 10% = F_EE_ (APRV 10%) extends T_Low_, leading to overdistension of the alveolar ducts (Figure 5, I). Direct *in vivo* visualization of subpleural alveoli revealed that the T_Low_ set using Equation 1 was the most effective in recruiting and stabilizing alveoli. The APRV 10% group successfully recruited alveoli during inspiration with the extended T_High_ but failed to prevent collapse during exhalation, resulting in RACE (Figure 5, II). Using the same methodology, it was shown that T_Low_ set with Equation 1 was most effective in achieving uniform alveolar behavior compared to T_Low_ set with the following cofactors: F_PE_ x 10, 25, 50% = F_EE_ (11).

**Figure 10:**
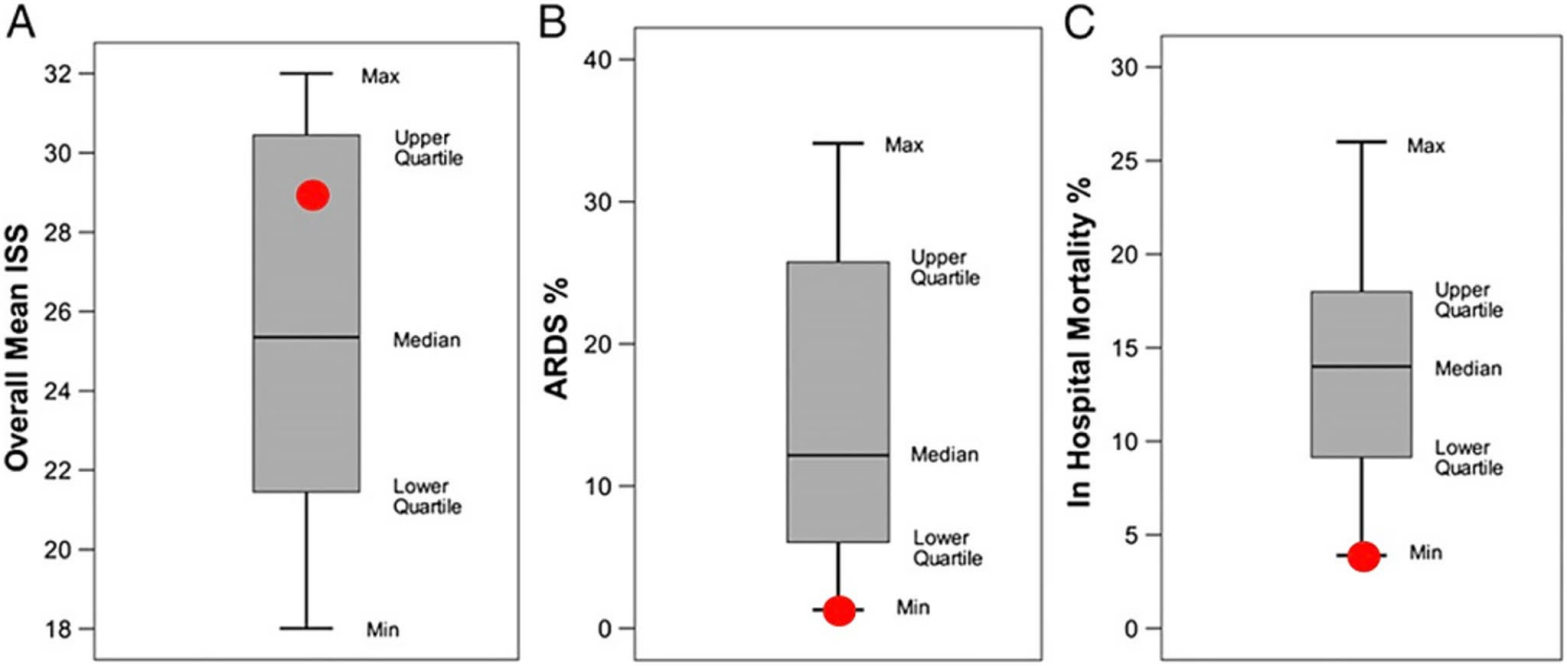
A systematic review was conducted comparing conventional ventilation (bar and whisker) in 16 intensive care units (ICUs) with airway pressure release ventilation (APRV) set and adjusted using the time-controlled adaptive ventilation (TCAV) method at the R Adams Cowley Shock Trauma Center (red dots). The TCAV patients were in the upper quartile for the Injury Severity Score (ISS), demonstrating severe injury. The percentage of patients on TCAV who developed ARDS (ARDS%) during their hospital stay was below the lower quartile (red dot). The Lung Injury Prediction Score (LIPS) in the TCAV group was 8.7, indicating that the ARDS incidence (ARDS%) should be 35%. However, the incidence of ARDS compared with the 16 other ICUs was 1.3% vs. 14.0%, and in-hospital ARDS mortality was 3.9% vs. 14.1%, using the TCAV method. This demonstrates that TCAV, when applied early, can reduce the incidence and mortality associated with ARDS.

Three studies using a clinically relevant 48-h porcine PS + I/R ARDS model showed that setting T_Low_ based on Equation 1, along with three other consensus conference-recommended settings, provided significant lung protection (Figure 4) (28, 50, 58). Additionally, case studies (Figure 7) (59, 60) and a statistical comparison involving ICU patients (Figure 10) (57) suggest that the T_Low_ set, as defined by Equation 1, in combination with the three other recommended settings, provides significant lung protection.

##### Future research opportunities

Extensive scientific and clinical evidence support using the recommended Equation 1 to set T_Low_ when employing the APRV mode (Supplementary Files 3–7). Of the four APRV parameters, the recommendations for setting T_Low_ and P_Low_ have the most published scientific backing (Supplementary File 2). Future work to refine T_High_ and P_High_ settings, potentially through computational modeling and fuzzy logic, could help maximize APRV’s lung-protective capabilities.

## Recommendation protocol

As defined by the Berlin criteria, ARDS is a form of acute, diffuse, inflammatory lung injury characterized by the rapid onset of respiratory failure within one week of a known clinical insult or new/worsening symptoms. The severity of ARDS is classified based on the degree of hypoxemia, measured by the PaO₂/FiO₂ ratio, with a minimum requirement of 5 cm H₂O positive end expiratory pressure (PEEP): mild (200–300 mmHg), moderate (100–200 mmHg), and severe (<100 mmHg) (9).

The committee’s recommendation *Protocol* is as follows:

Stepwise approach for *initial* APRV settings:

- P_High_
  - Transitioning from conventional mode:
    1. Volume Control (VC) mode: Use P_Plat_ to set P_High_ (Note: an inspiratory hold may be required if a P_Plat_ is not measured with a brief inspiratory time)
    2. Pressure Control (PC) mode: Use PIP to set P_High_
    3. Dual Targeted (DT) mode* (i.e., PRVC): The PIP is used to set P_High_ when there is no spontaneous effort; however, if spontaneous efforts are vigorous and the set V_T_ is exceeded, the PIP will be inaccurate and reflect the PEEP level. This is because these modes display negative proportionality [i.e., the greater the effort (pleural pressure), the lower the applied airway pressure] (Note: May consider changing to a VC or PC mode to obtain an accurate P_Plat_ /PIP pressure, respectively)
    4. High Frequency Oscillatory Ventilation (HFOV) mode: Use Paw plus 2–4 cmH_2_O to set P_High_
  - When APRV is the primary mode at the start of mechanical ventilation, recommendations for *initial* P_High_ ranges:
    1. Mild ARDS: 20–24 cmH_2_O
    2. Moderate ARDS: 25–29 cmH_2_O
    3. Severe ARDS: 26–30 cmH_2_O‡

Note: With decreased C_RS_ or increased E_RS_, V_T_ may be <4 mL/kg; in this case, P_High_ may be increased in 2 cmH_2_O increments to achieve a minimum V_T_ of 5–6 mL/kg.

*Dual-targeted (DT) is a mode in which V_T_ is set, and pressure is automatically regulated to limit airway pressure based on the measured exhaled V_T_ from the previous breath_._ ‡Depending on the patient’s body type and chest wall compliance, a P_High_ greater than 30 cmH_2_O may be needed.

- T_High_
  - Transitioning from a conventional mode:
    1. Set initial T_High_ to match RR on conventional mode to achieve desired MVe. This may be achieved by using the equation: 60/RR – T_Low_. Example: 60/26 = 2.3 s – 0.5 = T_High_ 1.8 s. The T_High_ on APRV requires adjustment once the T_Low_ is confirmed to match MVe from conventional mode. If respiratory acidosis is present on conventional mode, an even higher MVe may be needed and achieved by reducing the T_High_ to increase RR. Note: With decreased C_RS_ / increased E_RS_, V_T_ may be <4 mL/kg; in this case, P_High_ may be increased in 2 cmH_2_O increments to achieve a minimum V_T_ of 5–6 mL/kg.
  - When APRV is the primary mode, set T_High_ at:
    1. Mild ARDS: 2–4 s
    2. Moderate ARDS: 2–3 s
    3. Severe ARDS: 1.5–3 s. Note: Using a very brief T_High_ (e.g., 1.5 s) may be necessary to ensure adequate MVe to maintain normocapnia. Since it takes approximately 1.5 s for the inspiratory airway pressure to reach the distal airspace, the inspiratory flow should be monitored to ensure it returns to baseline (i.e., reaches a no-flow state) (10). If it does not return to baseline, the T_High_ should be increased in small-millisecond steps to achieve this. For example, if a T_High_ of 1.5 s terminates the inspiratory flow and does not return to baseline, the T_High_ may be increased by 0.1-s increments (i.e., 1.6 s) and assessed to determine if the inspiratory flow reaches a no-flow state to prevent inspiratory flow starvation. Later, the T_High_ may be gradually increased as PaCO_2_ decreases_,_ as more lung surface area becomes available for ventilation and stability is achieved over time.
- P_Low_
  - P_Low_ is set to 0 cmH_2_O when transitioning from another mode or when serving as the primary mode.
- T_Low_
  - The T_Low_ is initially set within the ranges listed below, based on lung pathophysiology, when transitioning from another mode or when used as the primary mode. After several breath cycles, once the waveform stabilizes, the T_Low_ should be calculated. This is done by using the Slope_EF_ and Equation 1. The T_Low_ is determined from lung pathophysiology, as measured by Slope_EF,_ when the P_Low_ is set to 0 cmH_2_O. It is established to the fraction of F_EE_ at 75% of the F_PE_. The T_Low_ is adjusted until the target F_EE_ is achieved, is personalized to lung pathophysiology, and reflects lung collapse time constants. For example, if F_PE_ = 60 L/min, the clinician adjusts T_Low_ until expiration ends when the flow is F_PE_ = 45 L/min. [60 L/min × 75% = F_EE_ 45 L/min].
  - Initial T_Low_ settings.
    1. The initial T_Low_ should be based on lung pathophysiology whether APRV is set as the primary mode, or when transitioning from a conventional mode.
      - Normal lung physiology or post-operative respiratory failure: 0.4–0.6 s
      - Mild ARDS: 0.4–0.6 s
      - Moderate ARDS: 0.3–0.5 s
      - Severe ARDS: 0.2–0.4 s
    2. Allow a 15–30 s cycle for the waveform to stabilize
    3. Freeze the screen to analyze the waveform and measure F_PE_
    4. Scroll to the right to measure F_EE_
    5. Calculate F_PE_/F_EE_
    6. Adjust T_Low_ as needed to obtain F_PE_/F_EE_ at 75% (decrease T_Low_ if <75% or increase T_Low_ if >75%) (Equation 1).

### Highlights and monitoring

- The T_Low_ should not be increased solely to obtain a higher V_T_ or to improve PaCO_2_. If V_T_ < 4 mL/kg, increase P_High_ to achieve a minimum of 5–6 mL/kg V_T_. A larger V_T_ results from increasing T_Low_ to terminate less than 75% of F_PE,_ thereby increasing MVe and temporarily reducing PaCO_2_. However, extending the expiratory duration may lead to alveolar instability, allowing alveoli time to collapse and resulting in repetitive alveolar collapse and expansion (RACE)-induced atelectrauma (11–13). Ultimately, progressive loss of lung volume to collapse with improperly set T_Low_ results in an increase in PaCO_2_ as the area for diffusive gas exchange decreases. Although this may temporarily slow alveolar recruitment, adequate ventilation is balanced with progressive airway reopening; T_Low_ cannot be increased since RACE must be prevented at all costs. If V_T_ > 5 mL/kg but MVe is less than on conventional ventilation, T_High_ may be decreased to increase RR and achieve the necessary MVe. As PaCO_2_ improves, T_High_ can be progressively increased, thereby accelerating lung recruitment.
- P_Low_ should not be set above 0 cmH_2_O:
  - Setting a P_Low_ above 0 cmH_2_O alters the Slope_EF,_ so it cannot be used as in spirometry to assess the severity of lung pathophysiology, which is essential for correctly setting T_Low_. The slope of passive exhalation reflects the elastic recoil of the respiratory system and is proportional to changes in elastance (E_RS_) (14). For passive recoil to accurately represent respiratory system mechanics, the flow must not be obstructed by the added expiratory resistance of PEEP, as this alters the flow/time relationship during passive exhalation toward elastic equilibrium.
  - P_Low_ < 0 cmH_2_O decreases expiratory flow rate, thereby reducing CO_2_ removal and leading to hypercapnia.
  - Airway secretions are removed less effectively when P_Low_ is set > 0 cmH_2_O, increasing the risk of ventilator-associated pneumonia.
- PS above P_High_ is not recommended.
  - During APRV, most spontaneous breaths occur during T_High_ at the P_High_ level, also called the continuous positive airway pressure (CPAP) phase. Usually, the patient’s inspiratory effort—primarily driven by diaphragmatic activity—is self-limiting, thereby helping to prevent lung overinflation. However, applying PS above P_High_ adds extra inspiratory pressure and volume during spontaneous breaths. This can significantly increase lung stress and strain, thereby increasing the risk of volutrauma and barotrauma, particularly in injured or heterogeneous lungs, such as in ARDS.
  - PS fundamentally changes the flow dynamics of spontaneous breathing. Without PS, spontaneous inspiratory flow typically follows a sinusoidal pattern, as in natural breathing. Adding PS shifts the flow pattern to a decelerating pattern, more similar to mechanically assisted ventilation than to unassisted spontaneous effort. This change may decrease patient comfort, disrupt normal diaphragmatic mechanics, and potentially impair patient–ventilator synchrony.
  - Key clinical implication: To preserve physiological breathing patterns and minimize the risk of uncontrolled lung overdistension, PS should not be added above P_High_ during APRV. Note: If automatic tube compensation is not available, a maximum PS of 2–5 cmH_2_O may be used to overcome the resistive load of the artificial airway, with the understanding that during spontaneous breaths, the gas flow will be delivered in a decelerating manner, and this pressure will be added on top of P_High._
- Monitoring:
  - Changes in the C_RS_ of the lung or chest wall may require adjustments. For instance, if C_RS_ decreases or the patient is placed in the prone position, thereby reducing V_T_, an increase in P_High_ may be necessary.
  - Assessment of waveforms should be made at a minimum every 6 h.
  - Alarm limits for MVe and V_T_ should be set appropriately to identify changes in C_RS_.
  - If there is an obstruction from secretions, water in the circuit, a wet expiratory filter, or a humidified moisture exchanger, the Slope_EF_ will change from a 45-degree angle to a 90-degree angle, requiring troubleshooting such as suctioning, emptying water from the circuit, or changing wet filters.

## Discussion

This manuscript represents an expert consensus and recommendation document rather than a formal clinical practice guideline. The consensus was developed to synthesize current physiologic principles, experimental data, and available clinical evidence related to APRV, in areas where definitive high-level evidence remains limited. While aspects of APRV are supported by preclinical studies, physiologic investigations, and observational or single-center clinical experiences, large multicenter randomized controlled trials evaluating patient-centered outcomes are currently lacking in several domains. Accordingly, the recommendations presented herein should be interpreted as guidance informed by expert interpretation of lung-protective physiology and the best available evidence, rather than prescriptive standards of care. This consensus aims to provide a structured, physiology-based framework to reduce practice variability, inform the safe and rational application of APRV, and serve as a foundation for future trials designed to more definitively define efficacy, patient selection, and optimal timing of initiation.

The faculty unanimously agreed on the four APRV settings, which closely align with those previously described for the time-controlled adaptive ventilation (TCAV) method. The recommended settings constitute a personalized, protocolized approach that continuously adapts to a patient’s respiratory mechanics and partial pressure of carbon dioxide (PaCO₂). The brief time (T_Low_) at the lower airway pressure (P_Low_), set by non-invasive, breath-to-breath real-time spirometry, to terminate expiratory flow at a precise percentage of the peak expiratory flow rate, thereby preventing alveolar collapse regardless of respiratory system compliance (C_RS_). Adaptation to lung mechanics occurs through T_Low_ adjustment, while adaptation to ventilation and PaCO₂ occurs through time at the upper airway pressure (T_High_) adjustment. By modifying T_High_ and T_Low_ in response to changes in mechanics and ventilation needs, whether improving or declining, the ventilator provides individualized, physiology-guided support that preserves recruitment (T_Low_) while effectively balancing PaCO₂ control (T_High_) and initiating airspace reopening (i.e., the longest T_High_ that maintains adequate ventilation). The upper airway pressure (P_High_) is primarily analogous to a plateau pressure (Pplat) in conventional modes. Modifications to the P_High_ are limited to circumstances in which tidal volume (V_T_) is <4 mL/kg and a significant chest wall component is present, resulting in a decline in overall C_RS_. The recommended protocol has the potential to prevent ARDS by reducing tissue stress and strain through lung stabilization. Its prolonged T_High_ initiates recruitment while the brief, spirometry-guided T_Low_ minimizes dynamic airspace opening and closing, reducing the mechanical injury that drives ARDS development. By limiting these injurious forces, optimally set APRV offers a protective strategy for patients at risk.

### Limitations

This work has several important limitations. First, the recommendations are informed by expert consensus and therefore subject to the inherent biases of expert opinion, including variability in clinical experience, institutional practice patterns, and interpretation of the existing literature. Although efforts were made to include a multidisciplinary and geographically diverse panel, consensus statements cannot fully substitute for prospective empirical validation.

Second, there is a lack of large-scale randomized controlled trial data evaluating the impact of this specific protocol on patient-centered outcomes such as mortality, ventilator-free days, or long-term functional recovery. While the protocol is grounded in established physiologic principles and supported by mechanistic and observational data, definitive conclusions regarding its superiority over alternative strategies cannot be drawn in the absence of adequately powered randomized trials.

Finally, the application of a structured protocol to patients with ARDS is challenged by the marked heterogeneity of the syndrome, including differences in etiology, disease severity, lung recruitability, chest wall mechanics, and temporal evolution. Although the protocol is designed to be adaptive and physiology-guided, its implementation may require clinician judgment and individualized adjustment to account for patient-specific factors that are not fully captured by standardized settings.

## Conclusion

Based on available physiologic data and clinical experience, patients with early, recruitable lung injury—particularly those with moderate to severe hypoxemia, high ΔP, or evidence of tidal derecruitment on conventional ventilation—are most likely to derive benefit. Early initiation, before the establishment of extensive alveolar collapse, edema, and fibroproliferation, may optimize the protocol’s ability to stabilize alveoli, reduce cyclic opening and closing, and limit ventilator-induced lung injury.

However, given the heterogeneity of ARDS and the absence of large, definitive randomized trials addressing these questions, we intentionally avoid rigid inclusion criteria or timing thresholds. Instead, we emphasize a physiology-guided approach in which APRV is considered when conventional strategies fail to maintain adequate oxygenation or lung stability without injurious pressures, and when real-time assessment of respiratory mechanics suggests ongoing derecruitment. Future prospective studies stratified by ARDS phenotype, timing of initiation, and recruitability are needed to define patient selection and optimal timing more precisely.

**Figure 11:**
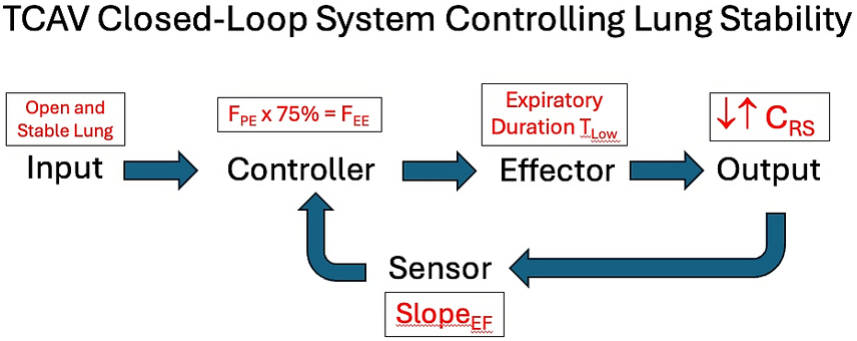
The time-controlled adaptive ventilation (TCAV) method for setting airway pressure release ventilation (APRV) is a closed-loop system guided by changes in respiratory system compliance (C_RS_), which helps maintain lung stability as lung conditions change. The input to this closed-loop system is a stable, open lung. The controller uses Equation 1 (F_PE_ x 75% = F_EE_) to calculate the effector: expiratory duration (T_Low_). T_Low_ must be sufficiently brief to prevent alveoli with rapid collapse time constants from collapsing. The physiological output is the change in C_RS_. The slope of the expiratory flow curve (Slope_EF_) acts as the sensor, measuring breath-by-breath changes in C_RS_. Slope_EF_ functions like spirometry to identify lung pathophysiology. The sensor adjusts the controller to maintain the desired input (5). When C_RS_ decreases, Slope_EF_ becomes steeper, leading T_Low_ to be shortened to prevent alveoli with fast time constants from collapsing.

The goal of this consensus guidelines conference was to recommend APRV settings that have demonstrated excellent clinical results across multiple hospitals when used by experts over many years. It outlines recommendations for setting and adjusting the four APRV parameters (P_High_, T_High_, P_Low_, T_Low_) and discusses the scientific evidence supporting these suggestions. Our recommendations closely align with the TCAV method for setting and adjusting the APRV mode and function as a closed-loop system, maintaining an open and stable lung in response to changing lung pathophysiology (Figure 11). The faculty hopes that these guidelines will serve as a starting point for clinicians interested in adopting APRV for their patients and guide more homogenous studies investigating APRV.

## Data Availability

The original contributions presented in the study are included in the article/Supplementary material, further inquiries can be directed to the corresponding authors.
