# Correction: Expert recommendations for setting and adjusting airway pressure release ventilation based on clinical experience and basic science evidence

**DOI:** 10.3389/fmed.2026.1832064

**Published:** 2026-03-25

**Authors:** Frontiers Production Office

**Affiliations:** Frontiers Media SA, Lausanne, Switzerland

**Keywords:** airway pressure release ventilation, APRV, ARDS, consensus recommendations, TCAV, time controlled adaptive ventilation, ventilator induced lung injury, VILI

**Supplementary Images 1–9** and **Supplementary Tables 1–13** were erroneously published with the original version of this paper. These files have now been removed and replaced with **Supplementary Files 1–7**.

The original version of this article has been updated.

