# Supplementary material for: Expert recommendations for setting and adjusting airway pressure release ventilation based on clinical experience and basic science evidence
Source: Front Med (Lausanne). 2026 Feb 3;13:1741129. doi: 10.3389/fmed.2026.1741129 (PMC12909506; doi:10.3389/fmed.2026.1741129)
Supplement: Supplementary file 1 [file Supplementary_file_1.pdf]

Supplementary File 1 - Faculty Bio-Sketches

Dr. Hassan Al-Khalisy completed his residency in Internal Medicine at UPMC Harrisburg, followed by a fellowship in Pulmonary and Critical Care at SUNY Upstate. During his fellowship, he developed a keen interest in respiratory failure, particularly in patients with ARDS. His passion for this field led him to join the Nieman Physiology and Critical Care Lab, where he worked as a research fellow under the mentorship of Dr. Nader Habashi, Penny Andrews, and Professor Nieman.

Currently, Dr. Al-Khalisy serves as the Medical Director of the Medical Intensive Care Unit at East Carolina University Health System and also as the Director of Adult Respiratory Services. He specializes in managing patients with respiratory failure, employing the APRV mode and the TCAV method to achieve remarkable outcomes. His contributions have trained numerous fellows nationwide, who now successfully implement TCAV.

Dr. Al-Khalisy and his team have developed a comprehensive Mechanical Ventilation course, aimed at Pulmonary and Critical Care fellows, and have expanded it to include respiratory therapists and surgical intensivists. This program continues to grow in scope and impact. Additionally, he has collaborated on several publications with the Nieman group since his time at SUNY Upstate. Dr. Al-Khalisy remains dedicated to providing expert care across multiple units, including the CICU, and manages patients requiring ECMO.

Penny L. Andrews, RN, BSN, is an experienced critical care nurse with over 30 years in the field, currently involved in clinical research at the R Adams Cowley Shock Trauma Center in Baltimore, MD—America's largest standalone trauma center. She has extensive expertise in caring for and managing critically ill and injured patients, especially in the area of mechanical ventilation.

Her clinical and research interests focus on preventing acute respiratory distress syndrome (ARDS), guided by basic science research and animal models of lung injury developed through collaboration with Nader M. Habashi and Gary F. Nieman. Penny specializes in applying mechanical ventilation strategies, especially Airway Pressure Release Ventilation (APRV) using the Time-Controlled Adaptive Ventilation (TCAV™) method.

Penny has authored publications in peer-reviewed journals and has presented her work at both national and international conferences. She also plays a key role in implementing the TCAV method of APRV in brain-dead organ donors to improve the viability of donated organs, managing TCAV for thousands of patients.

Joaquin Araos, DVM, PhD, DACVAA, is an Associate Professor of Veterinary Anesthesia at Cornell University's College of Veterinary Medicine. His research bridges veterinary and human critical care, focusing on the cardiopulmonary physiology of

mechanical ventilation and extracorporeal support. Dr. Araos has developed translational large-animal models of acute respiratory distress syndrome (ARDS), including long-duration ECMO support in pigs, to study the interplay between mechanical strain, lung homogenization, and right-ventricular function. His work integrates advanced imaging, hemodynamic waveform analysis, and finite-element modeling to characterize regional lung strain and perfusion. In recent years, Dr. Araos has completed research in equine anesthesia and mechanical ventilation, where his group demonstrated that applying Time-Controlled Adaptive Ventilation (TCAV) during general anesthesia in supine horses optimizes oxygenation and regional and global lung aeration. He collaborates extensively with Drs. Gary Nieman and Nader Habashi on the mechanistic basis of TCAV, contributing experimental evidence supporting gradual recruitment as a key process in mitigating ventilator-induced lung injury. Dr. Araos has authored more than 60 peer-reviewed publications and has received support from intramural programs, the Zoll Foundation, and the NIH for work on ECMO, lung mechanics, and novel cardiopulmonary monitoring.

Jason H.T. Bates, PhD, DSc, has spent nearly four decades in academic biomedical research, first at McGill University and then at the University of Vermont. His research interests focus on the mechanical properties of the lung in health and disease. Dr. Bates extensively utilizes both advanced bioengineering experimental methods and computational modeling, and he has published over 350 peer-reviewed publications, most of which relate to lung physiology and biomedical engineering. He has also authored a single-author book, "Lung Mechanics: An Inverse Modeling Approach" (Cambridge University Press, 2009), which provides comprehensive coverage of the mathematics and physics needed to understand lung mechanics from a quantitative perspective. Dr. Bates has recently undertaken extensive work on the TCAV mode of mechanical ventilation in collaboration with Prof. Gary Nieman, with whom he has coauthored 19 papers. Drs. Bates and Nieman have been co-investigators on an NHLBI R01 grant since 2018. Dr. Bates' most notable contribution to the investigation of TCAV as a protective mode of mechanical ventilation in acute lung injury has been deriving support for the hypothesis that the efficacy of TCAV is due to its very brief expiratory duration. This prevents lung units from having enough time to derecruit during expiration, thereby avoiding the subsequent inspiratory recruitment that is so damaging to lung tissues.

Professor Luigi Camporota has worked extensively in the fields of acute respiratory distress syndrome (ARDS), mechanical ventilation, and extracorporeal membrane oxygenation (ECMO) for over two decades. His research has concentrated on ventilator-induced lung injury (VILI), lung-protective ventilation, the mechanical power of VILI, and personalized approaches to managing respiratory failure.

He has collaborated with Professors Nader Habashi, Gary Nieman, and Luciano Gattinoni to investigate airway pressure release ventilation (APRV), mechanical ventilation-induced regional lung stress, and ECMO weaning strategies. Studies with Habashi and Nieman have examined the distribution of mechanical breath energy within the lungs, contributing to a better understanding of VILI progression. Their findings have refined time-controlled ventilation strategies, including the Time-Controlled Adaptive Ventilation (TCAV) approach, which aims to enhance alveolar stability and reduce lung injury.

Beyond mechanical ventilation, he has studied the interactions among ARDS, lung mechanics, and extracorporeal support, examining how different respiratory support strategies—from ECMO to low-flow CO_2 removal—can minimize ventilator-induced lung stress. His research with Gattinoni has investigated lung mechanics and the physiological determinants of oxygenation and ventilation in critically ill patients.

Professor Camporota has authored over 270 peer-reviewed publications and contributed to large NIHR- and EPSRC-funded research projects. He has participated in multi-centre clinical trials, including ROMEO, PHIND, MARCH, and REALIST, which study new interventions for ARDS, mechanical ventilation, and ECMO management. His work in translational research also encompasses digital twin modelling for mechanical ventilation, which integrates computational physiology with clinical data to enhance decision-making at the bedside.

He is the Co-CI of an upcoming UK multicentre trial comparing TCAV with conventional lung-protective ventilation, set to begin recruitment in 2025.

He has been actively involved in professional societies, having previously served as Chair of the Acute Respiratory Failure Section of the European Society of Intensive Care Medicine (ESICM) and contributed to the development of NHS England’s national ECMO and mechanical ventilation guidelines. His work has supported UK NICE recommendations on ARDS management, ensuring that lung-protective ventilation strategies are grounded in the latest physiological evidence.

John Downs, M.D., graduated from the University of Illinois in 1965 and the University of Florida School of Medicine in 1969. During his residency and fellowship at the University of Florida, he introduced the concept of spontaneous breathing during mechanical ventilation for adult patients, which is known as intermittent mandatory ventilation (IMV). While serving as a major and director of the surgical ICU at Wilford Hall USAF Hospital, he reported the first case using high-level positive end-expiratory pressure and co-authored the first series of patients receiving high PEEP levels shortly thereafter. In 1987, he introduced airway pressure release ventilation (APRV) to support patients requiring mechanical ventilatory assistance. He co-authored multiple studies

involving APRV in animals and patients in the 1990s and a few years later. He served as a professor and acting chair of Anesthesiology at Ohio State University, professor and chair of Anesthesiology and Critical Care Medicine, and Emeritus Professor at the University of South Florida, and is currently a courtesy Professor in Anesthesiology and Critical Care Medicine at the University of Florida.

Nader M. Habashi, MD, FACP, FCCP, Dr. Nader M. Habashi is the Hamish and Christine Osborne Distinguished Professor of Medicine at the University of Maryland School of Medicine. Nader also serves as Professor of Surgery at SUNY Upstate Medical University and as a Researcher at the Cornell University College of Veterinary Medicine. He previously served as Medical Director of the Multi-Trauma ICU at the R Adams Cowley Shock Trauma Center in Baltimore, Maryland—the nation's largest free-standing trauma hospital, where he remains an active attending physician.

A board-certified physician in Internal Medicine, Pulmonary Medicine, Critical Care Medicine, and Neurocritical Care, Dr. Habashi brings over three decades of clinical, physiologic, and research experience to the field of intensive care. His career has focused on understanding the fundamental principles of respiratory physiology and respiratory mechanics as they apply to the care of critically ill and injured patients.

Dr. Habashi is internationally recognized for his pioneering work in mechanical ventilation and the prevention of ventilator-induced lung injury (VILI). His research has centered on Airway Pressure Release Ventilation (APRV) and the Time-Controlled Adaptive Ventilation (TCAV™) method—a physiologic approach to mechanical ventilation designed to stabilize alveoli, preserve lung homogeneity, and prevent acute lung injury.

Through extensive basic science studies, animal models of lung injury, and translational clinical research, Dr. Habashi has advanced understanding of how time-dependent control of airway pressure interacts with lung mechanics and gas exchange. His work continues to refine strategies that both protect and recruit the injured lung while maintaining cardiovascular stability.

Dr. Habashi has authored numerous peer-reviewed publications, book chapters, and scientific reviews, and has been an invited speaker at major national and international conferences. His lifelong commitment to integrating physiology with bedside practice has shaped modern approaches to mechanical ventilation and continues to influence critical care medicine worldwide.

Dr. Aurélien Ledoux, MD, graduated as both a bioengineer and a physician from the Université catholique de Louvain in Belgium. He completed his specialization in

anesthesiology and critical care medicine and earned a ministerial diploma qualifying him to lead intensive care units.

He currently works in a multidisciplinary intensive care unit within the HELORA hospital network in Belgium. His research interests include in vivo perfusion monitoring and mechanical ventilation in critically ill patients. His involvement with the TCAV approach to APRV began during the COVID-19 pandemic, leading to his ongoing collaboration with Dr. Nader Habashi, Penny Andrews, and Professor Nieman.

Maria Madden has over 30 years of experience as a respiratory therapist, researcher, ECMO specialist, and educator at the University of Maryland Medical Center/R. Adams Cowley Shock Trauma Center. She also works as a clinical specialist at ICON. Throughout her career, Maria has collaborated with Dr. Nader Habashi and Penny Andrews, gaining extensive experience in the use of APRV-TCAV for patient treatment. She is a national speaker on APRV-TCAV and has published research on its application.

In addition to her clinical roles, Maria has worked as an adjunct professor for various respiratory therapy programs. Her interests include critical care, APRV-TCAV, mechanical ventilation strategies, managing patients with spinal cord injuries, and the treatment and prevention of ARDS. Currently, she is serving her second term on the Board of Directors for the American Association of Respiratory Care.

Dr. Manjunath Markandaya has practiced as an intensivist and neuro-intensivist for over 15 years. He completed his Neurocritical Care fellowship at The Johns Hopkins Hospital and his Surgical Critical Care fellowship at the R Adams Cowley Shock Trauma Center, where he trained under Dr. Habashi in the intricacies of APRV-TCAV. Since then, he has primarily utilized APRV-TCAV as the preferred ventilation mode for his critically ill patients, achieving very positive outcomes. Dr. Markandaya has established and led three different neurocritical care units in New York and North Carolina, promoting the institution-wide adoption of APRV-TCAV as a key ventilation strategy. On average, he cares for over 500 ventilated patients each year and approximately 200 patients with ARDS annually. He has a particular interest and expertise in applying APRV-TCAV for critically ill patients with neurological injuries.

Dr. Toni Manougian is a board-certified anesthesiologist and intensivist with 16 years of experience in managing ICU care for critically ill trauma, surgical, and cardiothoracic patients. She completed her anesthesiology residency at St. Vincent's Catholic Medical Center in New York, followed by a critical care fellowship at NewYork-Presbyterian Hospital, Columbia University Medical Center. Dr. Manougian is the Section Chief of Critical Care Anesthesiology at Westchester Medical Center, specializing in mechanical ventilation strategies for respiratory failure through Time-Controlled Adaptive Ventilation (TCAV).

Her interest in TCAV began during the COVID-19 pandemic when she observed significant improvements in oxygenation and lung compliance through this approach while collaborating with Dr. Nader Habashi and Penny Andrews. For the past five years, she has been teaching this model and applying TCAV in the trauma and surgical ICUs. In 2023, she earned an MBA from Brandeis University, with a concentration in leadership and driving change in healthcare. Her MBA capstone project investigated strategies for implementing change in healthcare, using TCAV adoption as a model. She continues to apply these principles to refine ICU protocols, enhance ventilator management, and improve care for critically ill patients.

Professor Gary Nieman: For over five decades, Professor Nieman has studied various aspects of acute respiratory distress syndrome (ARDS) pathophysiology and has tested numerous novel pharmacological treatments, many of which have been patented. The past decade has been dedicated to exploring ventilator-induced lung injury (VILI), which is known to increase ARDS-related morbidity and mortality. Specifically, he has focused on the pathophysiology of VILI in the microenvironment and mechanical ventilation strategies to address these mechanisms.

In collaboration with Drs. Nader Habashi, Penny Andrews, Jason Bates, Hassan Al-Khalisy, and others demonstrated that pulmonary surfactant deactivation, a hallmark of ARDS pathology, significantly alters dynamic alveolar inflation, resulting in tissue damage due to the collapse and reopening of alveoli, as well as overdistension of alveolar ducts adjacent to regional atelectasis. These findings reveal that regional lung collapse acts as a stress multiplier, concentrating mechanical breath energy on adjacent healthy tissue and thereby driving progressive VILI. Additionally, the study examines how ARDS impacts the viscoelastic properties of lung tissue, rendering lung inflation time- and pressure-dependent. This indicates that it takes longer for lung tissue to open and less time to collapse at any given airway pressure. This led to the hypothesis that longer ventilator time, extended inspiratory duration, and brief expiratory duration could rapidly stabilize lung tissue, followed by a gradual ratcheting open over hours or days.

This research, in conjunction with the clinical work by Drs. Nader Habashi, John Downs, and others contributed to the development of the Time-Controlled Adaptive Ventilation (TCAV) method for setting and adjusting the airway pressure release ventilation (APRV) mode. Professor Nieman has authored over 180 peer-reviewed publications, including 53 studies on the role of time in protective mechanical ventilation, and holds six patents. He has presented extensively at international research symposia, contributed to several book chapters, and recently co-authored a book with Dr. Habashi on "***The Applied Physiology of Protective Mechanical Ventilation*** “ (doi.org/10.1007/978-3-031-66352-9). Drs. Nieman and Bates have been co-investigators on an NHLBI R01 grant since 2018.

Professor Luis Felipe da Fonseca Reis has been clinically active in two Adult Intensive Care Units for over 20 years, investigating mechanical ventilation in various clinical contexts. His research centers on Protective Mechanical Ventilation, ventilator-induced lung injury (VILI), and tailored approaches to managing respiratory failure. In the past six years, he has focused on the clinical application, development of care protocols, and research of Time-Controlled Adaptive Ventilation (TCAV) during airway pressure release ventilation (APRV).

During the COVID-19 pandemic, this strategy became routine in managing patients with ARDS and continues to be a method for preventing ARDS in individuals with risk factors or established clinical criteria. During this period, he supervised Master's and Doctoral students studying Time-Controlled Adaptive Ventilation (TCAV) in conjunction with airway pressure release ventilation (APRV), presenting papers at national and international conferences on the topic, and even receiving awards in Brazil for these contributions.

Today, regarding Time-Controlled Adaptive Ventilation (TCAV) in the context of airway pressure release ventilation (APRV), he has studied the impact of patient maintenance time on this strategy. He has also focused on various bedside monitoring techniques during TCAV. Since 2003, he has actively participated in organizations such as the Brazilian Association of Cardiorespiratory Physiotherapy and the Brazilian Association of Physiotherapy in Intensive Care, as well as the Brazilian Association of Intensive Care Medicine since 2010.

Professor Louise Rose MBE is a Professor and Research Division Head at King's College London. She also holds an honorary Professorship in the Departments of Critical Care and Lane Fox Respiratory Unit at St Thomas' Hospital in London. Before this, she was an Associate Professor at the University of Toronto. Her research focuses on improving outcomes and experience of patients with acute and chronic respiratory failure, including those receiving mechanical ventilation in intensive care and at home in the community. Prof Rose is a senior or principal investigator on numerous randomized controlled trials in both critical care and home ventilation funded in Canada, Australia, and the United Kingdom. This includes an NIHR-funded trial of Airway Pressure Release ventilation, currently recruiting a target of 710 participants. Prof. Rose is also experienced in systematic reviews, meta-analyses, implementation science, process evaluations, and the development of core outcome sets, critical methodologies that inform clinical trial conduct.

Professor Rose has authored over 290 peer-reviewed publications and been awarded more than 110 peer-reviewed research grants from government and charitable organisation funders in Canada, the UK, Australia, and New Zealand, totaling over £22

million. Professor Rose is active in various professional societies, including serving as the current chair of the Research Division of the UK Intensive Care Society. In 2023, she received the Member of the British Empire (MBE) award for her work in digital innovations that supported patients and families during the COVID-19 pandemic.

Joshua Satalin, BS, is a researcher at Cornell University with 14 years of experience developing and conducting large animal models of acute lung injury and sepsis. His expertise lies in the management of mechanically ventilated patients using airway pressure release ventilation (APRV), specifically the time-controlled adaptive ventilation (TCAV) methodology. He has contributed extensively to translational research aimed at preventing and treating acute respiratory distress syndrome (ARDS), ventilator-induced lung injury (VILI), and sepsis. His work focuses on mechanistic studies that bridge alveolar micromechanics with clinical outcomes, and he has co-authored over 35 peer-reviewed publications in high-impact journals, including *JAMA Surgery, Intensive Care Medicine Experimental, Annals of Translational Medicine,* and *Critical Care Medicine*. He is skilled in designing and executing complex in vivo studies, developing clinically relevant ARDS and sepsis models, and applying physiologically informed ventilation strategies to improve survival and recovery. As a senior research specialist, he brings a unique combination of technical expertise in large animal experimentation, ventilatory physiology, and translational research. These skills directly support my role in multidisciplinary teams developing novel interventions to improve outcomes in critically ill patients.

Joseph Shiber, MD, FACP, FACEP, FNCS, FCCM, trained at UMMS/R Adams Cowley Shock Trauma Center in the 1990s and has successfully applied APRV-TCAV over the past 25 years in various settings (ED, Medical ICU, Surgical ICU, Neuro ICU, Trauma Resuscitation Unit) to rescue patients with severe ARDS and to prevent its occurrence. Using APRV-TCAV as the primary mode of mechanical ventilation while serving as the Medical Director of several ICUs has demonstrated reduced rates of ARDS and avoidance of ECMO. Additionally, when consulted for ECMO evaluation in critical ARDS cases, it has been effective in avoiding the need for ECMO in over 75% of patients, with a survival rate exceeding 80%. He is currently the ECLS/Advanced Lung Service Director at UF Health-Jacksonville and a Professor of Neurology, Surgery, and Emergency Medicine, where he cares for over 100 patients with severe ARDS each year.

Professor Pedro Leme Silva has studied experimental ARDS and emphysema during mechanical ventilation for over fifteen years. His research focuses on ventilator-induced lung injury (VILI), lung-protective ventilation, mechanical power in VILI, and individualized methods for managing respiratory failure.

He has collaborated with Professors Nader Habashi, Gary Nieman, and Penny Andrews to investigate the Time-Controlled Adaptive Ventilation (TCAV) approach during airway pressure release ventilation (APRV). We conducted several pre-clinical studies involving

1) pulmonary and extrapulmonary ARDS, 2) pneumonia models using live bacteria instillations, and 3) emphysema induced by elastase instillations. In the ARDS models, the expiratory time (T_{Low}) was set using the equation: peak expiratory flow (P_{EF}) × Cofactor (0.75) = termination of expiratory flow (T_{EF}). In the pulmonary ARDS model, TCAV resulted in more pronounced beneficial effects on the expression of biomarkers related to overdistension and extracellular matrix homeostasis compared to volume-controlled ventilation (VCV). In the pneumonia model, at the same tidal volume (V_T) and mean airway pressure, TCAV was associated with less lung damage, lower gene expression of pro-inflammatory mediators, and reduced bacteremia compared to VCV. In the emphysema model, at similar V_T, we found that the equation to set T_{Low} needed to be modified to extend T_{Low} since lung recoil was reduced (P_{EF} x 25% = T_{EF}). This modification was associated with decreased alveolar heterogeneity, reduced lung inflammation and edema, and lower gene expression of biological markers related to ventilator-induced lung injury (VILI). It improved right ventricular (RV) performance compared to the previous equation (P_{EF} x 75% = T_{EF}).

Beyond mechanical ventilation, he has studied innovative therapies for pulmonary arterial hypertension, the brain-lung axis during mechanical ventilation, and the effects of anesthetic agents on cardiopulmonary interactions during mechanical ventilation. Recently, he has worked on retrospective studies related to COVID-19 and predictive indices for endotracheal intubation, as well as the use of NIV, HFNO, and their association with clinical outcomes in COVID-19 patients.

He has been actively involved in societies such as the American Thoracic Society (ATS) since 2010 and the Brazilian Physiological Society since 2008. Recently, he joined the Practical Guidelines on Mechanical Ventilation 2024 in Brazil.

Dr. Tero Varpula completed his residency in anesthesiology at the University of Helsinki and later participated in a Scandinavian postgraduate training program in Critical Care. He holds a European Diploma in Intensive Care Medicine.

Dr. Varpula has conducted several studies on respiratory failure. He carried out a randomized controlled trial (RCT) comparing Airway Pressure Release Ventilation (APRV) to Synchronized Intermittent Mandatory Ventilation (SIMV) in patients with severe Acute Respiratory Distress Syndrome (ARDS). He defended his thesis based on these studies in 2004. He has authored 43 peer-reviewed papers across various fields of critical care and serves as a professor of Intensive Care Medicine.

Dr. Varpula currently serves as the Medical Director of the Intensive Care Unit at Helsinki University's Jorvi Hospital and the Burn Unit. He has also led the National Mechanical Ventilation course for several years, chaired the writing group for the National Good

Clinical Practice Guidelines on acute respiratory failure, and acted as the Medical Advisor for the Finnish National ICU registry.
