# Supplementary material for: Expert recommendations for setting and adjusting airway pressure release ventilation based on clinical experience and basic science evidence
Source: Front Med (Lausanne). 2026 Feb 3;13:1741129. doi: 10.3389/fmed.2026.1741129 (PMC12909506; doi:10.3389/fmed.2026.1741129)
Supplement: Supplementary file 2 [file Supplementary_file_2.pdf]

Supplementary File 2

Role of Time and Pressure in Protective Mechanical Ventilation

Consensus Conference Guidelines Faculty Publications

Gaver D. *PNAS* 2025; 122	doi.org/10.1073/pnas.2419374122
Vicenti C, Araos J. *Vet Anaesth Analg* 2025	doi.org/10.1016/j.vaa.2025.06.007
Nieman, Habashi. *Springer Nature* 2024	ISBN-13: 9783031663512
Bates JHT. *Crit Care* 2024;28	doi.org/10.1186/s13054-024-05112-w
Bates JHT. *AJRCCM* 2024;209	DOI: 10.1164/rccm.202307-1292VP
Al-Khalisy H. *Resp Res* 2024;25:37	DOI: 10.1186/s12931-023-02615-y
Kingsley J. *Perfusion* 2024;0:1	DOI: 10.1177/02676591241227167
Lutz MR. *J Clin Med* 2024;13:2690	doi.org/10.3390/jcm13092690
Ramcharran H. Resp Care 2024	DOI: https://doi.org/10.4187/respcare.11745
Bates JHT. *Ann Biomed Eng* 2023	doi.10.1007/s10439-023-03186-1
Cruz A. *Military Med* 2023;188	DOI: 10.1093/milmed/usad059
Camporota L. *Curr Opin Crit Care* 2023;30	DOI:10.1097/MCC.0000000000001123
Nieman *Frontiers Physiol* 2023;14	doi: 10.3389/fphys.2023.1287416
Nieman *J Crit Med* 2023;12	doi.org/10.3390/jcm12144633
Habashi N. *Crit Care Med* 2023;	DOI: 10.1097/CCM.0000000000006018
Ma H. *Front Netw Physiol* 2023;3	doi: 10.3389/fnetp.2023.1257710
Habashi N. *Crit Care Med* 2022;50	doi: 10.1097/CCM.0000000000005403
Silva P. *JAP* 2022;132	doi:10.1152/japplphysiol.00689.202
Andrews P. *Frontiers Physiol* 2022;13,	doi: 10.3389/fphys.2022.928562
Oliveira *JAP* 2022;132,	doi:10.1152/japplphysiol.00689.2021
Ramcharran *JAP* 2022	doi: 10.1152/japplphysiol.00312.2022
Nieman. *Crit Care* 2022;26,	doi: 10.1186/s13054-022-04105-x
Nieman Frontiers Physiol 2022	DOI 10.3389/978-2-83250-264-8
Habashi N. *Crit Care Med* 2022;50	doi: 10.1097/CCM.0000000000005403
Habashi N. *JAP* 2021;130	doi:10.1152/japplphysiol.00742.2020
Beretta E. *Front Physiol* 2021;12,	doi: 10.3389/fphys.2021.781874
Magalhaes *Crit Care Med* 2021;49,	DOI: 10.1097/CCM.0000000000004675
Janssen *BMC Pulm Med* 2021;21	doi.org/10.1186/s12890-021-01545-z
Bates JHT. *Crit Care Explor* 2020;2	DOI: 10.1097/CCE.0000000000000299
Nieman. *Ann Inten Care*. 2020;10,	doi.org/10.1186/s13613-019-0619-3
Nieman. *Frontiers Physiol* 2020;11,	doi: 10.3389/fphys.2020.00227
Mahajan M. *Inten Care Med Exp* 2019;7:27,	doi.org/10.1186/s40635-019-0250-5

Silva PL *Crit Care Med* 2018; 46,	DOI: 10.1097/CCM.0000000000003078
Nieman. *Crit Care* 2018;22,	doi.org/10.1186/s13054-018-2051-8
Nieman. *J Trauma ACS* 2018;85,	DOI: 10.1097/TA.0000000000002050
Satalin J. *Trends Anaesth* 2018;22	doi.org/10.1016/j.tacc.2018.05.007.
Jain S. *Inten Care Med Exp* 2017;5,	DOI 10.1186/s40635-017-0138-1
Nieman. *JAP* 2017;122,	doi:10.1152/japplphysiol.00123.2017.
Nieman. *Inten Care Med Exp* 2017;5,	doi.org/10.1186/s13054-018-2051-8
Jain S. *Inten Care Med Exp* 2016;4,	DOI 10.1186/s40635-016-0085-2
Kollisch *J Ped Surg* 2016;52	doi.org/10.1016/j.jpedsurg.2016.10.020
Kollisch *Inten Care Med Exp* 2015;3	DOI 10.1186/s40635-015-0071-0
Kollisch *JAMA Surg* 2015, 151,	doi:10.1001/jamasurg.2015.2683
Smith BJ. *JAP* 2015;118,	doi:10.1152/japplphysiol.00902.2014
Nieman. *JAP* 2015;119,	doi:10.1152/japplphysiol.00659.2015.
Kollisch *JACS* 2014;219,	doi.org/10.1016/j.jamcollsurg.2014.09.011
Kollisch *JAMA Surg* 2014, 149,	doi:10.1001/jamasurg.2014.1829
Roy S. *Shock* 2013;39	DOI: 10.1097/SHK.0b013e31827b47bb
Roy S. *Shock* 2013;40	DOI: 10.1097/SHK.0b013e31829efb06
Andrews P. *J Trauma ACS* 2013;75:635,	DOI: 10.1097/TA.0b013e31829d3504
Emr B. *JAMA Surg* 2013,	doi:10.1001/jamasurg.2013.3746
Nieman *Crit Care Med* 2013;41	DOI: 10.1097/CCM.0b013e31828ce91b
Roy. *J Trauma ACS* 2012;73,	DOI: 10.1097/TA.0b013e31825c7a82
Albert S. J Surg Res 2011;166,	doi:10.1016/j.jss.2010.10.022
Albert S. *JAP* 2008;103	doi:10.1152/japplphysiol.90735.2008
Habashi N. *Crit Care Med* 2005;33,	DOI:10.1097/01.CCM.0000155920.11893.37
Carney D. *Crit Care Med* 2005;33	DOI: 10.1097/01.CCM.0000155928.95341.BC
Varpula T, *Acta Anaes Scand* 2004;48	doi: 10.1111/j.0001-5172.2004.00411.x
