# Supplementary material for: Expert recommendations for setting and adjusting airway pressure release ventilation based on clinical experience and basic science evidence
Source: Front Med (Lausanne). 2026 Feb 3;13:1741129. doi: 10.3389/fmed.2026.1741129 (PMC12909506; doi:10.3389/fmed.2026.1741129)
Supplement: Supplementary file 3 [file Supplementary_file_3.pdf]

**Supplementary File 3**

# Science Supporting the Guideline Recommendations

## Pathophysiology of ARDS and VILI

*VILI Pathogenesis*

A unifying theory of the pathogenesis of acute respiratory distress syndrome (ARDS) and ventilator-induced lung injury (VILI) is essential for understanding how mechanical ventilation can help restore the dynamic behavior of alveoli and ducts in the injured lung (Fig. S1).[1] The normal lung (*Normal Parenchymal Micromechanics*) becomes compromised in conditions such as *sepsis, pneumonia*, and *hemorrhagic shock*, especially when combined with ARDS, which has a notably high mortality rate (Fig. S1).[2] The initial inflammatory injuries in these conditions *increase endothelial permeability*, disrupting the lung's fluid balance as described by the Starling equation.[3] The resulting *pulmonary edema* plays a key role in driving progressive acute lung injury. Edema impairs *pulmonary surfactant function*, leading *to abnormal parenchymal micromechanics* during *mechanical ventilation* and thereby promoting progressive VILI. This process of VILI development is called a *permeability-originated obstruction response (POOR)*.[4] [5] POOR worsens with each mechanical breath, resulting in a *continuous decrease in end-expiratory lung volume (EELV)* and increased lung tissue damage from VILI (Fig. S1, red arrow). This aggravates existing lung injury, creating a *VILI Vortex*, which makes escape increasingly difficult.[6] Patient outcomes are unlikely to improve unless a reliable strategy for breaking this cycle can be developed.

The rationale for the ARDSnet low tidal volume ($V_T$) lung-protective strategy is that ARDS causes collapse of the lung's dependent regions. In contrast, the remaining non-dependent regions stay normal and inflated. The functional part of the lung is thus inherently normal but smaller than expected, a condition known as the "baby lung." [7] The obvious concern with the 'baby lung' is that inflating it with a normal $V_T$ could cause volutrauma from overdistension. This has led to the idea that $V_T$ should be lower than normal in ARDS. However, low $V_T$ can lead to long-term atelectasis, which increases the risk of pneumonia [8], fibrosis [9-11], pulmonary vascular resistance [12, 13], and edema. [14] Atelectasis also causes patient-ventilator desynchrony via activation of

mechanosensory, $PO_2$, $PCO_2$, and pH receptors [15-19], which is associated with high

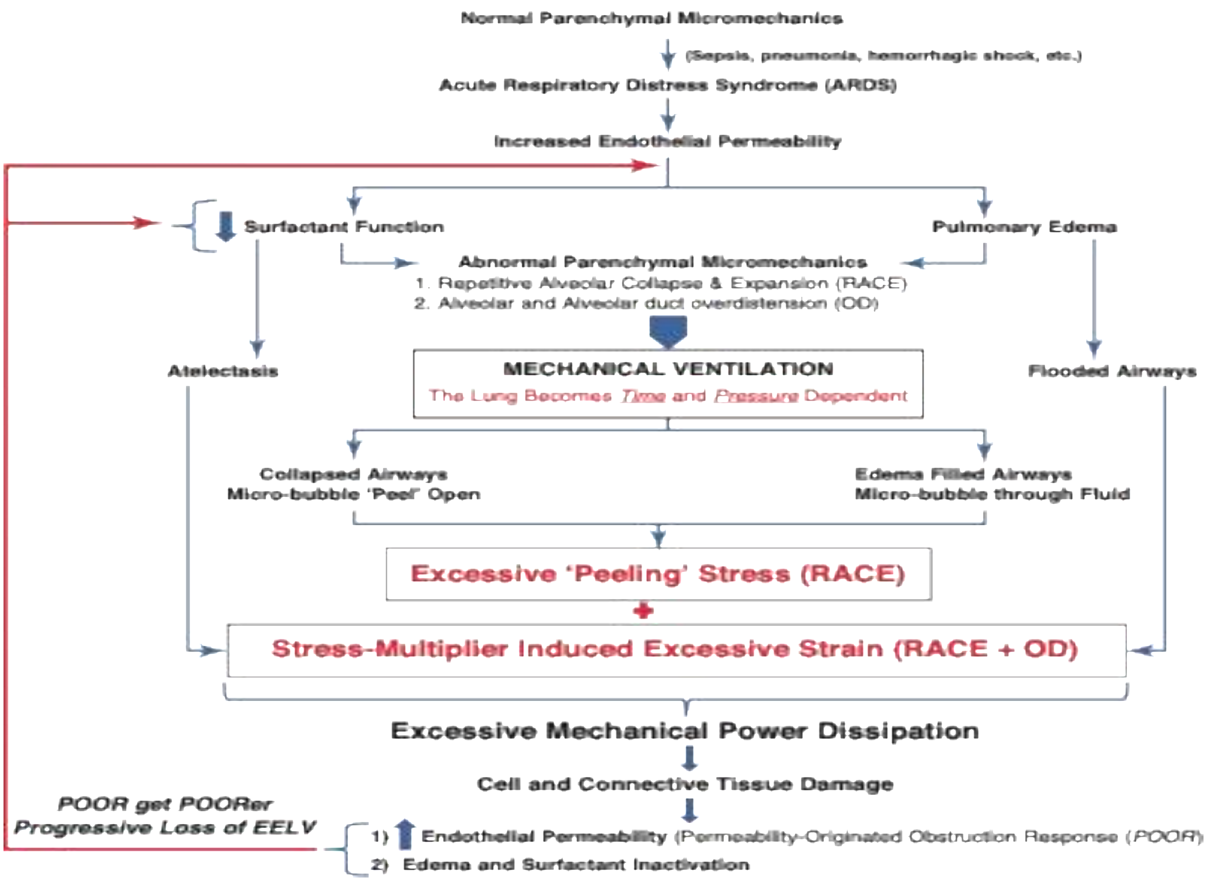


**Figure S1**. The proposed unifying hypothesis outlines the mechanisms by which the pathophysiology of acute respiratory distress syndrome (ARDS) predisposes the lung to secondary ventilator-induced lung injury (VILI). Primary injuries such as sepsis, pneumonia, or hemorrhagic shock initiate a cascade of events leading to ARDS. The inflammatory response to these insults increases **endothelial permeability**, driving two key processes: **impaired surfactant function** and **pulmonary edema**. These processes culminate in **abnormal parenchymal micromechanics** at the level of the alveoli and alveolar ducts.

These abnormalities promote **repetitive alveolar collapse and expansion (RACE)**, leading to **atelectrauma**, and **alveolar/ductal overdistension (OD)**, contributing to **volutrauma**. The combination of collapsed and fluid-filled alveoli reduces the lung's gas-exchange surface area, leading to hypoxemia and hypercapnia and often requiring **mechanical ventilation (MV)**.

Mechanical ventilation interacts with the pathologic lung by increasing the **time- and pressure-dependence associated with** surfactant dysfunction, prolonging alveolar opening, and making alveoli more susceptible to rapid collapse. During inspiration, the mechanical ventilator forces airflow into collapsed or edematous small airways, inducing excessive **'peeling' stresses** as these airways reopen. At the same time, regional alveolar collapse and instability amplify **stress multipliers** in adjacent open alveoli, worsening RACE and OD.

These repetitive mechanical stresses lead to progressive damage to cells **and connective tissues**, with energy dissipation during each ventilatory cycle serving as a central mechanism of injury. This damage further triggers the **permeability-originated obstruction response (POOR)**, propagating injury throughout the lung, resulting in a **progressive loss of end-expiratory lung volume (EELV)** and exacerbating **surfactant dysfunction** and **endothelial permeability**. These self-perpetuating **POOR get POORer** mechanisms create a vicious cycle that accelerates the progression of VILI and lung tissue injury.

mortality [20]. In addition, lung stretch during ventilation is essential for exogenous surfactant release [21]. These factors all hinder lung reopening, which is crucial for successful weaning from the ventilator.

Another issue with the baby lung concept is that recent evidence indicates it is very difficult to cause overdistension injury in a homogeneously ventilated healthy lung [22-25]. Athletes, for instance, can generate large $V_T$ without causing stress failure [26], whereas much less strain can damage the heterogeneously ventilated ARDS lung. The risks of overinflation in the ARDS lung must stem from something other than simply stretching normal parenchymal tissue (i.e. Baby Lung). The additional factor appears to be the proximity of normal and collapsed parenchyma, which creates vulnerable

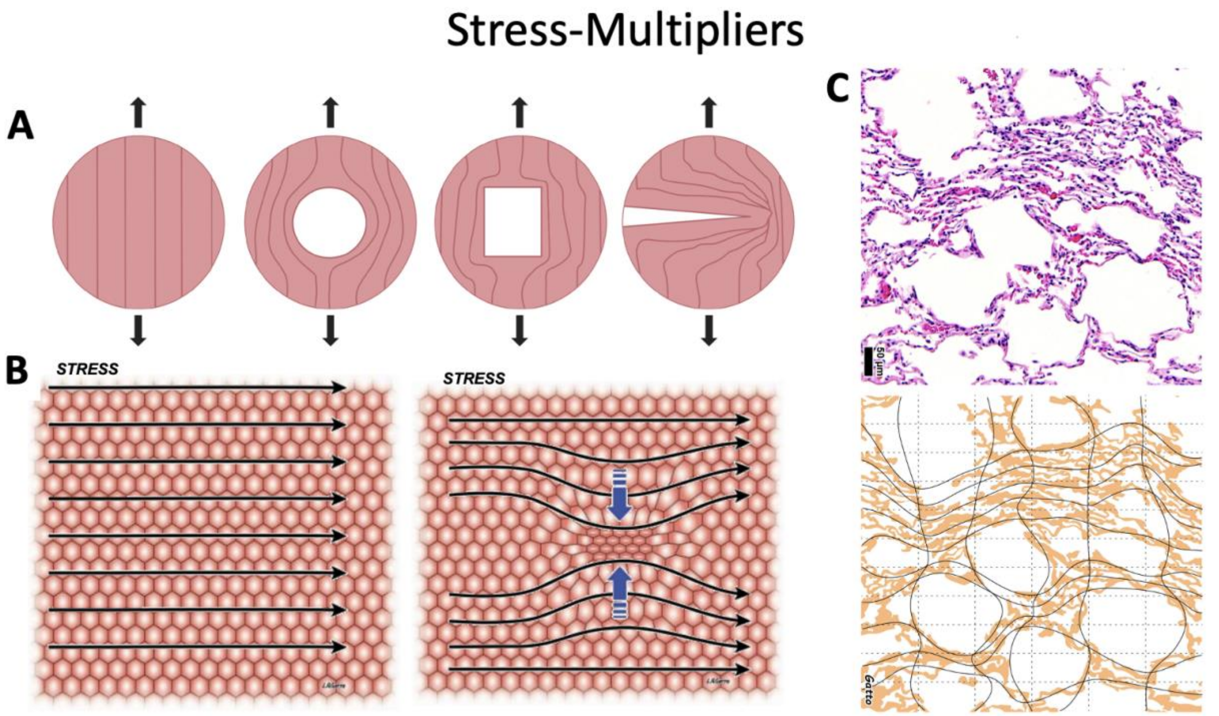


**Figure S2**. **A**) The concept of stress multiplication in a round board is examined. Force is applied at each end of the board (arrows) until the point of stress failure is reached, at which point the board fails. In the first board without defects (stress multipliers), the lines of force are equally spaced and parallel, requiring a significant force to break the board. However, defects in the board concentrate the lines of force; thus, the board with the wedge defect has the highest stress concentration (lines of force close together) and is likely to fail under a small applied force. **B**) Stress multipliers occur in lung tissue due to regional alveolar flooding or collapse. Alveoli are depicted as hexagons because they share walls with adjacent alveoli; under homogeneous inflation, the lines of force are evenly distributed and parallel. A group of collapsed alveoli causes the lines of force to concentrate toward the stress multiplier (blue arrows), overdistending the adjacent open alveoli. **C**) Acute lung injury histology shows areas of regional collapse (Hidden Micro-atelectasis) with stress lines superimposed, identifying the areas exposed to excessively high levels of stress and strain due to stress multiplication.

interfaces where alveolar wall stress is amplified by the collapsed regions' inability to contribute to overall lung expansion (Fig. S2). [5, 27-29] Stress amplification occurs because the open alveoli share walls with the collapsed alveoli, so the local expansion of the parenchyma must be accommodated entirely by the open alveoli, causing them to over-distend (Fig S2, Fig 3IC).

Repetitive alveolar collapse and expansion (RACE) damage lung tissue by two mechanisms: 1) excessive shear or 'peeling' strain as collapse alveolar walls that are in contact with each other peel open during recruitment, and 2) alveoli adjacent to these unstable alveoli are overdistended because alveolar walls are interconnected (Fig. S3). [1] All alveoli (hexagons) are open during ventilation during inspiration (Fig. S3A), in which case all alveolar walls are subject to the same stress. In contrast, when a vertical column of alveoli collapses during exhalation (Fig. S3B), the adjacent alveoli become stretched laterally, causing a concentration of horizontal stress in their walls (Fig. S3B, arrows). This

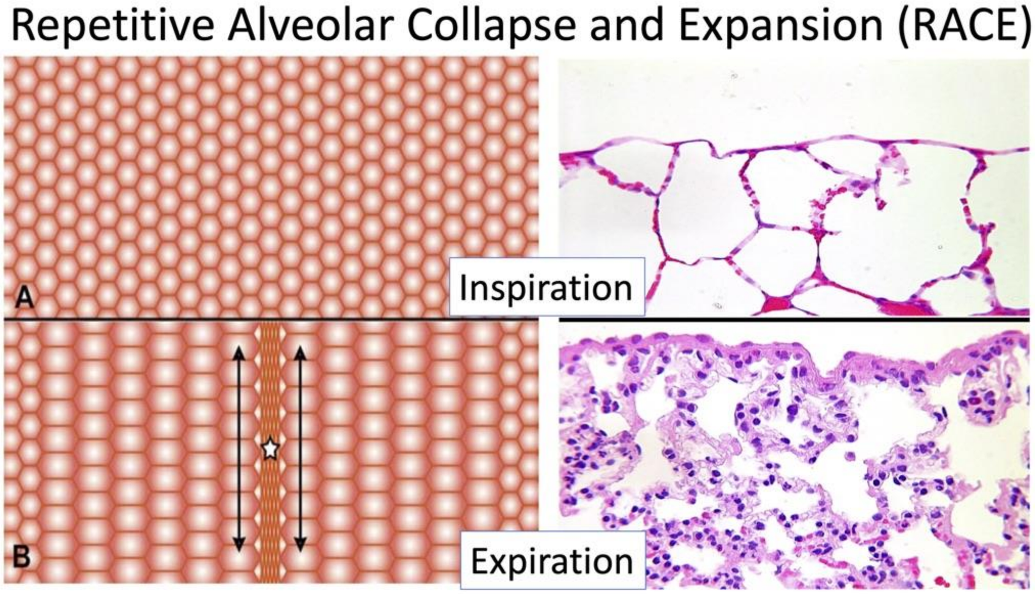


**Figure S3**. Repetitive alveolar collapse and expansion (RACE) is a dynamic process in which alveoli open during inspiration and collapse during expiration. Alveoli are represented by hexagons with shared walls. **A**) All hexagonal 'alveoli' are open at inspiration, resulting in heterogeneous ventilation, and histologically, alveoli are open with thin walls. **B**) During exhalation, some hexagonal 'alveoli' collapse (indicated by the star between double arrows), leading to the crumpling of alveolar walls, like a paper bag, and seen as thickened walls histologically. This heterogeneous ventilation is a stress multiplier, with hexagonal 'alveoli' adjacent to the collapse becoming over-distended. Thus, RACE causes VILI by two mechanisms: 1) energy dissipation secondary to shear or peeling stress as alveolar walls in apposition are pulled apart during inspiration, and 2) overdistension of the open and stable alveoli adjacent to the collapse.

stress is further increased during inspiration because collapsed alveoli cannot contribute to lung expansion. Such lines of concentrated stress are apparent in parenchymal lung tissue samples from an ARDS animal model (Fig. S2C). [1] Since VILI is invariably spatially heterogeneous, regions of stress concentration can arise throughout the parenchyma in an injured lung, leading to widely dispersed micro-scale volutrauma (Fig S2C, S3) caused by alveolar duct overdistension (Fig 3IC) [1, 30] and atelectrauma [31] (Fig S3). Furthermore, alveolar walls are shared and interconnected by a continuous web of connective tissue beginning at the alveolar mouth and running through the alveolar walls and lobules all the way to the pleural surface. This allows stress concentrations to be transmitted between inflated alveoli (Fig. S2, S3). [1]

Computational modeling of interconnected alveoli shows that stresses induced by mechanical ventilation in normal tissue adjacent to collapse can be up to three times the average across the parenchyma. Individual cell walls may experience stresses up to 16 times that measured at the ventilator. [32] A particularly concerning aspect of this micro-level stress concentration is that, even when the lung is subjected to safe levels of macro-strain and appears normal on a chest X-ray, micro-atelectatic regions that are not visible may still be present. [32-34] Studies using hyperpolarized gas and magnetic resonance imaging have identified hyperinflated areas near micro-atelectasis that can be normalized with recruitment maneuvers such as deep lung inflations [28, 35] or PEEP [36].

Volutrauma is not the only cause of VILI. Another major factor is repetitive alveolar collapse and expansion (RACE)-induced atelectrauma, which occurs when alveoli and small airways collapse or become edematous, separating their walls with fluid instead of air. Reopening these closed structures requires pressurizing the fluid layer. Resistance to this reopening arises from surface tension at the fluid-air interface, making the reopening pressure high when surfactant function is impaired. This pressure puts considerable stress on the epithelial cells as the meniscus moves over them, potentially causing rapid cell injury and death (Fig. S1). [37] The model in Figure S3B shows that micro-atelectatic regions within normal lung tissue can cause atelectrauma from RACE when closed alveoli are temporarily forced open during inspiration.

*The Role of Mechanical Power*

Multiple studies have investigated the hypothesis that VILI is associated with the mechanical power delivered to the lung by the ventilator. Viewing the respiratory system as a single alveolar compartment with elastance supplied by one airway, the equation for this is:

$$MP = f\left\{{V_T}^2\left[\frac{E_{rs}}{2} + \frac{f(1+\alpha)}{60\alpha}R_{rs}\right] + \Delta V P_0\right\} \qquad \text{Eq. 1}$$

where $f$ is the respiratory rate in breaths/min, $\alpha$ is the ratio of inspiratory to expiratory duration, $E_{rs}$ is respiratory system elastance, $R_{rs}$ is respiratory system resistance, and $P_0$ is the level of applied PEEP. [38] Numerous studies have shown that high MP levels correlate with worse outcomes [39-42].

However, it has also been shown that driving pressure (∆P) and respiratory rate are as effective as MP at predicting outcomes and are much easier to determine at the bedside. [38] ∆P is essentially $V_T$ normalized to respiratory system compliance ($C_{RS}$), which strongly correlates with increased mortality. [42] Numerous studies have shown that ∆P ($V_T/C_{RS}$) is a significantly better predictor of injurious ventilation than $V_T$ alone. [43-45]

Others have questioned the validity of MP alone as the primary driver of VILI. [43] Gattarello et al. [44] found no significant difference in lung injury between high and low MP in a 48-hour porcine model. Coppola et al. [45] demonstrated, in a retrospective analysis, no causal link between MP and mortality; however, mortality correlated with when normalized to $C_{RS}$ or EELV, underscoring the importance of lung pathophysiology in the development of VILI. Danti et al. [46] demonstrated that MP did not provide better information regarding the risk of death from VILI compared to $V_T$ size or ΔP in a meta-regression involving adults with ARDS. A pooled database of ARDS patients from six

randomized trials of lung protective ventilation and a large observational cohort found that higher MP was associated with increased mortality but was not superior to respiratory rate (RR) or ΔP. [39]

The current clinical approach is to adjust RR, inspiratory/expiratory ratio, PEEP, and $V_T$ to minimize MP (Eq. 1). This approach is based on the concept of a ‘safe region’ in the $V_T$-PEEP plane where no lung damage occurs, but this region becomes progressively smaller as lung damage increases in severity, making it more difficult to ventilate the lung safely. [23] Instead of adjusting ventilator settings to reduce MP, a better solution would be to address the underlying pathophysiologic problem, decrease $E_{rs}$, and normalize EELV. This can be achieved by stabilizing

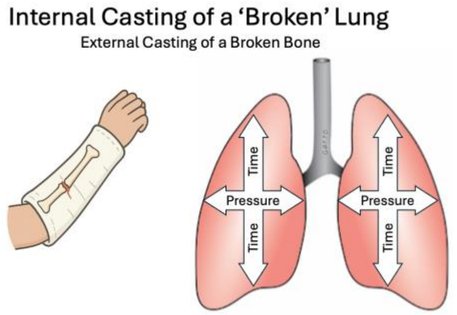


**Figure S4**. The goal of any protective ventilation strategy should be to apply an internal ‘cast’ using a precise combination of airway pressure and time, similar to the external case applied to a broken bone. This is necessary because a heterogeneous distribution of RACE and stress multipliers is the mechanism of VILI in the microenvironment. The APRV mode, set and adjusted using the TCAV method, has been shown to ‘cast’ open alveoli (Fig. 5) and the entire lung (Fig. 4), resulting in homogeneous ventilation free of RACE and stress multipliers. TCAV settings and adjustments were similar to those recommended in our Guidelines.

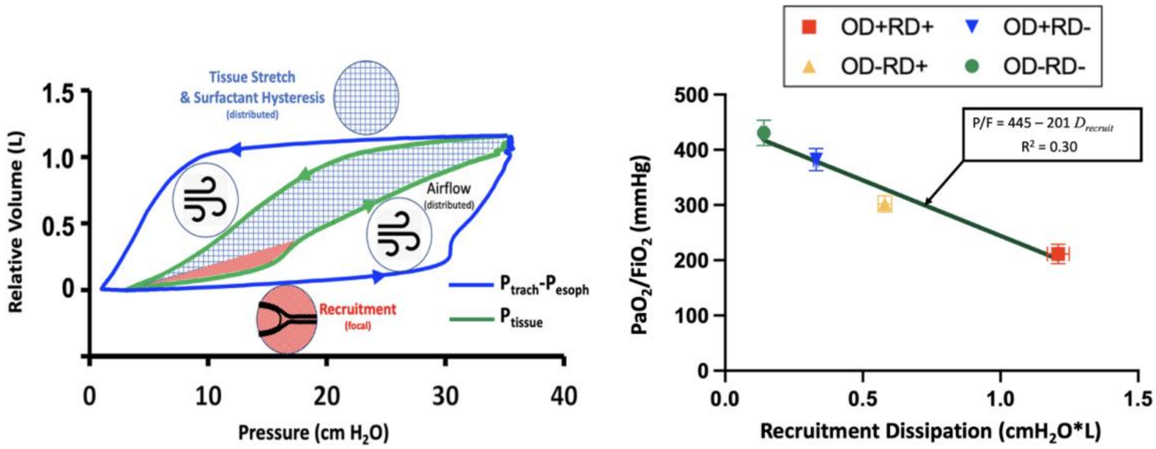


**Figure S5**. Identification that localized and rapid alveolar recruitment results in energy dissipation correlated with lung pathophysiology. **A**) The hysteresis in the whole lung pressure/volume curve is a measure of energy dissipated into the lung with each breath. The two largest energy dissipation mechanisms are Airflow distribution (resistance) measured by the transpulmonary pressure (Ptracheal – Pesophageal, blue line) and lung tissue and surfactant hysteresis (green line). This occurs with every breath and is not harmful. Using data from a porcine heterogeneous lung injury model (Fig. 4), we found that energy dissipation is highly localized and rapid during alveolar recruitment (red). **B**) More importantly, the group that causes the most energy dissipation OD+RD+ (red) causes the most pathophysiology (fall in $PaO_2/FiO_2$), and the group with the least dissipated energy OD-RD- (green) caused negligible injury. [4] OD-RD- group settings and adjustments were similar to those recommended in our Guidelines.

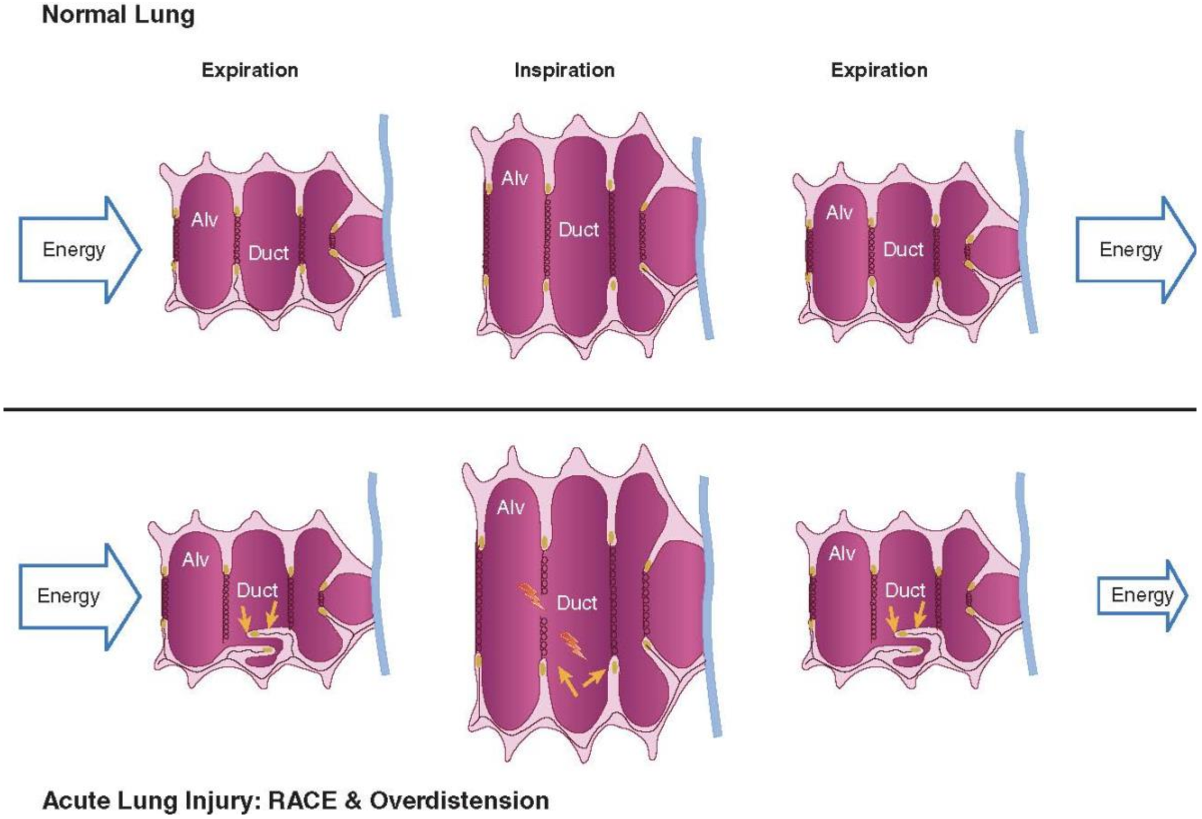


**Figure S6**. High mechanical power or energy delivered to the lungs has been associated with poor outcomes in patients with ARDS. However, it is not the energy *delivered* to the lungs that causes lung damage; rather, the energy is *dissipated* and does not return during exhalation. In a normal lung**,** the energy delivered to the acinus stretches and expands the alveolar ducts by enlarging the elastin (springs connecting alveolar walls) that make up the alveolar mouths. During exhalation, elastin recoils to its resting position, returning all energy, resulting in no tissue damage (Normal Lung; Energy In and Out arrows of equal size). Energy is dissipated by resistance to gas flow in the airways, by tissue viscoelasticity, and by surfactant function; this is normal and does not cause injury (Fig. S4). *Acute lung injury* impairs pulmonary surfactant function and alters alveolar mechanics, leading to repetitive alveolar collapse and expansion (RACE) (Fig. 5, S3) and alveolar duct overdistension (Fig. 5). Collapsed alveolar walls in apposition (gold arrows) occur during *expiration*. During *inspiration,* energy is dissipated (as a lightning bolt) on the alveolar walls as they peel open, causing shear-stress injury. In addition, stress multipliers contribute to overdistension, leading to tissue tears (lightning bolts on elastin springs). Alveoli re-collapse during exhalation, and the process repeats with each breath.

the lung open internally, using appropriate airway pressures and timing—similar to stabilizing a broken arm with an external cast (Fig. S4) [1, 46], which helps reduce and expand the safe region in the $V_T$-PEEP plane. [23]

Importantly, even if MP is high (Eq. 1), it causes no tissue damage provided that all energy delivered to the tissues during inspiration is returned to the environment during expiration. This occurs if tissue stresses are purely elastic. Damage can only result from energy that is irreversibly dissipated within the tissues.[43] However, it is perfectly normal

to have energy dissipation in the lung due to gas flow along the airways, stretching of viscoelastic parenchyma, and tension-area hysteresis at the air-liquid interface. Instead, the energy dissipated by lung recruitment during inspiration is what correlates with lung injury (Figs. S5, S6). [4]

*The Physiologic Rationale for our Guideline Recommendations*

Lung volume changes as a viscoelastic system, with a delay between the application of airway pressure and the lung's full inflation (Fig. S7A). If inspiratory airway pressure is maintained, the lung will continue to 'creep' open over time without an increase in airway pressure. This allows the lung to be inflated gradually without increasing driving pressure or mechanical power. During deflation, there is a lag between the decrease in airway pressure and the onset of lung tissue collapse (Fig. S7B). Mechanical ventilation strategies can use this knowledge to promote recruitment and prevent re-collapse by adjusting inspiratory and expiratory times.

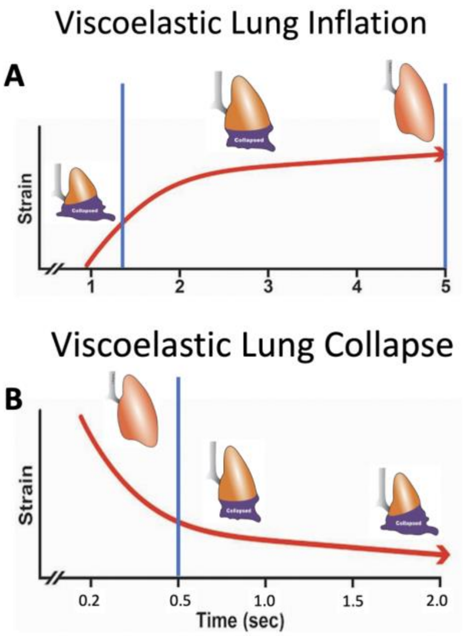


**Figure S7**. Viscoelastic lung inflation (A) and collapse (B) are shown. Once airway inflation pressure is applied, the lung initially opens rapidly; however, if the pressure is maintained, it continues to 'creep' open without a change in pressure (A). Once airway pressure is reduced, there is a brief delay before the lung begins to collapse, which then continues until the lung is reinflated during inspiration. If inspiratory time is extended to the right of the blue line (A) and expiratory time set to stop and reinflate the lung to the left of the blue line (B), the lung will be slowly ratcheted up one breath at a time.

Loss of functional surfactant secondary to ARDS alters the viscoelastic dynamics of recruitment and derecruitment, such that, compared with a normal lung, derecruitment occurs more quickly and at higher expiratory pressures, whereas recruitment takes longer and requires higher inspiratory pressures. This pathophysiology suggests that increasing the duration of inspiration can help recruit alveolar tissue in the injured lung, whereas reducing expiration can prevent re-collapse. Therefore, properly adjusting the timing of the breath cycle could reduce the repetitive

alveolar collapse and expansion (RACE) and stress multipliers, which are the main causes of atelectrauma (Figs. 5, S2, S3). [4]

Evidence for this has been demonstrated in a porcine model of lung injury induced by heterogeneous surfactant dysfunction. In this study, four groups of pigs were mechanically ventilated with airway pressure release ventilation (APRV) to separately manipulate the degree of overdistension (OD) and RACE (recruitment/derecruitment - RD) (Fig. S8). In the lung protective, or OD↓RD↓ group, inspiratory pressure was moderate (28 $cmH_2O$), and expiratory duration was kept short using the time-controlled

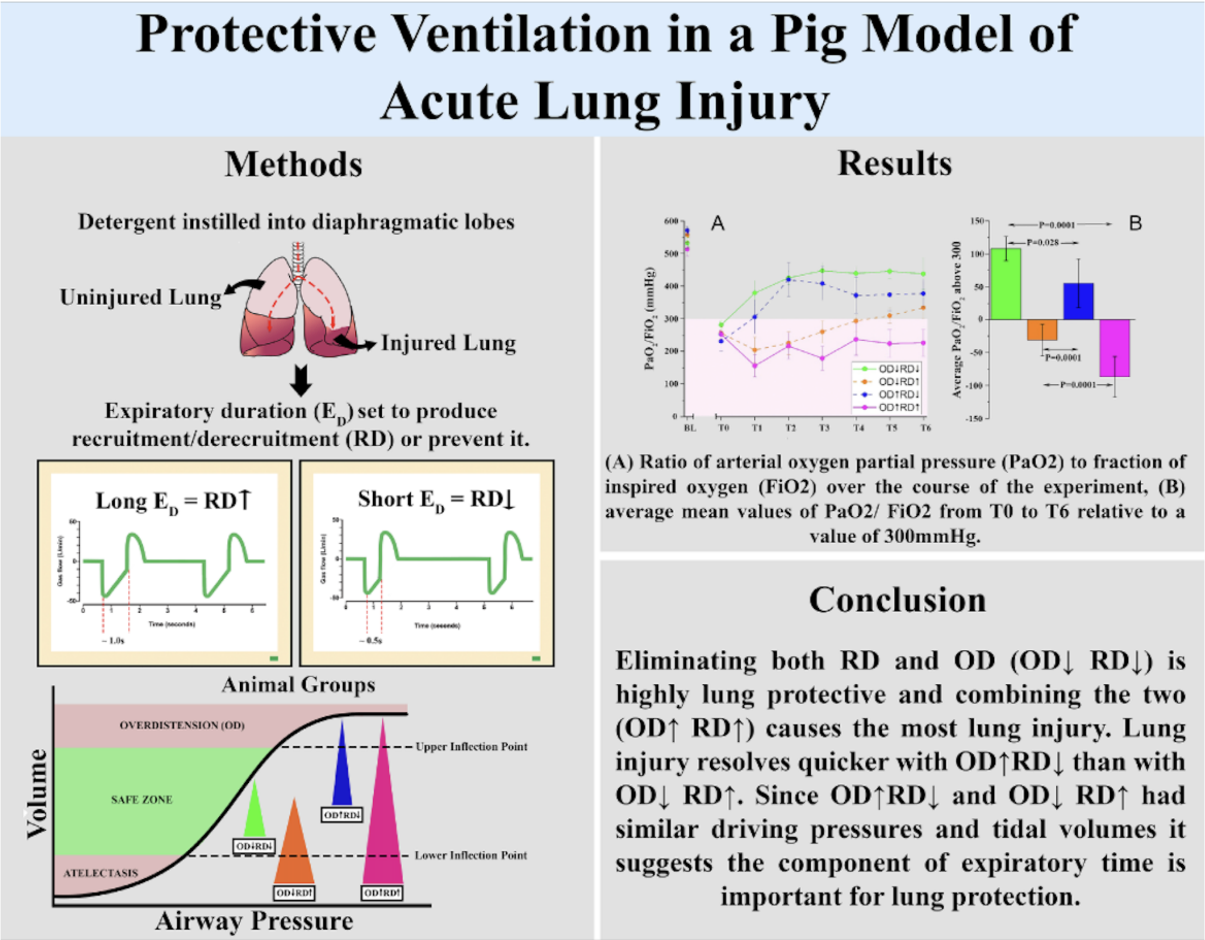


**Figure S8**. A graphic abstract of a study investigating the role of overdistension (OD) and alveolar recruitment/derecruitment (RD) in VILI pathogenesis. A porcine heterogeneous lung injury model was used to measure OD and RD in both normal (baby lung) and surfactant-depleted (ARDS) tissues. The APRV mode was used in all four groups with a $P_{High}$ set at 40 $cmH_2O$ to cause OD↑ and 28 $cmH_2O$ to prevent OD↓. A long expiratory time was set to induce RD↑, while a very brief expiratory time was implemented to minimize RD↓. OD↓RD↓ (green) was the most lung-protective, with APRV settings similar to those recommended in our Guidelines. Normal lung tissue was not injured even in the OD↑RD↑ group, suggesting OD of the 'baby lung' is not the mechanism driving VILI. OD↓RD↑ caused more injury than OD↑RD↓ with similar driving pressures and tidal volumes, emphasizing the critical role of RD and expiratory time for lung protection.

adaptive ventilation (TCAV) method to set APRV, adjusting expiratory time based on peak expiratory flow ($F_{PE}$) and the slope of the expiratory flow curve, which is a breath-by-breath assessment of $C_{RS}$. In the OD↑RD↓ group, inspiratory pressure was high (40 cm$H_2O$) to cause OD, while expiratory duration was again kept short to prevent RACE (RD). In the OD↓RD↑ group, moderate inspiratory pressure was combined with an extended expiratory duration, both adjusted using $F_{PE}$. Finally, in the OD↑RD↑ group, animals were subjected to both overdistension and RACE. [25] The OD↓RD↓ group was ventilated in accordance with the recommendations outlined in our APRV Guidelines. It protected both normal and surfactant-deficient lung tissues, supporting the benefits of TCAV as a protective strategy during mechanical ventilation of the injured lung. Furthermore, the OD↓ RD↑ group had more tissue damage (VILI) than the OD↑ RD↓ group, despite having nearly identical $V_T$ and driving pressures (ΔP) (Fig. S8, Conclusions).

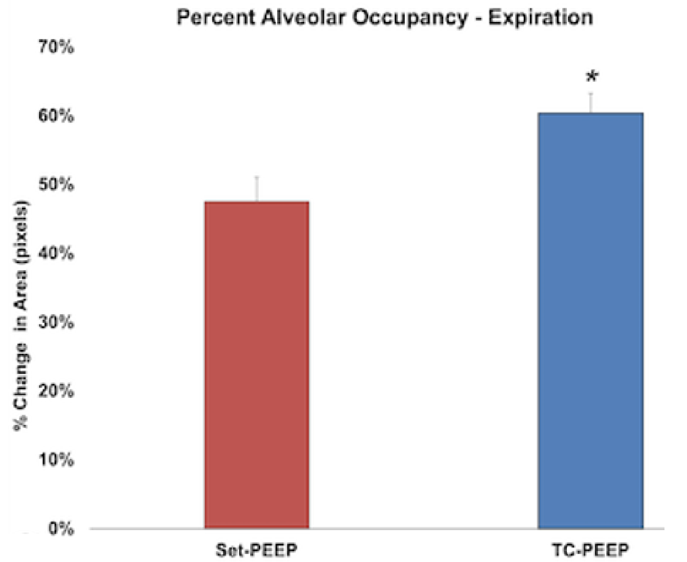


**Figure S9:** The same alveoli were examined during expiration using *in vivo* microscopy with brief and extended expiratory times at the same positive end-expiratory pressure (PEEP). The objective was to determine the influence of expiratory time and pressure on alveolar stabilization. Both groups used the airway pressure release ventilation (APRV) mode. ***TC-PEEP group***: The low pressure ($P_{Low}$) was set to 0 cm$H_2O$, and a very brief expiratory time ($T_{Low}$) was used with the time-controlled adaptive ventilation (TCAV) method to set $T_{Low}$. The short expiratory duration did not allow the lung to fully depressurize, thereby maintaining a time-controlled PEEP (TC-PEEP) (Fig. 2, red dotted line). ***Set-PEEP group***: The TC-PEEP was measured during short $T_{Low}$ and used to set the $P_{Low}$ in the Set-PEEP group. Consequently, the Set-PEEP and TC-PEEP groups had identical end-expiratory pressures, with the sole difference being that expiratory time ($T_{Low}$) was extended in the Set-PEEP group. Assessment of alveolar collapse measured in all four rats as a Percent Change in Alveolar Area (%) of all alveoli within a microscopic field. A brief expiratory time (TC-PEEP) group significantly reduced alveolar collapse (blue bar) as compared to an extended expiratory time (red bar) (* = P<0.05).

The role of expiratory time in alveolar collapse has also been studied using *in vivo* microscopy, which images subpleural alveoli and can determine the pressure-duration combination required to prevent collapse during expiration. Using our *in vivo* microscope, we set the expiratory time with APRV mode and the TCAV method. We measured TC-

PEEP (Fig. 2, red dotted line) and calculated alveolar derecruitment as the percentage of the microscope slide occupied by inflated alveoli (Fig. S9, TC-PEEP). We then set the PEEP to match the TC-PEEP, extended expiratory time, and measured alveolar derecruitment (Fig. S9, Set-PEEP). The study showed that alveolar derecruitment can be prevented at the same PEEP with a sufficiently brief expiratory time ($T_{Low}$) compared to a longer expiratory duration at the same PEEP. This demonstrates that a brief expiratory duration (TC-PEEP group) is more effective than pressure alone (Set-PEEP group) at stabilizing alveoli and preventing their collapse. [1] This is due to the viscoelastic nature of alveolar collapse (S7B)

Additionally, prolonged inspiratory durations enhance lung tissue recruitment without increasing driving pressure or dissipating mechanical energy—they are simply sustained for longer (Fig. S7A, between the blue lines). Conversely, short expiratory times prevent progressive collapse when PEEP is added (Fig. S7B, left of the blue line). Combining extended inspiratory durations ($T_{High}$) with brief expiratory durations ($T_{Low}$) in APRV helps normalize alveolar and duct sizes (Fig. 5). When applied to the ARDS lung, using the TCAV method (Fig. S8, OD↓RD↓) prevents lung collapse during expiration and promotes recruitment during the next inspiration, thereby significantly reducing energy dissipation and tissue injury (Figs S5, S6). [4] This emphasizes the importance of considering both time and pressure in maintaining uniform ventilation of alveoli and alveolar ducts while minimizing tissue strain (Fig. 5). [30]

*Summary*

The increase in endothelial permeability driven by inflammation is the initial pathologic process that leads to surfactant dysfunction and pulmonary edema (Fig S1). This results in uneven ventilation and altered alveolar mechanics, which contribute to ongoing lung damage. Currently, no drugs are available to prevent this rise in endothelial permeability [47, 48], so VILI remains a constant risk. When surfactant function is compromised, alveoli open more slowly and collapse more rapidly at any given airway pressure, leading to regional alveolar collapse that worsens over time. The loss of EELV associated with this causes hypoxemia and necessitates mechanical ventilation, which must be managed to maintain lung volume and prevent RACE and regional alveolar

overdistension at the boundary between normal and collapsed alveoli (Fig. S2, S3). In the APRV mode, when configured using the TCAV method, the ventilation achieves these goals (Figs. 4, 5, 7). As a result, APRV could break the VILI Vortex [6] and reduce the incidence and mortality of ARDS (Fig. 10). [49] Therefore, it is crucial to determine the optimal method for setting and personalizing APRV for each patient. [1] Our current guidelines aim to provide clinicians with the tools needed to implement the TCAV method of APRV in patients with ARDS.

## References


1. Nieman, G.F., N.M. Habashi, and SpringerLink, *Applied Physiology to Reduce Ventilator Induced Lung Injury : Clinical Applications for the Acutely Injured Lung*. 1st 2024. ed. 2024, Cham: Springer Nature Switzerland : Imprint: Springer.
2. Bellani, G., et al., *Epidemiology, Patterns of Care, and Mortality for Patients With Acute Respiratory Distress Syndrome in Intensive Care Units in 50 Countries.* JAMA, 2016. **315**(8): p. 788-800.
3. Nieman, G.F., et al., *The role of pulmonary surfactant on lung fluid balance.* Am J Physiol Lung Cell Mol Physiol, 2025. **329**(2): p. L307-L314.
4. Gaver, D.P., 3rd, et al., *Mechanical ventilation energy analysis: Recruitment focuses injurious power in the ventilated lung.* Proc Natl Acad Sci U S A, 2025. **122**(10): p. e2419374122.
5. Gaver, D.P., 3rd, et al., *The POOR Get POORer: A Hypothesis for the Pathogenesis of Ventilator-induced Lung Injury.* Am J Respir Crit Care Med, 2020. **202**(8): p. 1081-1087.
6. Marini, J.J. and L. Gattinoni, *Time Course of Evolving Ventilator-Induced Lung Injury: The "Shrinking Baby Lung".* Crit Care Med, 2020. **48**(8): p. 1203-1209.
7. Gattinoni, L. and A. Pesenti, *The concept of "baby lung".* Intensive Care Med, 2005. **31**(6): p. 776-84.
8. Li Bassi, G., et al., *Short-Term Appraisal of the Effects and Safety of Manual Versus Ventilator Hyperinflation in an Animal Model of Severe Pneumonia.* Respir Care, 2019. **64**(7): p. 760-770.
9. Lutz, D., et al., *Alveolar derecruitment and collapse induration as crucial mechanisms in lung injury and fibrosis.* Am J Respir Cell Mol Biol, 2015. **52**(2): p. 232-43.
10. Burkhardt, A., *Alveolitis and collapse in the pathogenesis of pulmonary fibrosis.* Am Rev Respir Dis, 1989. **140**(2): p. 513-24.
11. Cabrera-Benitez, N.E., et al., *Mechanical ventilation-associated lung fibrosis in acute respiratory distress syndrome: a significant contributor to poor outcome.* Anesthesiology, 2014. **121**(1): p. 189-98.
12. Duggan, M., et al., *Atelectasis causes vascular leak and lethal right ventricular failure in uninjured rat lungs.* Am J Respir Crit Care Med, 2003. **167**(12): p. 1633-40.
13. Whittenberger, J.L., et al., *Influence of state of inflation of the lung on pulmonary vascular resistance.* J Appl Physiol, 1960. **15**: p. 878-82.
14. Pearse, D.B., et al., *Effects of tidal volume and respiratory frequency on lung lymph flow.* J Appl Physiol (1985), 2005. **99**(2): p. 556-63.
15. Mellott, K.G., et al., *Patient-ventilator dyssynchrony: clinical significance and implications for practice.* Crit Care Nurse, 2009. **29**(6): p. 41-55 quiz 1 p following 55.
16. Burki, N.K. and L.Y. Lee, *Mechanisms of dyspnea.* Chest, 2010. **138**(5): p. 1196-201.

17. Solomon, I.C., N.H. Edelman, and J.A. Neubauer, *Pre-Botzinger complex functions as a central hypoxia chemosensor for respiration in vivo.* J Neurophysiol, 2000. **83**(5): p. 2854-68.
18. Widdicombe, J., *Airway receptors.* Respir Physiol, 2001. **125**(1-2): p. 3-15.
19. Yu, J., *Deflation-activated receptors, not classical inflation-activated receptors, mediate the Hering-Breuer deflation reflex.* J Appl Physiol (1985), 2016. **121**(5): p. 1041-1046.
20. Blanch, L., et al., *Asynchronies during mechanical ventilation are associated with mortality.* Intensive Care Med, 2015. **41**(4): p. 633-41.
21. Majumdar, A., et al., *Jamming dynamics of stretch-induced surfactant release by alveolar type II cells.* J Appl Physiol (1985), 2012. **112**(5): p. 824-31.
22. Schreiber, T., et al., *PEEP has beneficial effects on inflammation in the injured and no deleterious effects on the noninjured lung after unilateral lung acid instillation.* Intensive Care Med, 2006. **32**(5): p. 740-9.
23. Protti, A., et al., *Lung stress and strain during mechanical ventilation: any difference between statics and dynamics?* Crit Care Med, 2013. **41**(4): p. 1046-55.
24. Seah, A.S., et al., *Quantifying the roles of tidal volume and PEEP in the pathogenesis of ventilator-induced lung injury.* Ann Biomed Eng, 2011. **39**(5): p. 1505-16.
25. Ramcharran, H., et al., *Protective ventilation in a pig model of acute lung injury: timing is as important as pressure.* J Appl Physiol (1985), 2022. **133**(5): p. 1093-1105.
26. Blackie, S.P., et al., *Normal values and ranges for ventilation and breathing pattern at maximal exercise.* Chest, 1991. **100**(1): p. 136-42.
27. Albert, K., et al., *Hidden Microatelectases Increase Vulnerability to Ventilation-Induced Lung Injury.* Front Physiol, 2020. **11**: p. 530485.
28. Cereda, M., et al., *Positive end-expiratory pressure increments during anesthesia in normal lung result in hysteresis and greater numbers of smaller aerated airspaces.* Anesthesiology, 2013. **119**(6): p. 1402-9.
29. Retamal, J., et al., *Non-lobar atelectasis generates inflammation and structural alveolar injury in the surrounding healthy tissue during mechanical ventilation.* Crit Care, 2014. **18**(5): p. 505.
30. Kollisch-Singule, M., et al., *Airway pressure release ventilation reduces conducting airway micro-strain in lung injury.* J Am Coll Surg, 2014. **219**(5): p. 968-76.
31. Kollisch-Singule, M., et al., *Mechanical breath profile of airway pressure release ventilation: the effect on alveolar recruitment and microstrain in acute lung injury.* JAMA Surg, 2014. **149**(11): p. 1138-45.
32. Makiyama, A.M., et al., *Stress concentration around an atelectatic region: a finite element model.* Respir Physiol Neurobiol, 2014. **201**: p. 101-10.
33. Grasso, S., et al., *Inhomogeneity of lung parenchyma during the open lung strategy: a computed tomography scan study.* Am J Respir Crit Care Med, 2009. **180**(5): p. 415-23.
34. Mead, J., T. Takishima, and D. Leith, *Stress distribution in lungs: a model of pulmonary elasticity.* J Appl Physiol, 1970. **28**(5): p. 596-608.

35. Cereda, M., et al., *Quantitative imaging of alveolar recruitment with hyperpolarized gas MRI during mechanical ventilation.* J Appl Physiol (1985), 2011. **110**(2): p. 499-511.
36. Cereda, M., et al., *Imaging the interaction of atelectasis and overdistension in surfactant-depleted lungs.* Crit Care Med, 2013. **41**(2): p. 527-35.
37. Bilek, A.M., K.C. Dee, and D.P. Gaver, 3rd, *Mechanisms of surface-tension-induced epithelial cell damage in a model of pulmonary airway reopening.* J Appl Physiol (1985), 2003. **94**(2): p. 770-83.
38. Gattinoni, L., et al., *Ventilator-related causes of lung injury: the mechanical power.* Intensive Care Med, 2016. **42**(10): p. 1567-1575.
39. Costa, E.L.V., et al., *Ventilatory Variables and Mechanical Power in Patients with Acute Respiratory Distress Syndrome.* Am J Respir Crit Care Med, 2021. **204**(3): p. 303-311.
40. Marini, J.J., P.R.M. Rocco, and L. Gattinoni, *Static and Dynamic Contributors to Ventilator-induced Lung Injury in Clinical Practice. Pressure, Energy, and Power.* Am J Respir Crit Care Med, 2020. **201**(7): p. 767-774.
41. Cressoni, M., et al., *Mechanical Power and Development of Ventilator-induced Lung Injury.* Anesthesiology, 2016. **124**(5): p. 1100-8.
42. Tonetti, T., et al., *Driving pressure and mechanical power: new targets for VILI prevention.* Ann Transl Med, 2017. **5**(14): p. 286.
43. Bates, J.H.T., et al., *Mechanical Power and Ventilator-induced Lung Injury: What Does Physics Have to Say?* Am J Respir Crit Care Med, 2024. **209**(7): p. 787-788.
44. Gattarello, S., et al., *Impact of Fluid Balance on the Development of Lung Injury.* Am J Respir Crit Care Med, 2025. **211**(3): p. 331-338.
45. Coppola, S., et al., *Effect of mechanical power on intensive care mortality in ARDS patients.* Crit Care, 2020. **24**(1): p. 246.
46. Dianti, J., et al., *Comparing the Effects of Tidal Volume, Driving Pressure, and Mechanical Power on Mortality in Trials of Lung-Protective Mechanical Ventilation.* Respir Care, 2021. **66**(2): p. 221-227.
47. Fan, E., D. Brodie, and A.S. Slutsky, *Acute Respiratory Distress Syndrome: Advances in Diagnosis and Treatment.* JAMA, 2018. **319**(7): p. 698-710.
48. Qadir, N., et al., *An Update on Management of Adult Patients with Acute Respiratory Distress Syndrome: An Official American Thoracic Society Clinical Practice Guideline.* Am J Respir Crit Care Med, 2024. **209**(1): p. 24-36.
49. Andrews, P.L., et al., *Early application of airway pressure release ventilation may reduce mortality in high-risk trauma patients: a systematic review of observational trauma ARDS literature.* J Trauma Acute Care Surg, 2013. **75**(4): p. 635-41.
