# Supplementary material for: Expert recommendations for setting and adjusting airway pressure release ventilation based on clinical experience and basic science evidence
Source: Front Med (Lausanne). 2026 Feb 3;13:1741129. doi: 10.3389/fmed.2026.1741129 (PMC12909506; doi:10.3389/fmed.2026.1741129)
Supplement: Supplementary file 4 [file Supplementary_file_4.pdf]

Supplementary File 4: Adult human trials using airway pressure release ventilation (APRV)

Trial	Year	Settings P_{High}	T_{High}	P_{Low}	T_{Low}	Principal Findings and Score
PPV vs. APRV post-surgery on 14 Patients for 30 m in. Garner, DOI: 10.1378/chest.94.4.779	1988	1;12	NL	NL	4;1.5	APRV supported oxygenation and ventilation in patients with mild acute lung injury similar to PPV but with a lower peak airway pressure. Score: B – Oxygenation and ventilation
APRV vs. CV ARDS PT on 50 patients for 30 min. Rasanen, DOI: 10.1097/00003246-199110000-00004,	1991	2;21±9	NL	1;6±5	4;1.5	APRV is a feasible alternative to CV for patients with moderate to mild ALI producing similar oxygenation and ventilation at significantly lower Ppeak. Score: B – Oxygenation and ventilation; C - Ppeak
APRV vs. IV-IRA PT 24hrs on 18 ALI patients. Sydow, DOI: 10.1164/ajrccm.149.6.8004312	1994	3;15-30	1;2-4	1;5	1;0.5-0.7	Similar hemodynamics. APRV reduced shunt significantly after 8 hrs, suggesting recruitment. Ppeak lower. Score: C – Oxygenation; B- Hemodynamics
Crossover PC-IRA to APRV Number of patients not listed Kaplan, DOI: 10.1186/cc1027	2001	1;25±3	1;4.5	1;0	1;0.8	APRV significantly reduced Ppeak, Pmean, CVP, lactate, paralytic, pressors, and sedative use and increased CO, DO_2, and UO. Score: C – for all of the above parameters
APRV with and without SB on 30 patients for 72hrs Putensen, DOI: 10.1164/ajrccm.164.1.2001078	2001	NL	NL	NL	NL	APRV with SB increased C_{RS}, PaO_2, CO, and DO_2 and decreased Qs/Qt, sedation, pressors, Ppeak, with shorter VD. Score: C – for all of the above parameters.
APRV vs. PS-SIMV ARDS on 59 patients for the hospital stay. Varpula, doi: 10.1111/j.0001-5172.2004.00411.x	2004	5;<35	1;4	2:10	1;1	APRV did not differ from PS-SIMV in PEEP, PaO_2/FiO_2, arterial pH, MAP, CO, MV, with a significant decrease in Ppeak. Score: B – for all parameters except Ppeak – C.
APRV vs. PS, CT ARDS SB RT on 18 patients for the hospital stay. Yoshida, DOI: 10.1213/ANE.0b013e3181bbd918	2009	1;<30	1;4	3;0	3;NL	APRV increased aerated lung and reduced atelectasis improving P/F. Score: C – for all of the above parameters.
APRV vs LV_T Trauma MV 72hrs on 63 patients for the hospital stay. Maxwell, DOI: 10.1097/TA.0b013e3181e75961		4;23	2;NL	NL	4;NL	No differences in VD, ICU LOS, VAP, Ppeak pneumothorax, PO_2, pH, PCO_2, or mortality. Score: B – for all of the above parameters.
Early APRV vs. LV_T ARDS on 138 patients for the hospital stay. Zhou, DOI 10.1007/s00134-017-4912-z	2017	4;24	1;NL	3;0	3;NL	APRV improved P/F and C_{RS} with Less sedation. APRV increased ventilator-free days, ICU stay, and trend for mortality. Score: C – for all the above parameters.
APRV-LV_T vs. APRV vs. LV_T on 52 patients for 3 days. Hirshberg, DOI: 10.1097/CCM.0000000000003437	2018	3;NL	NL	4;NL	NL	No differences in P/F, sedation, pressors, or pneumothorax. High V_T Score: B – for all of the above parameters.
APRV vs. LV_T RCT CARDS On 55 patients for 28 days. Ibarra-Estrada, DOI: 10.1097/CCM.0000000000005312	2021	4;NL	1;4	NL	3;NL	APRV had higher P/F and C_{RS} but no difference in VFD or mortality. Score: C – P/F, C_{RS}; B – VFD, mortality

APRV vs SIMV PT CARDS on 12 patients for 21 days<br>Joseph, doi.org/10.36401/ISIM-20-03	2021	4;NL	1;5	3;0	2;NL	APRV increased P/F and reduced FiO_2, pressors, and sedation.<br>Score: C - for all of the above parameters.
APRV vs. PCV OR RCT On 39 patients for 3 days.<br>Ge, doi: 10.3389/fphys.2021.684927	2021	4;NL	1;3.6-6.0	3;0	2;NL	APRV improved CO, P/F, C_{RS}, & Xray ICU LOS and VD were similar.<br>Score: C – CO, PF; B – ICU LOS, VD
Eary APRV vs. PS-SIMV on 65 patients for 3 days.<br>Kucuk, doi.org/10.1016/j.bjane.2021.03.022	2021	3;NL	1;4	3;0	6;NL	APRV improved P/F, ICU LOS & reduced FiO_2. Similar sedation, ARDS incidence, & mortality.<br>Score: C – P/F, LOS, FiO_2; B – sedation, incidence, mortality.
APRV ARDS EIT PT On 12 patients for 24hrs<br>Li, doi.org/10.1186/s13054-023-04469-8	2023	4;NL	NL	3;0	3;NL	24hrs on APRV optimized V/Q, reduced LH Improved PO_2, P/F, C_{RS}, $PaCO_2$,<br>Score: C – V/Q, LH,
APRV vs. LV_T RCT EIT on 40 patients for 24hrs.<br>Zou, doi.org/10.1016/j.chest.2024.08.050	2025	4;NL	1;NL	3;0	2;0.4	APRV improved V/Q & LH, PO_2, FiO_2, C_{RS}, & PCO_2. No difference in CO or DS.<br>Score: C – all parameters except CO & DS - B

Numerical code for the 4 APRV settings: The first # are the setting method codes below (1-6). The second # is the actual value in cmH_2O (P_{High}, P_{Low}) or seconds (T_{High}, T_{Low}).

Settings methods numbering system:
P_{High} **-** 1) arbitrarily set in cmH_2O, 2) set to optimize PaO_2/FiO_2 with cardiovascular compromise, 3) set based on tidal volume (V_T), 4) set based on conventional ventilation peak or plateau airway pressure, or 5) titrated with PV-curve.

T_{High} **-** 1) arbitrarily set in seconds, 2) based on SB rate, or 3) adjusted to maintain $PaCO_2$.

P_{Low} **-** 1) arbitrarily set in cmH_2O, 2) titrated with PV-curve, 3) set at 0 cmH_2O, or 4) set based on tidal volume V_T.

T_{Low} **-** 1) arbitrarily set in seconds, 2) set at 75% of peak expiratory flow (P_{EF}), 3) set at 50%-75% of P_{EF}, 4) set at 25%-75% of P_{EF}, 5) adjusted to a targeted V_T, or 6) adjusted to a targeted $PaCO_2$.

NL = Settings method not listed or unclear

Example:
If the P_{High} is arbitrarily set (1) at (12) cmH_2O, it would be **1;12**. If T_{Low} were set at 50%-75% of $F_{PE,}$ resulting in a 0.5sec expiratory duration, it would be **3; 0.5**.

Principal Findings Score summary: APRV vs other ventilation groups: **A)** Negative Impact, **B)** Neutral Impact, and **C)** Positive Impact.

Abbreviations.

Measured lung mechanics parameters
High airway pressure (P_{High}); High airway pressure time (T_{High}); Low airway pressure (P_{Low}); Low airway pressure time (T_{Low}). SB = Spontaneous Breathing; Ppeak = Peak airway pressure; Pmean = Mean airway pressure; CPAP = Continuous positive airway pressure; C_{RS} = Respiratory System Compliance; MV = Minute Ventilation; LH = Lung heterogeneity (EIT).

Measured hemodynamic and blood gas exchange parameters
MAP - Mean arterial pressure; CO = Cardiac Output; CVP = Central Venous Pressure; UO = Urine Output; DO_2 = Oxygen Delivery; Qs/Qt = venous admixture, P/F = PaO_2/FiO_2 ratio, DS = Dead Space; V/Q = Ventilation/Perfusion ratio.

Ventilator modes and methods
MV = Mechanical Ventilation; PPV = Conventional positive pressure ventilation; PCV = Pressure Controlled Ventilation; CV = Conventional ventilation; VC-IRA = Volume-controlled Inverse Ratio; PC-IRA = Pressure-controlled Inverse Ratio; PS-

SIMV = Pressure Support with synchronized intermittent ventilation; LV_T = Low tidal volume ventilation, APRV-LV_T = APRV adjusted to maintain a V_T at or below 6cc/kg.

Severity of lung injury, type of clinical trial, and measured clinical parameters
ALI = Acute Lung Injury; ARDS = Acute Respiratory Distress Syndrome; PT = Prospective Trial; RT = Retrospective trial, RCT = Randomized Controlled Trial; RST = Randomized Superiority Trial CT = Computed Tomography; EIT = Electrical Impedance Tomograph; VD = Ventilator days; VFD = Ventilator free days; ICU LOS = Intensive Care Unit Length of Stay; OR = Operating Room; VAP = Ventilator-associated pneumonia; Open Lung = Lung heterogeneity (LH) improvements

Principal Findings Score summary: APRV vs other ventilation groups: **A)** Negative Impact, **B)** Neutral Impact, and **C)** Positive Impact.

Table S3

Publications	Oxygenation	Ventilation	Arterial pH	LM	Open Lung	Hemo	UO	Sedation	Paralytics	Pressors	VFD	ARDS%	Mortality	Pneumo	VAP	ICU LOS	X-Ray
Garner, DOI: 10.1378/chest.94.4.779	B	B		C													
Rasanen, DOI:10.1097/00003246-199110000-00004	B	B		C													
Sydow, DOI: 10.1164/ajrccm.149.6.8004312	C			C		C	C	C	C	C							
Kaplan, DOI:10.1186/cc1027	C			C													
Putensen, DOI:10.1164/ajrccm.164.1.2001078	C	B		C		C					C						
Varpula, doi: 10.1111/j.1399-6576.2004.00411.x	B		B	C		B					B		B				
Yoshida, DOI: 10.1213/ANE.0b013e3181bbd918	C			B		C											
Maxwell, DOI: 10.1097/TA.0b013e3181e75961	B	B	B	B		B					B			B	B		
Zhou, DOI 10.1007/s00134-017-4912-z	C	B		C				C			C						
Hirshberg, DOI: 10.1097/CCM.0000000000000343	B							B		B				B			
Ibarra-Estrada, DOI: 10.1097/CCM.0000000000005312	C			C							B		B	B		B	
Joseph, doi.org/10.36401/ISIM-20-03	C							C		C							
Ge, doi: 10.3389/fphys.2021.684927	C			C		C					B					B	C
Kucuk, doi.org/10.1016/j.bjane.2021.03.022	C							B				B	B			C	
Li, doi.org/10.1186/s13054-023-04469-8	C	C		C	C												
Zou, doi.org/10.1016/j.chest.2024.08.050																	
**Legends**																	
**A = APRV Negative Impact**	0	0	0	0	0	0	0	0	0	0	0	0	0	0	0	0	0
**B = APRV Neutral Impact**	5	5	2	2	0	2	0	2	0	1	4	1	3	3	1	2	0
**C = APRV Positive Impact**	10	1	0	10	1	4	1	3	1	2	2	0	0	0	0	1	1
**Negjtive Impact %**	0%	0%	0%	0%	0%	0%	0%	0%	0%	0%	0%	0%	0%	0%	0%	0%	0%
**Neutral Impact %**	33%	83%	100%	16%	0%	33%	0%	40%	0%	33%	66%	100%	100%	100%	100%	66%	0%

Oxygenation = PaO_2, O2 Delivery;P/Fi ratio; V/Q ratio

Ventilation = $PaCO_2$, physiologic dead space.

VAP = Ventilator-associated pneumonia

LM = Lung mechanics: Airway pressures and respiratory system compliance

ICU LOS = Intensive Care Unit Length of Stay

VFD = Ventilator Free Days

ARDS% - ARDS Incidence

Pressors = Vasopressors

Pneumo = Pneumothorax

Open Lung = Measured by Electrical Impedance Tomography

UO = Urine Output

Hemo = Hemodynamics: Blood pressures, cardiac output

Table S3 Legend

In 16 published adult clinical trials, 17 parameters were measured, with study durations ranging from 30 minutes to the duration of hospital stay. The number of studies measuring the same parameter ranged from 1 to 15. The number of patients per study ranged from 12 to 138. There was considerable variation in the methods used to set and adjust the APRV mode, and none were precisely aligned with those recommended in these guidelines.

No parameter in any of the clinical studies showed a negative effect using APRV. Oxygenation was the most frequently measured parameter, with APRV having a positive (67%) or neutral (33%) effect on oxygenation at a lower peak airway pressure (Ppeak) (83%) and a positive effect on hemodynamics (67%). There were no significant differences in arterial pH, ARDS incidence, mortality, pneumothorax, ventilator-associated pneumonia (VAP), or chest X-rays. APRV positively impacted ventilation, lowered sedation, lowered vasopressor use, increased ventilator-free days, and reduced ICU length of stay. Additionally, APRV improved lung opening, increased urine output, and reduced paralytic use in single-center studies.
