# Supplementary material for: Expert recommendations for setting and adjusting airway pressure release ventilation based on clinical experience and basic science evidence
Source: Front Med (Lausanne). 2026 Feb 3;13:1741129. doi: 10.3389/fmed.2026.1741129 (PMC12909506; doi:10.3389/fmed.2026.1741129)
Supplement: Supplementary file 5 [file Supplementary_file_5.pdf]

Supplementary File 5: Adult animal models using airway pressure release ventilation (APRV)

Trial	Year	Settings P_{High} T_{High} P_{Low} T_{Low}	Principal Findings and APRV Score
APRV vs. IPPV+PEEP OA on 10 Dogs for 2hrs. Stock, PMID: 3568710	1987	1;10 NL 1;2 NL	Score: C – Oxy, Vent, LuMec<br>Score: B – Hemo
APRV vs. CPPV OA on 10 dogs for 1.5hrs. Rasanen, DOI: 10.1378/chest.93.5.911	1991	1;10 NL 1;0 1;1.5	Score: C – Oxy, Vent, Hemo
APRV vs. CMV+LV_T LAV on 21 rabbits for 4hrs. Matsuzawa, DOI:10.1097/EJA.0b013e328333c2b0	2010	1;20 1;2.9 1;5 1;0.15	Score: C - Oxy, Inflam, W/D.<br>Score: B – Hemo.
APRV, HFOV, LV_T, RM+OP Tween, on 22 pigs for 6hrs Albert, doi:10.1016/j.jss.2010.10.022	2011	1;30 1;5 1;0 1;0.2	Score: C – Inflam.<br>Score: B – Hemo, Oxy, HistP
APRV vs. CV NPV PS+I/R on 8 pigs for 48hrs Roy, DOI: 10.1097/TA.0b013e31825c7a82	2012	TCAV	APRV prevented the development of ARDS<br>Score: C – Oxy, HistP, W/D, SfP, Pulmonary Inflam, LuMec<br>Score B – Systemic Inflam
APRV vs. CMV normal rats for 6hrs. Emr, doi:10.1001/jamasurg.2013.3746	2013	TCAV	APRV prevented the development of ARDS<br>Score: C – Oxy, SfP, AF<br>Score: B – Hemo, Pulmonary Inflam, VPerm
APRV vs. VC T/HS on 9 rats for 6hrs. Roy, DOI: 10.1097/SHK.0b013e31829efb06	2013	TCAV	APRV prevented the development of ARDS<br>Score: C – Oxy, VPerm, SfP, AF, AlvS<br>Score: B – Hemo, LuMec
APRV vs. VC-LV_T PS+I/R on 12 pigs for 48hrs. Roy, DOI: 10.1097/SHK.0b013e31827b47bb	2013	TCAV	APRV prevented the development of ARDS.<br>Score: C – Oxy, Hemo, LuMec, VPerm, SfP, HistP, W/D, Inflam<br>Score: B – Vent.
APRV 10, 25, 50, 75 CMV PEEP 5, 10, 16, 20, 24 on 10 rats Tw Kollisch, doi:10.1001/jamasurg.2014.1829	2014	TCAV	APRV 75 vs all groups.<br>Score: C – AlvS+AlvR in combination<br>Score: B – AlvS alone vs PEEP 16-24<br>Score: B – AlvR alone vs APRV 10-50
APRV 75 & APRV 10 CMV PEEP 5 & 16 on 10 rats Tw Kollisch, doi.org/10.1016/j.jamcollsurg.2014.09.011	2014	TCAV	APRV 75 vs all groups.<br>Score: C – AF, AlvS, AlvH
APRV vs. VC-LV_T HCL+ICP on 22 pigs for 6.5hr. Davies, DOI: 10.1097/TA.0000000000000518	2015	1;25 1;4 3;0 1;0.5	Score: B – Oxy, Vent, Hemo, LuMec, HistoP, ICP

APRV 10, 25, 50, 75<br>CMV PEEP 5, 10, 16, 20, 24<br>on 14 rats Tw<br>Kollisch, doi:10.1001/jamasurg.2015.2683	2015	TCAV	APRV 75 vs all groups.<br>Score: C – AlvH+AlvSize in combination<br>Score: B – AlvH alone vs PEEP 16-24
APRV vs. VC-LV$_T$ PS+I/R<br>on 6 pigs for 48hrs.<br>Roy, DOI: 10.1097/SHK.0b013e31827b47bb	2015	TCAV	APRV prevented the development of ARDS.<br>Score: C – Oxy, LuMec, SfP, HistoP<br>Score: B – Hemo, Inflam, W/D
APRV vs CV Ex Vivo lungs<br>on 4 porcine lungs for 4hrs<br>Mehaffery, doi.org/10.1016/j.jtcvs.2016.09.029	2017	4;NL 1;5 3;0 2;NL	Score: C – Oxy, LuMec, W/D
APRV 75 vs. APRV 25 HTw<br>on 12 pigs for 6hrs.<br>Jain, DOI 10.1186/s40635-017-0138-1	2017	1;40 1;90% 3;0; 0.6±0.2	Score: C - In Tw Tissue: Oxy, HistoP, W/D<br>Score: B – Hemo, Vent
APRV vs. VCV<br>ARDSp & ARDSesp<br>on 28 rats for 1hr.<br>Silva, DOI: 10.1097/CCM.0000000000003078	2018	TCAV	ARDSexp Score: C - HistP<br>ARDSp Score: C – Inflam, HistoP, VPerm<br>Both Injuries Score: B – LuMech, Oxy, Vent<br>.
APRV vs. VCV ARDSnet<br>on 10 fixed pig lungs<br>Mahajan, doi.org/10.1186/s40635-019-0250-5	2019	TCAV	Score: C – Mucus clearance
APRV vs. VCV pneumonia<br>on 21 rats for 1hr.<br>Magalhaes, DOI: 10.1097/CCM.0000000000004675	2021	TCAV	Score: C – Inflam, VPerm, CFU, HistoP, SfP<br>Score: B – Vent, Hemo
APRV vs. A/C-VCV<br>on 10 pigs for 48hrs<br>Chen, doi: 10.3389/fphys.2022.927507	2022	4;25 3;4-5 3;0 3,NL	Score: C – LuMech, HistoP, Vent, VPerm<br>Score: B – Oxy, EVLW
APRV 4 set to cause OD,<br>RD, both, or neither (TCAV)<br>HTw on 40 pigs for 6hrs.<br>Ramcharran, doi:10.1152/japplphysiol.00312.2022	2022	TCAV	Score: C - Oxy, LuMech, HistoP, W/D, VPerm<br>Inflam<br>Score: B – Hemo
APRV vs. VCV ARDSnet<br>saline lavage on 21 pigs for 48hr<br>Ma, doi.org/10.1186/s12890-022-02238-x	2022	4;25 3;4 3;0 3;0.4	Score: C – LuMech, EIT<br>Score: B – Hemo, Oxy, Vent, Inflam
APRV 4 set to cause OD,<br>RD, both, or neither (TCAV)<br>HTw on 40 pigs for 6hrs.<br>Gaver, doi.org/10.1073/pnas.2419374122	2025	TCAV	Score: C - Oxy, LuMech, HistoP, W/D, VPerm<br>Inflam, DissEn<br>Score: B – Hemo

Numerical code for the 4 APRV settings: The first # are the setting method codes below (1-6). The second # is the actual value in cmH_2O (P_{High}, P_{Low}) or seconds (T_{High}, T_{Low}).

Example:
If the P_{High} is arbitrarily set (1) at (12) cmH_2O, it would be **1;12**. If T_{Low} were set at 50%-75% of $F_{PE,}$ resulting in a 0.5sec expiratory duration, it would be **3; 0.5**.

Settings methods numbering system:
P_{High} - 1) arbitrarily set in cmH_2O, 2) set to optimize PaO_2/FiO_2 with cardiovascular compromise, 3) set based on tidal volume (V_T), 4) set based on conventional ventilation peak or plateau airway pressure, or 5) titrated with PV-curve.

T_{High} - 1) arbitrarily set in seconds, 2) based on SB rate, or 3) adjusted to maintain $PaCO_2$, 4) a percentage of each breath, for example, 90% of each breath at T_{High}.

P_{Low} - 1) arbitrarily set in cmH_2O, 2) titrated with PV-curve, 3) set at 0 cmH_2O, or 4) set based on tidal volume V_T.

T_{Low} - 1) arbitrarily set in seconds, 2) set at 75% of peak expiratory flow (P_{EF}), 3) set at 50%-75% of P_{EF}, 4) set at 25%-75% of P_{EF}, 5) adjusted to a targeted V_T, or 6) adjusted to a targeted $PaCO_2$.

TCAV = Time-controlled Adaptive Ventilation method to set APRV. The settings are closest to those suggested by these Guidelines. [DOI:10.1097/MCC.0000000000001123]

NL = Settings not listed or unclear

Principal Findings Score summary: APRV vs other ventilation groups: **A)** Negative Impact, **B)** Neutral Impact, and **C)** Positive Impact.

Injury Models Code

OA = Oleic Acid; **LAV** = Saline lung lavage; **Tw** = Tween lung lavage; **HTw** - Heterogeneous Tween lavage; **T/HS** = Trauma and Hemorrhagic Shock; Peritoneal sepsis (PS) + Gut ischemia/reperfusion (I/R) (**PS+I/R**); Hydrochloric Acid lung lavage (**HCL**); Increase cerebral pressure (**ICP**); **ARDSp** = Primary ARDS endotoxin lung instillation; **ARDSexp** = extra pulmonary ARDS endotoxin intraperitoneally.

Ventilator Modes and Methods Code as listed in each publication

APRV 10 = T_{Low} set at 10% of the peak expiratory flow (F_{PE} x 0.10); APRV 25 = T_{Low} set at 25% of the peak expiratory flow (F_{PE} x 0.25); APRV 50 = T_{Low} set at 50% of the peak expiratory flow (F_{PE} x 0.50); APRV 75 = T_{Low} set at 75% of the peak expiratory flow (F_{PE} x 0.75 - the TCAV method); Low tidal volume (ARDSnet Protocol) ventilation = LV_T; PEEP = Positive end-expiratory pressure; VCV = Volume cycled ventilation; IPPV = Intermittent positive-pressure ventilation; PPV = Positive pressure ventilation; PCV = Pressure Controlled Ventilation; A/C-VCV = Volume assist-controlled ventilation; CV = Conventional ventilation; CPPV = Conventional positive pressure ventilation; VC-IRA = Volume-controlled Inverse Ratio; PC-IRA = Pressure-controlled Inverse Ratio; PS-SIMV = Pressure Support with synchronized intermittent ventilation; ARDSnet LV_T = Low tidal volume ventilation using the ARDSnet protocol; HFOV = High-frequency oscillatory ventilation; RM+OP = Recruitment+Optimized PEEP; NPV = Nonprotective ventilation

Parameters Measured Code

Alveolar ovedistension (**OD**)

Alveolar Recruitment/Derecruitment (**RD**)

Hemodynamics (**Hemo**) = Blood pressures, cardiac output, lactate, vasopressors

Lung mechanics (**LuMec**) = Airway pressures and respiratory system compliance

Oxygenation (**Oxy**) = PaO_2, Oxygen Delivery; Venous admixture, PaO_2/FiO_2 ratio; Ventilation/Perfusion ratio.

Ventilation (**Vent**) = $PaCO_2$, respiratory acidosis, physiologic dead space.

Pulmonary edema = Lung tissue Wet/Dry weight ratio (**W/D**) or extravascular lung water (**EVLW**)

Microenvironment: Histopathology (**HistP**); Alveolar Inflation (**AF**); Alveolar stability or microstrain (**AlvS**); Alveolar Recruitment (**AlvR**), Alveolar Homogeneity (**AlvH**); Alveolar Size (**AlvSize**)

Inflammation (**Inflam**) = High-mobility group box-1 (**HMGB1**); cytokines; white blood cells

Vascular permeability increase (**VPerm**): bronchoalveolar lavage (**BALF**) proteins: E-cadherin, VCAM-1, amphiregulin

Pulmonary surfactant proteins (**SfP**): Pulmonary surfactant dysfunction

Brain ischemia (**Cisch**): Cerebral micro-dialysis

Mucus clearance (**Mc**) = Movement of mucus retrograde toward the trachea

Colony-forming units (**CFU**) = Blood bacteria load counts on a Petri dish

Electrical Impedance Tomography (**EIT**) = Improved lung volume or stability

Dissipated energy during alveolar recruitment (**DissEn**)

Principal Findings Score summary: APRV vs other ventilation groups: **A)** Negative Impact, **B)** Neutral Impact, and **C)** Positive Impact.

Table S4	Oxygenation	Ventilation	Hemodynamics	LuMec	Inflammation	HistoP	SfP	W/D	EVLW	Vperm	EIT	Alveolar Inflation	AlvS	AlvH	AlvR	AlvH+AlvSize	Mc Clearance	DissEN	ICP	CFU
**Publications**																				
Stock, PMID: 3568710	C	C	B	C																
Rasanen, DOI: 10.1378/chest.93.5.911	C	C	C																	
Matsuzawa, DOI:10.1097/EJA.0b013e328333c2b0	C		B		C			C												
Albert, doi:10.1016/j.jss.2010.10.022	B		B		C	B														
Roy, DOI: 10.1097/TA.0b013e31825c7a82	C			B	B	B	B	B												
Emr, doi:10.1001/jamasurg.2013.3746	C		B		B		C			B		C								
Roy, DOI: 10.1097/SHK.0b013e31829efb06	C		B	B			C					C	C							
Roy, DOI: 10.1097/SHK.0b013e31827b47bb	C	B	C	C	C	C	C	C												
Kollisch, doi:10.1001/jamasurg.2014.1829													B		B	C				
Kollisch, doi.org/10.1016/j.jamcollsurg.2014.09.011												C	C	C						
Davies, DOI: 10.1097/TA.0000000000000518	B	B	B	B		B													C	
Kollisch, doi:10.1001/jamasurg.2015.2683														B		C				
Roy, DOI: 10.1097/SHK.0b013e31827b47bb	C		B	C	B	C	C	B												
Mehaffery, doi.org/10.1016/j.jtcvs.2016.09.029	C			C				C												
Jain, DOI 10.1186/s40635-017-0138-1	C	B	B			C		C												
Silva, DOI: 10.1097/CCM.0000000000003078	B	B		B	C	C				C										
Mahajan, doi.org/10.1186/s40635-019-0250-5																	C			
Magalhaes, DOI: 10.1097/CCM.0000000000004675	B	B	B		C	C	C		B	C										C
Chen, doi: 10.3389/fphys.2022.927507	B	C		C		C			B											
Ramcharran, doi:10.1152/japplphysiol.00312.2022	C		B	C	C	C		C		C										
Ma, doi.org/10.1186/s12890-022-02238-x	B	B	B	C	B						C									
Gaver, doi.org/10.1073/pnas.2419374122	C		B	C	C	C		C		C								C		
**Legends**																				
**A = Negative Impact**	0	0	0	0	0	0	0	0	0	0	1	0	0	0	0	0	0	0	0	0
**B = Neutral Impact**	6	6	12	4	4	3	1	2	2	1	0	0	1	1	1	0	0	0	0	0
**C = Positive Impact**	12	3	2	8	7	8	5	6	0	4	0	3	2	1	0	2	1	1	1	1
**Negative Impact %**	0%	0%	0%	0%	0%	0%	0%	0%	0%	0%	0%	0%	0%	0%	0%	0%	0%	0%	0%	0%
**Neutral Impact %**	33%	67%	85%	33%	36%	27%	17%	25%	100%	20%	0%	0%	33%	50%	100%	0%	0%	0%	0%	0%
**Positive Impact %**	67%	33%	14%	67%	64%	73%	83%	75%	0%	80%	100%	100%	67%	50%	0%	100%	100%	100%	100%	100%

Table S4 Legend

Twenty-one parameters were measured across 22 published adult animal studies, with study durations ranging from 1 to 48 hours. The number of studies measuring each parameter ranged from 1 to 18. The number of animals used ranged from 4 to 40. Multiple methods were used in 9 studies to set and adjust the APRV mode. However, the methods most consistent with the guidelines outlined in this paper were used in 13 studies (i.e., the TCAV method).

APRV did not negatively affect any parameter in any of the 22 studies. The most studied parameter was oxygenation. APRV showed 1) a positive (67%) or neutral (33%) effect on oxygenation, 2) a positive effect on reducing airway pressure and improving compliance (LuMec 67%), and 3) a neutral (85%) or positive (14%) effect on hemodynamics.

Furthermore, APRV had either a positive or neutral effect on ventilation ($PaCO_2$ and arterial pH), inflammation (cytokines and white blood cells), histopathology (histoP), surfactant proteins (SfP), pulmonary edema (W/D), vascular permeability (Vperm), and alveolar heterogeneity (AlvH). No significant differences (100% neutral effect) were observed between groups regarding extravascular lung water (EVLW) and alveolar recruitment (AlvR).

Additionally, APRV positively affected lung aeration (EIT), the combined measure of alveolar heterogeneity and size (AlvH+AlvSize), mucus (Mc) clearance, dissipated energy (DissEN), increased intracranial pressure (ICP), and colony-forming units (CFU).
