# Supplementary material for: Expert recommendations for setting and adjusting airway pressure release ventilation based on clinical experience and basic science evidence
Source: Front Med (Lausanne). 2026 Feb 3;13:1741129. doi: 10.3389/fmed.2026.1741129 (PMC12909506; doi:10.3389/fmed.2026.1741129)
Supplement: Supplementary file 6 [file Supplementary_file_6.pdf]

Supplementary File 6 - Computational models analyzing time-controlled mechanical ventilation

Eight published papers analyzing the impact of ventilator time on lung mechanics using computational models were reviewed. Computational modeling clearly shows that extended inspiratory time recruits alveoli, while sufficiently brief expiratory time prevents re-collapse. Studies examining airway pressure release ventilation (APRV) demonstrate its ability to stabilize and progressively recruit the lung, thereby reducing both volutrauma and atelectrauma. A digital twin-based computational model showed that the APRV mode was superior to pressure-controlled ventilation (PCV) in reducing mechanical power and lung tissue recruitment and derecruitment in ARDS patients.

1) Bates JHT 2002. DOI: 10.1152/japplphysiol.01274.2001

Computational Model Description

The model consists of a parallel array of lung units, each comprising an alveolar compartment served by an airway that can switch between fully open and completely closed states. All the airways connect at their proximal ends to a common airway opening. The model is driven by an applied pressure waveform (P). If we focus on the slow inflations and deflations of this model, we can ignore the component of P due to airway resistance. When an airway is open, its downstream alveolar compartment inflates and deflates with P according to the well-known exponential relationship

$$V = A - Be^{-KP} \quad \text{(Eq 1)}$$

where A, B, and K are constants and V is the volume of the alveolar compartment. Each airway has a critical opening pressure, P_O, and a critical closing pressure, P_c. If the airway is closed and $P > P_O$, the airway opens with a delay determined by the rate of increase of a virtual trajectory variable x. This trajectory variable does not represent any physical quantity or process, but it is motivated by the idea that liquid bridge formation across the lumen of small airways takes a finite amount of time to manifest when P is low, and to break when P is high. When x reaches a value of 1, the airway opens. Conversely, if the airway is open and $P < P_C$, x decreases until it reaches a value of 0, at which point the airway closes. V remains fixed until the airway reopens, at which point it immediately assumes the value of V appropriate for the current value of P according to Eq. 1. The rate of change of x is proportional to the amount by which P exceeds P_O of is less than P_C. The trajectory variable x remains fixed for $P_C < P < P_O$. Distributions of values for P_O, , and the constants of proportionality for the rate of change of, x determine the quasi-static P-V behavior of the entire model.

Summary of Findings

This model incorporates a novel computational mechanism that explains how recruitment and derecruitment cause transient behavior in lung mechanics. With appropriate parameter values, the model accurately replicates the first and second P-V loops of a degassed lung, as well as the transient drops in lung compliance following a deep lung inflation. Adjusting the model parameter values appropriately replicates the increased P-V hysteresis and exaggerated post-sigh derecruitment seen in injured lungs. This model can be used to explore how different ventilation strategies can help recruit an injured lung.

2) Albert SP 2008. DOI: 10.1152/japplphysiol.90735.2008.

Mathematical Model Description

This model is an analytic version of the numerical model originally proposed by Bates and Irvin [DOI: 10.1152/japplphysiol.01274.2001]. The analytic model focuses on the dynamics of recruitment following a sudden elevation in airway pressure and, again, is based on the concept of the virtual trajectory. The model predictions were compared with measurements of lung recruitment in rats obtained using *in vivo* microscopy.

Summary of Findings

The study measured alveolar recruitment for 40 seconds at three different airway pressures (20, 30, 40 cmH_2O). Most of the recruitment occurred within the first two seconds following pressure elevation to 30 and 40 cmH_2O, and about half of the recruitment was observed at two seconds with 20 cmH_2O. The time courses of recruitment were accurately predicted by the mathematical model, supporting the hypothesis that the degree of alveolar recruitment generated by a lung inflation depends on the magnitude of the recruiting pressure and the duration of the inflation. This demonstrates that the key question regarding the use of recruitment maneuvers during ventilation of the injured lung is not whether they should be applied, but rather how often.

3) Smith BJ 2015. DOI: 10.1152/japplphysiol.00902.2014

Computational Model Description

The model consists of 768 parallel respiratory units, each comprising an elastic alveolar compartment connected to an airway that is either open or closed, all such airways being connected to a common airway junction. All alveolar compartments are identical and have a constant elastance E_{base} at low volumes (below V_{crit}) and exhibit strain stiffening at high volumes (above V_{crit}). The rate of increase in elastance above V_{crit} is determined by a rate constant E_{Fac}. The airways are either open or closed according to the virtual trajectory model, the elastance E_{RS} of the entire model at any point in time, being determined by the number of open units. The values of the parameters V_{crit}, E_{base}, and E_{Fac} were determined by fitting the model to experimental data of respiratory system elastance versus time following recruitment maneuvers.

Summary of Findings

The predictions from this model suggest that airway pressure release ventilation (APRV) is more effective at lung recruitment than low tidal volume (V_T) ventilation, without causing excessive tissue overdistension. This shows that both the timing and magnitude of the airway pressures used during mechanical ventilation influence the timing of lung recruitment, while the maximum tissue distension across the lung depends on inspiratory airway pressure. The model simulations indicate that low-V_T ventilation generally results in less intra-tidal recruitment compared to APRV.

4) Bates JHT 2020. DOI: 10.1097/CCE.0000000000000299

Computational Model Description

The lung is modeled as a single alveolar compartment that can expand in two perpendicular directions. Vertical expansion stretches the tissues of the open fraction of the lung, while horizontal expansion increases the fraction of open lung (i.e., recruitment of closed lung units). Thus, the open lung units are all distended to the same extent by alveolar pressure, but the number of open units varies over time as recruitment or derecruitment occurs. The pressure required to open a closed lung unit during inflation is higher than the pressure at which it closes during expiration. This model provides a simple representation of the recruitable lung that can be fit to continuous measurements of airway pressure and flow made during mechanical ventilation.

Summary of Findings

The model shows that repetitive alveolar collapse and expansion (RACE)-induced atelectrauma during mechanical ventilation is heavily influenced not only by airway pressure but also by the duration of the applied pressures. Simulations with the model support the common understanding that avoiding atelectrauma during conventional low tidal volume ventilation is mainly achieved by adjusting the pressure via PEEP. Conversely, preventing atelectrauma during airway pressure release ventilation (APRV) is primarily accomplished by adjusting the expiratory time (T_{Low}). Notably, the model further demonstrates that the effectiveness of APRV depends on the relative values of two time-dependent processes, represented in our model by the time constants of lung emptying and recruitment/derecruitment (R/D). An important yet often overlooked factor is that the duration of expiration is just as crucial as airway pressure levels in preventing atelectrauma. Using this model at the bedside could help optimize APRV parameters on a patient-specific basis in order to minimize RACE.

5) Cruz AF 2023. DOI: org/10.1093/milmed/usad059

Computational Model Description

The computational model includes a ventilator pressure source, a distensible breathing circuit, an endotracheal tube, and a porcine lung with recruited and derecruited zones, as well as a transitional zone capable of intratidal recruitment and derecruitment (R/D). Lung injury was simulated by altering the surface tension of each acinus in an inflation-dependent manner. The model was designed to simulate time-varying

fluctuations in gas pressure and flow throughout an anatomically structured airway tree with viscoelastic acini. For simplicity, mechanical relationships between gas pressure and flow were described using lumped-element models. The porcine lung was divided into distinct zones: recruited, derecruited, and a transitional zone capable of intratidal R/D. The mechanical properties of the consistently recruited zone were modeled as a lumped-element parallel combination of all open acini during ventilation, in series with a lumped-element model of the resistive and inertial properties of the airway segments leading to those acini. Lung injury was simulated by altering each acinus's inflation-dependent surface tension to a value randomly drawn from a uniform distribution, mimicking different levels of surfactant dysfunction.

Summary of Findings

Simulations with this anatomically based computational model of the ventilated injured lung demonstrate the confounding effects of cyclic R/D, sustained recruitment, and parenchymal strain stiffening on estimates of both global and regional elastance during airway pressure release ventilation (APRV). Increasing inspiratory airway pressures not only result in more sustained recruitment of unstable acini but also cause increased intra-tidal R/D. These simulations thus suggest that higher inspiratory pressures should be combined with shorter exhalation times to prevent an increase in intra-tidal R/D. The model also illustrates a key difference between current protective ventilation strategies and APRV: the former is tailored for a heterogeneously injured lung, while the latter recruits the entire lung over an extended period, up to hours or even days. This allows the lung to repair naturally in its fully recruited state.

6) Ma H. DOI : 10.3389/fnetp.2023.1257710

Computational Model Description

This study developed and examined a comprehensive multiscale computational model of a mechanically ventilated ARDS lung to clarify the mechanisms underlying the development and prevention of ventilator-induced lung injury (VILI). The model is based on a healthy lung model that includes realistic airway and alveolar geometry, tissue distensibility, and surfactant dynamics. Key features of the ARDS model include recruitment and derecruitment (R/D) dynamics, alveolar tissue viscoelasticity, and surfactant deficiency. This model effectively reproduces realistic pressure-volume (PV) behavior, dynamic surface tension, and time-dependent descriptions of R/D events across different ventilation scenarios.

Summary of Findings

Simulations of airway pressure release ventilation (APRV) with both short and long exhalation times (T_{Low}) show a higher incidence of R/D with long T_{Low}, despite reduced surface tension due to interfacial compression. This finding supports experimental evidence highlighting the importance of timing in protective ventilation strategies. Quantitative analysis of energy dissipation reveals that although alveolar recruitment accounts for only a small part of total energy dissipation, its spatial concentration and brief duration may significantly contribute to VILI progression due to its focal nature and higher intensity. Using

this computational framework, the model can be extended to develop personalized protective ventilation strategies that improve patient outcomes by illuminating the complex dynamics of VILI, thereby helping optimize ventilation in ARDS management.

7) Nieman GF 2023. DOI: 10.3389/fphys.2023.1287416

Computational Model Description

Using a simple computational model of the injured lung, we calculate progressive recruitment and re-collapse. We represented the lung as a single alveolar compartment that can expand in two orthogonal directions. Vertical expansion corresponds to distension of the open lung, while horizontal expansion corresponds to an increase in the open lung fraction.

Summary of Findings

We demonstrated that airway pressure release ventilation (APRV) can slowly open even the most recalcitrant alveoli with extended periods of high inspiratory pressure, while reducing alveolar re-collapse through the use of brief expirations. These processes together comprise a ratchet mechanism by which the lung is progressively recruited, similar to the way the newborn lung is aerated during a series of cries, albeit over longer time scales.

7) Bates JHT 2024. DOI: org/10.1186/s13054-024-05112-w

Computational Model Description

A mathematical model of respiratory system mechanics, which includes a volume-dependent elastance term, was fitted to 10-s airway pressure-flow epochs collected in pigs every hour for 6 hours after Tween injury, during both expiration and inspiration. We also continuously measured respiratory system impedance between 5 and 19 Hz during inspiration at the same time points, from which we derived a time course for respiratory system resistance.

Summary of Findings

The model further supports the hypothesis that sufficiently short expiratory durations protect against repetitive alveolar collapse and expansion (RACE)-induced atelectrauma because they do not allow sufficient time for derecruitment during expiration. This suggests a way to tailor mechanical ventilation settings to individual patients.

8) Joy W 2025. DOI: 10.1097/CCM.0000000000006885

Computational Model Description

Digital twins based on a computational model of the human cardiopulmonary system were matched to patients with acute respiratory distress syndrome (ARDS) on pressure-controlled ventilation (PCV) mode.

The aim was to calculate indices of ventilator-induced lung injury (VILI) in the PCV mode and compare them with results from typical fixed and time-controlled adaptive methods used to set the airway pressure release ventilation (APRV) mode in the same patients. Global optimization algorithms were used to determine the optimal settings for each mode based on VILI indices, while ensuring adequate gas exchange. Data included ventilator settings and arterial blood gas (ABG) measurements from 98 patients on PCV. The patients were selected solely based on the availability of data necessary for modeling, including ABGs and ventilator settings.

Summary of Findings

The time-controlled adaptive method for setting APRV reduced mechanical power (MP) by 32% and tidal alveolar recruitment/derecruitment (RD) by 34% compared with PCV mode, with only moderate increases in $PaCO_2$ that could have been minimized by shortening inspiratory time (T_{High}). Driving pressure, tidal volume, and lung stress/strain remained similar in both modes. The authors conclude that using digital twins can help identify lung-protective ventilation modes and methods for setting ventilation modes, which may be useful for guiding strategies in randomized controlled trials.
