# Supplementary material for: Expert recommendations for setting and adjusting airway pressure release ventilation based on clinical experience and basic science evidence
Source: Front Med (Lausanne). 2026 Feb 3;13:1741129. doi: 10.3389/fmed.2026.1741129 (PMC12909506; doi:10.3389/fmed.2026.1741129)
Supplement: Supplementary file 7 [file Supplementary_file_7.pdf]

Supplementary File 7 - Statistical and Meta-analyses on studies using airway pressure release ventilation (APRV)

Table S6	Ppeak	Oxy	MAP	CRS	Pneumo	ReqIRT	RI-ARDS	VFD	ICU LOS	TPS	H-Mortality	28-Mortality	AC-Mortality
**Meta- and Statistical-Analysis Description**													
Andrews PL, 2013							C			C	C		
DOI: 10.1097/TA.0b013e31829d3504													
16 clinical trials on 66,199 surgical ICU patients at high risk of													
developing ARDS using the standard or care mechanical													
ventilation vs. 231 APRV patients using trhe TCAV method													
applied immediately post-surgery before ARDS development.													
Carsetti A. 2019		B	C		B				C		C		
doi.org/10.1186/s13613-019-0518-7													
5 RCTs on 330 ARDS patients comparing APRV with CMV													
Lim J. 2019		C			B	B							C
DOI: 10.1097/CCM.0000000000003972													
7 RCTs on 412 ARDS patients comparing APRV to any													
ventilation model													
Sun X. 2019	C												B
doi.org/10.1097/MD.0000000000018586													
14 Clinical trials on 2096 patients comparing APRV													
to any CMV mode and method													
Zhong X. 2020		C	C	C	B			C	C		C		
doi: 10.21037/atm-20-6917													
7 RCTs on 405 ARDS patients comparing APRV with													
low tidal volume ventilation													
Chen C. 2021	C	B										C	
doi.org/10.21037/apm-21-747													
6 RCTs on 360 ARDS patients comparing APRV with													
with all methods of CMV													
Othman F. 2021		C							C				B
DOI 10.4103/atm.ATM_475_20													
6 clinical trials on 375 ARDS patients comparing APRV with													
other conventional mechanical ventilation modes													
Roshdy A. 2023		C			B			B	B				B
DOI: 10.1177/08850666221109779													
7 RCT on 354 CARDS patients comparing APRV with CMV													
**Negitive Impact = A**	**0**	**0**	**0**	**0**	**0**	**0**	**0**	**0**	**0**	**0**	**0**	**0**	**0**
**Neutral Impact = B**	**0**	**2**	**0**	**0**	**0**	**1**	**0**	**1**	**1**	**0**	**0**	**0**	**3**
**Positive Impact = C**	**2**	**4**	**2**	**1**	**3**	**0**	**1**	**1**	**3**	**1**	**3**	**1**	**1**
**Negitive Impact %**	**0%**	**0%**	**0%**	**0%**	**0%**	**0%**	**0%**	**0%**	**0%**	**0%**	**0%**	**0%**	**0%**
**Neutral Impact %**	**0%**	**33%**	**0%**	**0%**	**100%**	**100%**	**0%**	**50%**	**25%**	**0%**	**0%**	**0%**	**75%**
**Positive Impact %**	**100%**	**67%**	**100%**	**100%**	**0%**	**0%**	**100%**	**50%**	**75%**	**100%**	**100%**	**100%**	**25%**

Principal Findings Score summary: APRV vs other ventilation groups: **A)** Negative Impact, **B)** Neutral Impact, and **C)** Positive Impact.

Abbreviations

Ppeak = Peak airway pressure

Oxy = Oxygenation status

MAP = Mean arterial pressure

Pneumo = Pneumothorax

ReqIRT = The requirement to initiate rescue treatment such as inhaled vasodilators, prone positioning, and ECMO

RI-ARDS = Reduced ARDS incidence

VFD = Ventilator-free days

ICU LOS = Intensive care unit length of stay

TPS = Therapy Performance Score

H-Mortality = In-hospital mortality

28-Mortality = 28-day mortality

AC-Mortality = All cause mortality

RCT = Randomized Controlled Trials

TCAV = Time Controlled Adaptive Ventilation Method to set the APRV mode

ARDS = Acute Respiratory Distress syndrome

CARDS = Covid Acute Respiratory Distress syndrome

CMV = Conventional mechanical ventilation

Table S6 Legend

Thirteen parameters were assessed across eight published statistical and meta-analyses that included prospective and retrospective observational cohort studies and randomized controlled trials (RCTs). The number of studies evaluating the same parameter ranged from 1 to 6, and the number of patients ranged from 330 to 66,199. Most studies did not differentiate the methods used to set and adjust the APRV mode, except for one. In this study, the TCAV method for setting the APRV mode in a single hospital was compared to standard mechanical ventilation across 16 other surgical intensive care units (SICUs). [DOI: 10.1097/TA.0b013e31829d3504]

The outcomes were graded as **A** = Statistically Inferior; **B** = Statistically Neutral; and **C** = Statistically Superior, as reported by the authors in each of the papers analyzed. APRV was not statistically inferior in any parameter. A summary of the findings is as follows: Most studies assessed oxygenation with APRV and reported a statistically positive effect (67%) or a neutral effect (33%). APRV had a statistically positive impact on peak airway pressure (Ppeak 100%), mean arterial pressure (MAP 100%), respiratory system compliance (C_{RS} 100%), ARDS incidence (RI-ARDS 100%), ICU length of stay (ICU LOS 100%), Therapy Performance Score (TPS 100%), in-hospital mortality, and 28-day mortality (all 100%). TPS is calculated with the formula: TPS = %mortality x %ARDS / mean Injury Severity Score (ISS). APRV had a

positive (25%) or neutral (75%) effect on all-cause mortality (AC-Mortality). There was no statistically significant difference in pneumothorax risk between APRV and other ventilation groups (neutral 100%).
